# Supplementary material for: Sterile triggers drive joint inflammation in TNF‐ and IL‐1β‐dependent mouse arthritis models
Source: EMBO Mol Med. 2023 Sep 11;15(10):e17691. doi: 10.15252/emmm.202317691 (PMC10565626; doi:10.15252/emmm.202317691)
Supplement: Supplementary file 1 — Appendix [file EMMM-15-e17691-s012.pdf]

Appendix

Table of contents

- Appendix Figure S1: Phenotyping the TNF^{emARE} model: evaluating affected organs……1
- Appendix Figure S2: Gut phenotype of the TNF^{emARE} mouse model……………………………2
- Appendix Figure S3: Early onset of gut and joint disease in $TNF^{emARE/ARE}$ mice…………..3
- Appendix Figure S4: Absolute cell counts and gating strategy of small intestine flow cytometry………4
- Appendix Figure S5: Phenotyping the $A20^{myel\text{-}KO}$ model: evaluating affected organs….6
- Appendix Table S1: Histopathological ileitis scoring………………………………………………..7
- Appendix Table S2: Histopathological peripheral musculoskeletal disease scoring…….7
- Appendix Table S3: Histopathological axial musculoskeletal disease scoring…………….7

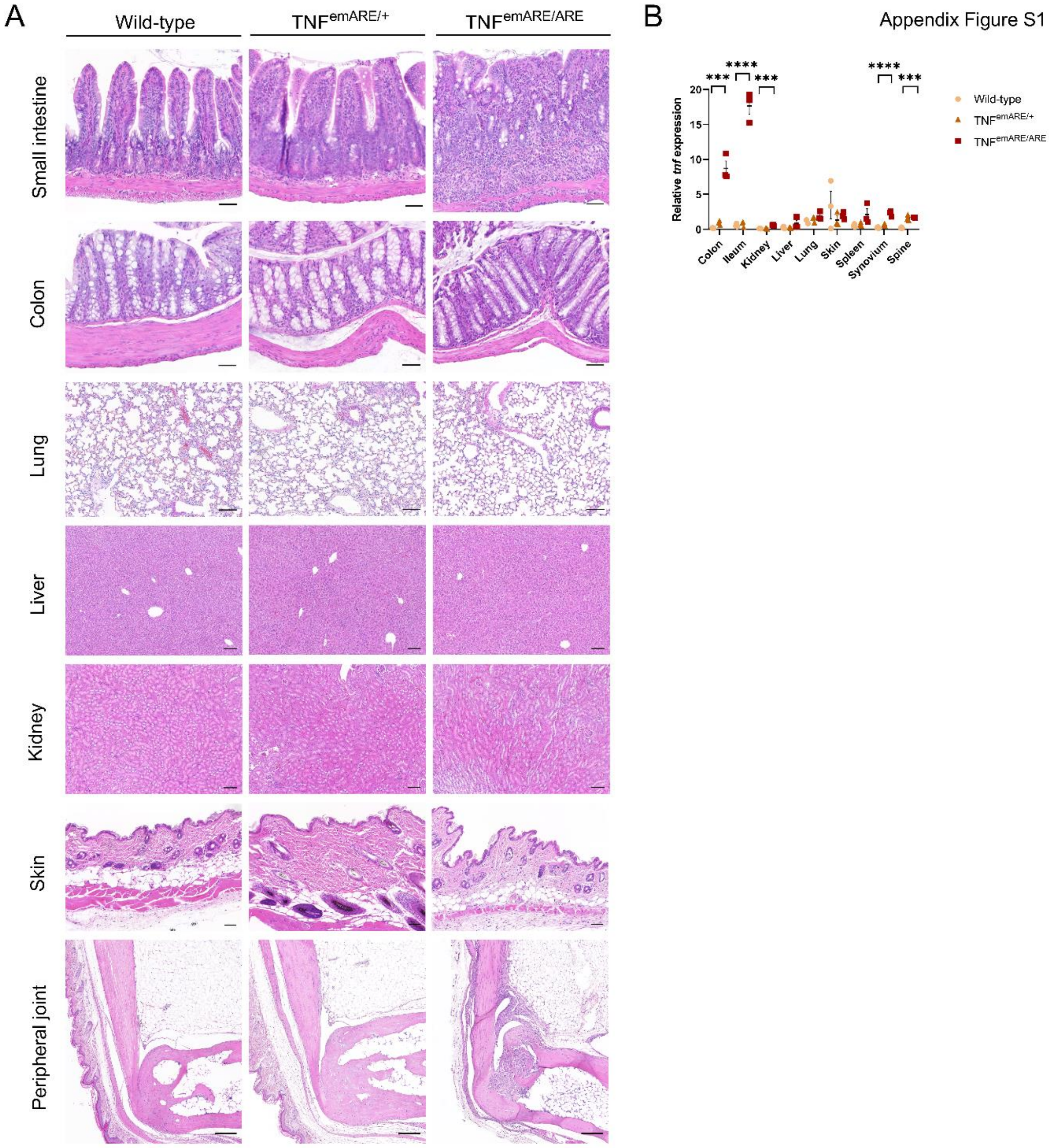

Figure S1: Phenotyping the TNF^{emARE} model: evaluating inflammation in multiple organs. (A) Histological evaluation of inflammation in sections of small intestine (ileum), colon, lung, liver, kidney, skin and peripheral joint (ankle) of wild-type, TNF$^{emARE/+}$ and TNF$^{emARE/ARE}$ mice (15-20 weeks old). (Scale bars colon, ileum, skin: 50 μm; kidney, liver, lung: 100 μm; ankle: 200 μm) (B) qPCR data on multiple tissues of TNF^{emARE} mice to evaluate expression of the *tnf* gene (15-20 weeks old mice) (n=3/genotype for every tissue).

Data information: For (B), data are represented as Mean +/- SEM, n=biological replicates, one-way ANOVA test used with Tukey's multiple comparisons test for every organ. ns= p-value > 0.05, * = p-value ≤ 0.05, ** = p-value ≤ 0.01, *** = p-value ≤ 0.001, **** = p-value ≤ 0.0001. Only significant differences between wild-types and homozygotes is shown.

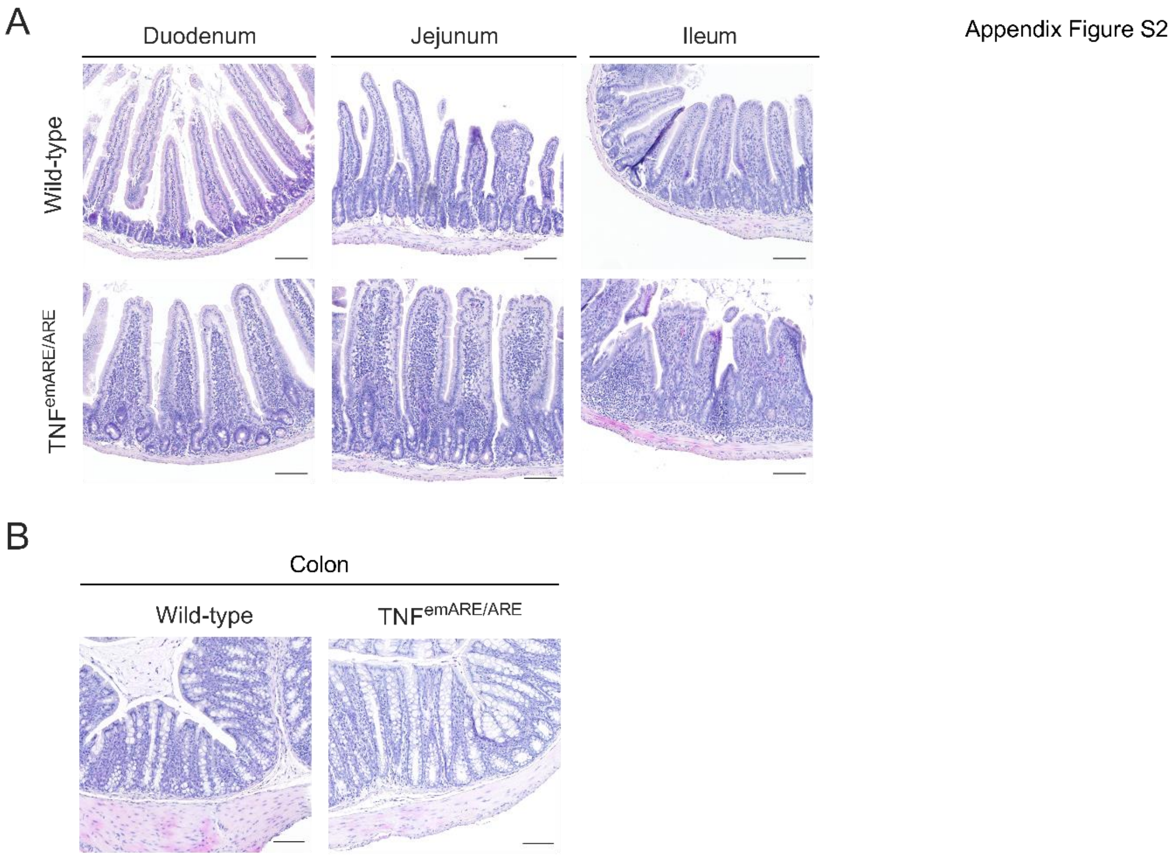

Figure S2: Gut phenotype of TNF^{emARE} mice. (A) Histological H&E stained sections of duodenum, jejunum, ileum and (B) colon of SPF wild-type and TNF$^{emARE/ARE}$ mice. (Scale bars: 100 µm). B) *il-1β* qPCR performed on multiple tissues in A20$^{myel\text{-}KO}$ and WT mice (20 weeks old) (n=3/genotype).

Data information: For (A, B), data are represented as Mean +/- SEM, n=biological replicates, one-way ANOVA test used with Tukey's multiple comparisons test for every organ. ns= p-value > 0.05, * = p-value ≤ 0.05, ** = p-value ≤ 0.01, *** = p-value ≤ 0.001, **** = p-value ≤ 0.0001.

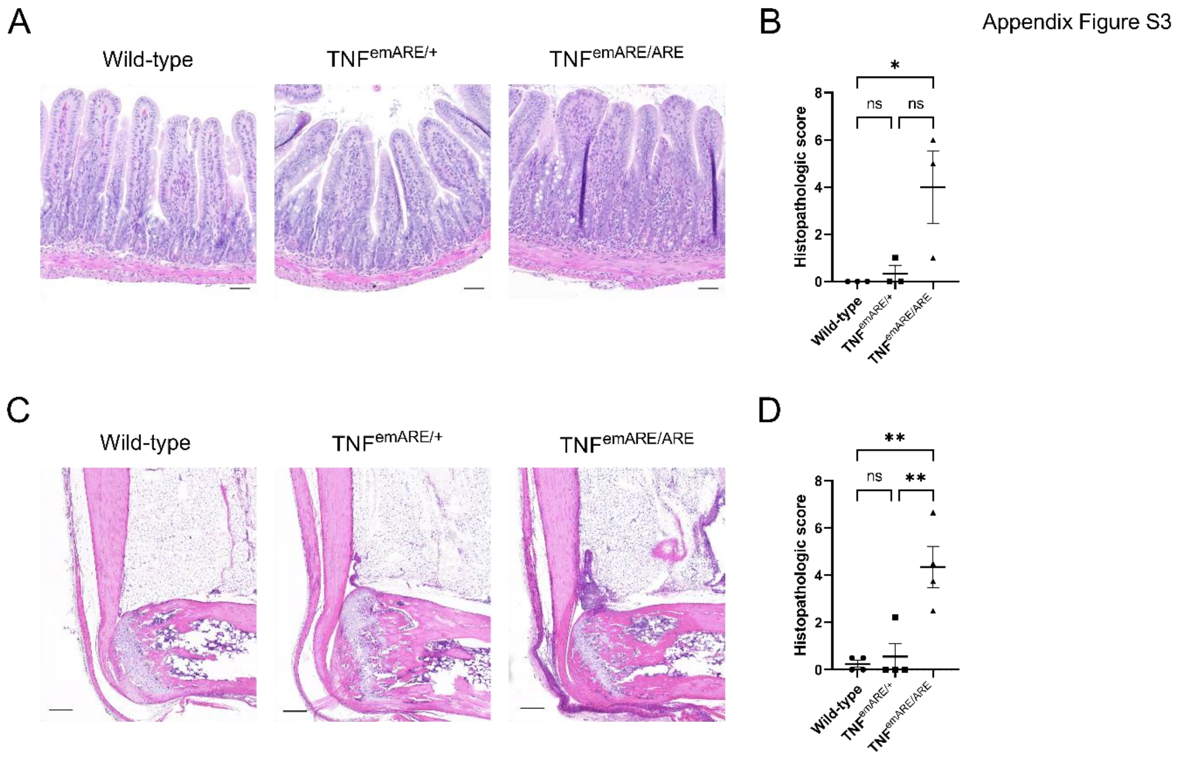

Figure S3: Early onset of gut and joint disease in TNF$^{emARE/ARE}$ mice. (A) Histological H&E stained ileal sections of 5-6 w/o SPF TNF^{emARE} mice. (Scale bars: 50μm) (B) Histopathological scoring for ileal disease in young (5-6 w/o) wild-type, TNF$^{emARE/+}$ and TNF$^{emARE/ARE}$ mice (n=3/genotype) (C) H&E stained ankle sections of young SPF TNF$^{emARE/ARE}$ mice (5-6 w/o). (Scale bars: 200μm) (D) Histopathologic scoring of musculoskeletal disease in 5-6 w/o wild-type (n=4), TNF$^{emARE/+}$ (n=4) and TNF$^{emARE/ARE}$ mice (n=4).

Data information: For graphs (B, D), data are represented as Mean +/- SEM, n=biological replicates. For graph (B), one-way ANOVA test was used with Tukey's multiple comparisons test. For graph (D), one-way ANOVA test was used with Holm-Šídák's multiple comparisons test. ns= p-value > 0.05, * = p-value ≤ 0.05, ** = p-value ≤ 0.01, *** = p-value ≤ 0.001, **** = p-value ≤ 0.0001.

Appendix Figure S4

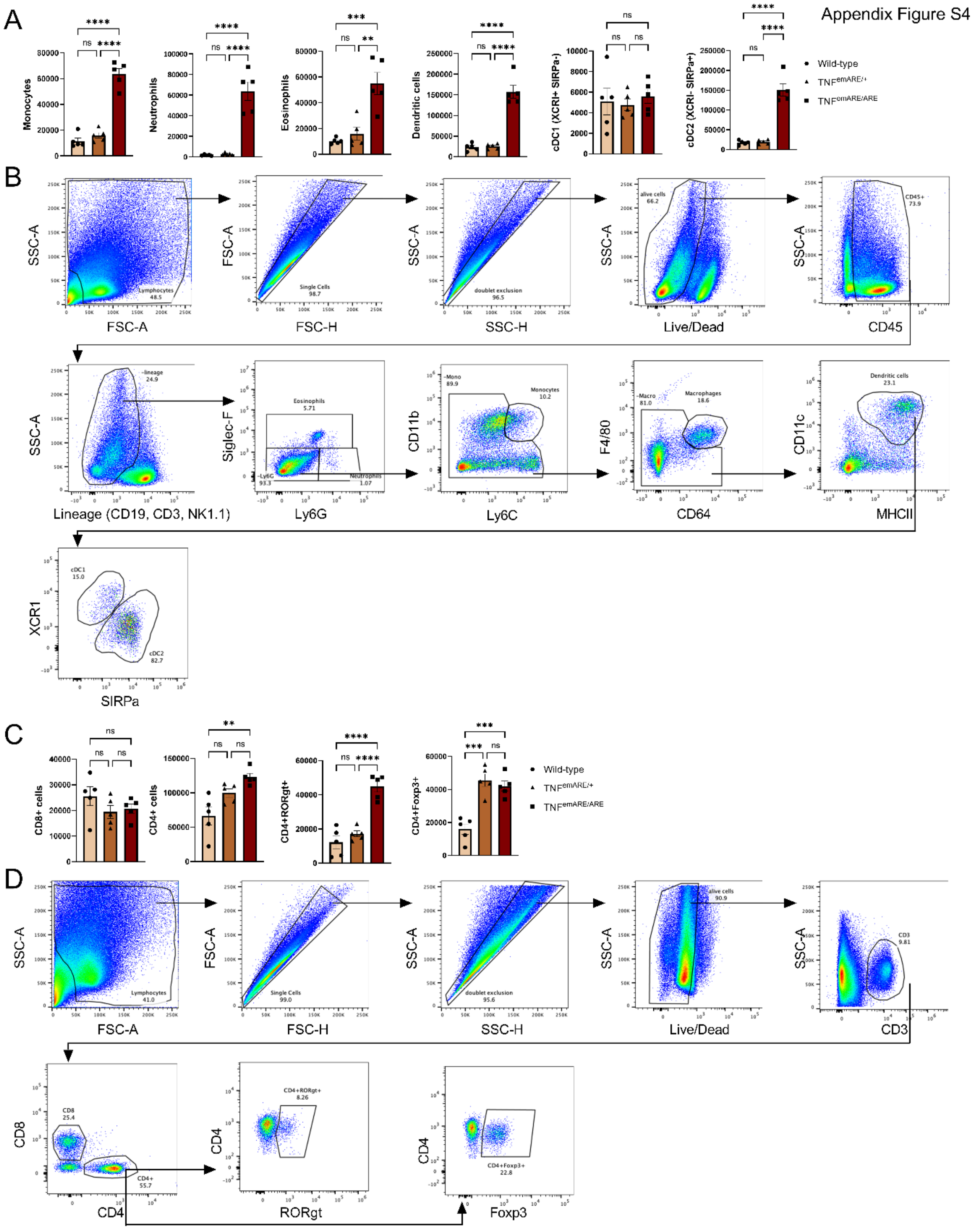

Figure S4: Absolute cell counts and gating strategy of small intestinal flow cytometry analysis. (A) Absolute cell counts of monocytes, neutrophils, eosinophils, dendritic cells, cDC1 and cDC2 cells in small intestinal lamina propria of wildtype, heterozygote and homozygote TNF^{emARE} mice. (n=5 mice/genotype) (B) Gating strategy of flow cytometry analysis of small intestinal lamina propria myeloid cells. (C) Absolute cell counts of CD8+, CD4+, Th17 and Treg cells in small intestinal lamina propria of wild-type, heterozygote and homozygote TNF^{emARE} mice. (n=5 mice/genotype) (D) Gating strategy of flow cytometry analysis of small intestinal lamina propria T cells.

Data information: For (A, C), data are represented as Mean +/- SEM, n=biological replicates, one-way ANOVA test used with Tukey's multiple comparisons test. ns= p-value > 0.05, * = p-value ≤ 0.05, ** = p-value ≤ 0.01, *** = p-value ≤ 0.001, **** = p-value ≤ 0.0001.

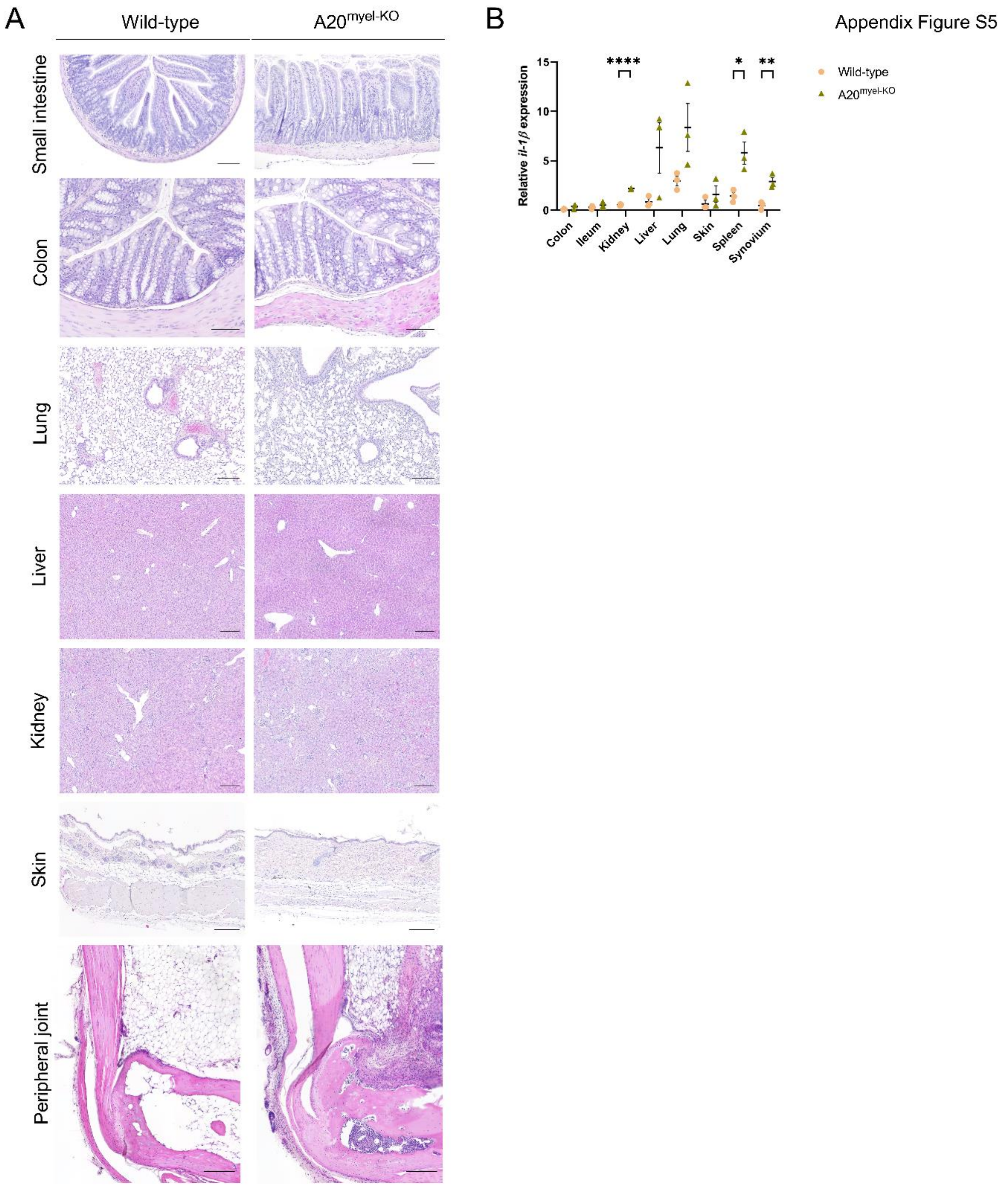

Figure S5: Phenotyping the A20$^{myel\text{-}KO}$ model: evaluating inflammation in multiple organs. Histological evaluation of inflammation in sections of small intestine, colon, lung, liver, kidney, skin and peripheral joint of wild-type versus A20$^{myel\text{-}KO}$ mice (20 weeks old). (Scale bars colon, ileum: 100 μm; kidney, liver, lung, skin, ankle: 200 μm). (B) qPCR data on multiple tissues of A20$^{myel\text{-}KO}$ mice to evaluate expression of the *il-1β* gene (20 weeks old mice) (n=3/genotype for every tissue).

Data information: For (B), data are represented as Mean +/- SEM, n=biological replicates, two-tailed unpaired t-test used for every organ. ns= p-value > 0.05, * = p-value ≤ 0.05, ** = p-value ≤ 0.01, *** = p-value ≤ 0.001, **** = p-value ≤ 0.0001.

Tables

Goblet cell count	- Normal, no GC loss (0)<br>- Minimal GC loss: loss of 1-30% (1)<br>- Clear GC loss: loss of 30-60% (2)<br>- Severe GC loss: loss of 60-90% (3)<br>- Maximal GC loss: loss of > 90% (4)
Immune cell infiltration	- Normal, no immune cell infiltration (0)<br>- Lamina propria immune cell infiltration (1)<br>- Mucosal infiltration: lamina propria and villi (2)<br>- Submucosal infiltration (3)<br>- Transmural infiltration (4)
Villus architecture	- Normal villus architecture (0)<br>- Minimal villus blunting (1)<br>- Blunted villi, only 50% of crypt remained (2)<br>- Blunted villi, only 25% of crypt remained (3)<br>- Maximal blunting, complete loss of villus-crypt architecture (4)

Appendix Table S1: Histopathological ileitis scoring

Immune cell infiltration	- immune cell infiltration in 0-15% of intervertebral discs or in the longitudinal ligament at the intervertebral disc level (0)<br>- immune cell infiltration in 15-50% of intervertebral discs or in the longitudinal ligament at the intervertebral disc level (1)<br>- immune cell infiltration in 50-90% of intervertebral discs or in the longitudinal ligament at the intervertebral disc level, or mild inflammation at the level of all discs (2)<br>- severe immune cell infiltration in all intervertebral discs/severe immune cell infiltration in the longitudinal ligament (3)

Appendix Table S2: Histopathological axial musculoskeletal disease scoring

Cuboidal joint:	- Immune infiltrates in synovium (0-2)<br>- Immune infiltrates in fat pad (0-2)<br>- Immune infiltrates in joint space (0-2)<br>- Immune infiltrates in cartilage (0-2)
Calcaneus:	- Bone erosion (0-2)<br>- Bone marrow edema (0-2)
Achilles tendon and Kargers' fat pad:	- Tendonitis (0-2)<br>- Immune infiltrates in synovium (0-2)<br>- Immune infiltrates in Kargers' fat pad (0-2)<br>- Immune infiltrates in joint space (0-2)

Appendix Table S3: Histopathological peripheral musculoskeletal disease scoring
