# Supplementary material for: Sterile triggers drive joint inflammation in TNF‐ and IL‐1β‐dependent mouse arthritis models
Source: EMBO Mol Med. 2023 Sep 11;15(10):e17691. doi: 10.15252/emmm.202317691 (PMC10565626; doi:10.15252/emmm.202317691)
Supplement: Supplementary file 2 — Expanded View Figures PDF [file EMMM-15-e17691-s007.pdf]

Expanded View Figures

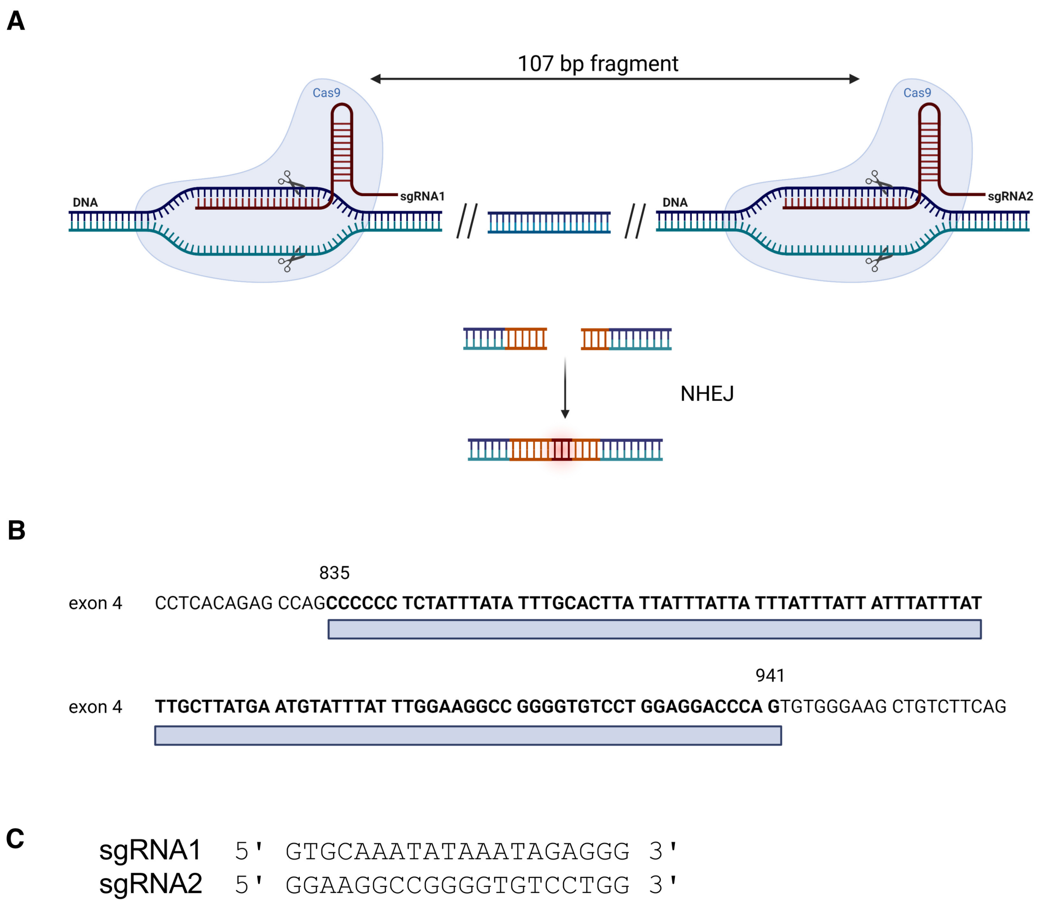

Figure EV1. Generation of a TNF-driven inflammation model by targeting the AU-rich element of the Tnf gene.

A Schematic overview of a deletion of a 107 bp fragment by CRISPR-Cas9 technology, followed by non-homologous end-joining (NHEJ).
B A 107-bp fragment was deleted in the 3′UTR region (exon 4) of the *Tnf* gene on chromosome 17, exon 4.
C Two guide RNA's were used to specifically delete the targeted region.

Data information: Figure created with Biorender.com.

 © 2023 The Authors

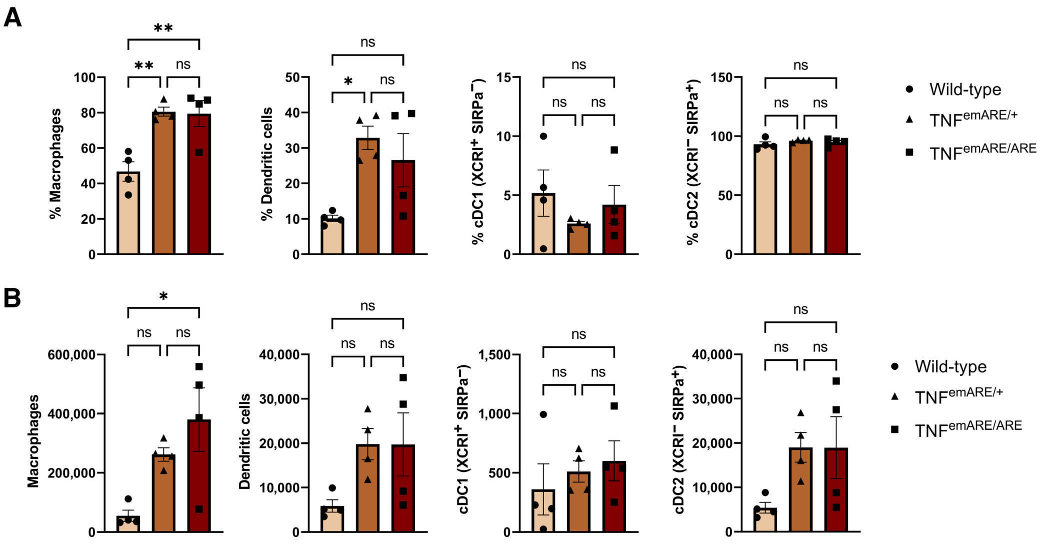

Figure EV2. Flow cytometry data of synovial leukocytes reveals a significant expansion of macrophages in TNF$^{emARE/+}$ and TNF$^{emARE/ARE}$ mice.

A Synovial flow cytometry data of 25 w/o TNF^{emARE} mice show an activated innate immune system (n = 8 mice/genotype, each datapoint represents data from two pooled mice).

B Absolute cell counts of synovial flow cytometry data (n = 8 mice/genotype, each datapoint represents data from two pooled mice).

Data information: Data are represented as Mean ± SEM, n = biological replicates with each datapoint on the graphs representing data of two pooled mice, one-way ANOVA test used with Tukey's multiple comparisons test. ns = P-value > 0.05, * = P-value ≤ 0.05, ** = P-value ≤ 0.01, *** = P-value ≤ 0.001, **** = P-value ≤ 0.0001.

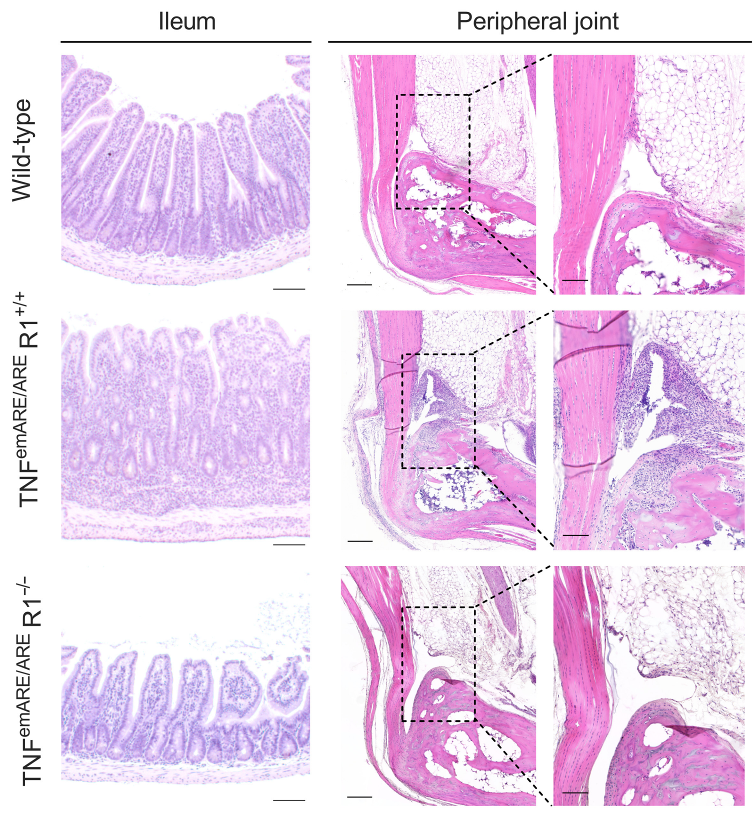

Figure EV3. TNF$^{emARE/ARE}$R1$^{-/-}$ mice are rescued from gut and joint pathology.

TNF$^{emARE/ARE}$R1$^{-/-}$ mice are rescued and do not display ileal pathology nor arthritis (Scale bars ileum: 100 µm, scale bars hind paws: 200 µm, scale bar focused images of SEC region: 100 µm).

© 2023 The Authors

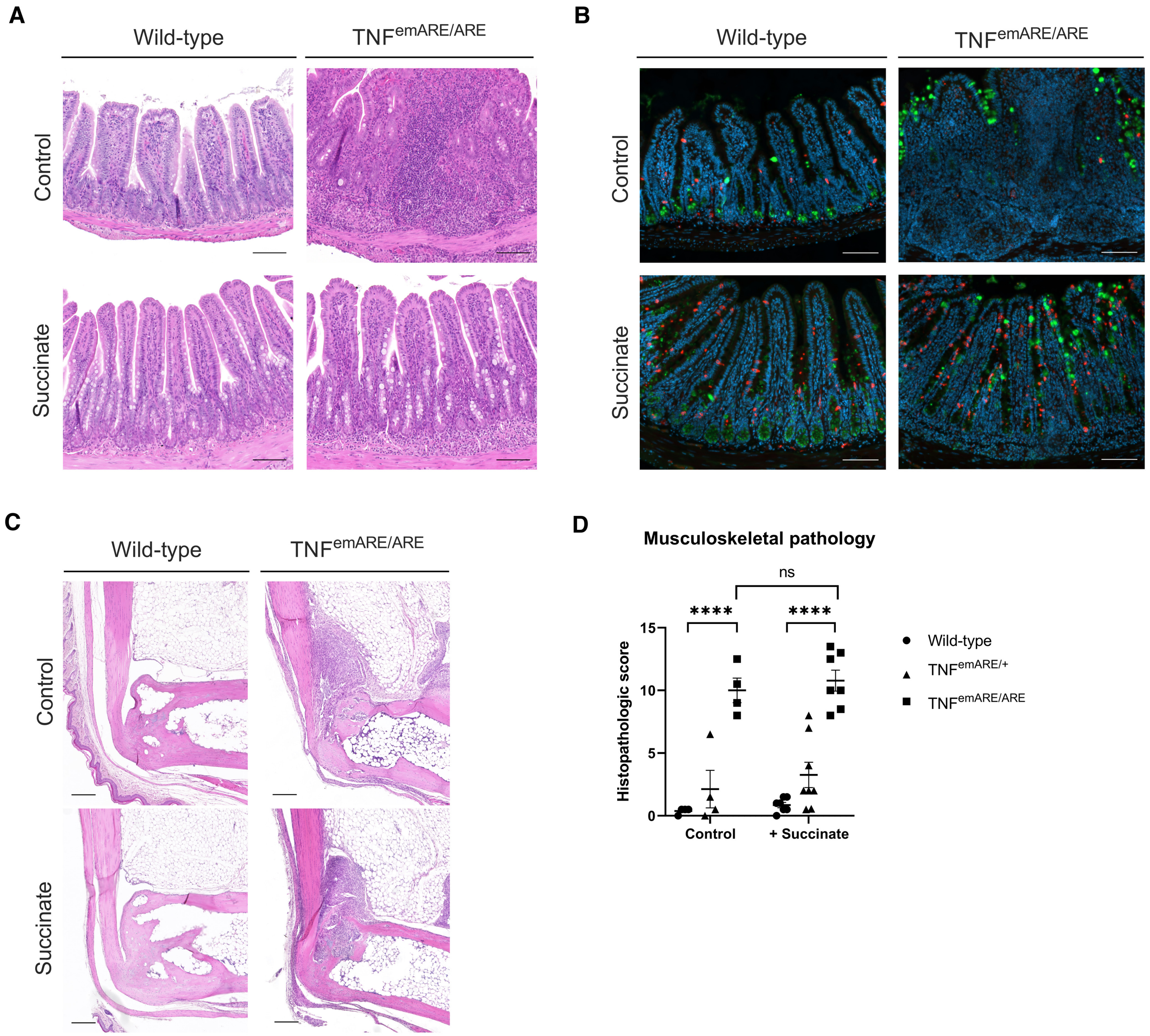

Figure EV4. Succinate supplementation induces tuft cell expansion and improvement of gut but not joint disease.

A Histological H&E sections of ileum indicate that succinate supplementation leads to improvement of ileal pathology (Scale bars: 100 μm).

B Immunofluorescent staining of DCLK1^{+} cells (red) shows expansion of tuft cells in succinate-treated animals, in both wild-types and TNF$^{emARE/ARE}$ mice. Mucins = green (WGA + UEA-1), nuclei = blue (Hoechst; Scale bars: 100 μm).

C Succinate-treated mice are not rescued from arthritis development, disease severity is similar to control mice (Scale bars: 200 μm).

D Quantitative analysis of musculoskeletal pathology confirms no amelioration of joint disease in mice that received succinate treatment (Control groups n = 4/genotype; succinate-treated mice n = 8/genotype).

Data information: For (D), data are represented as Mean ± SEM, n = biological replicates, two-way ANOVA test used with Tukey's multiple comparisons test. ns = P-value > 0.05, * = P-value ≤ 0.05, ** = P-value ≤ 0.01, *** = P-value ≤ 0.001, **** = P-value ≤ 0.0001.

 © 2023 The Authors

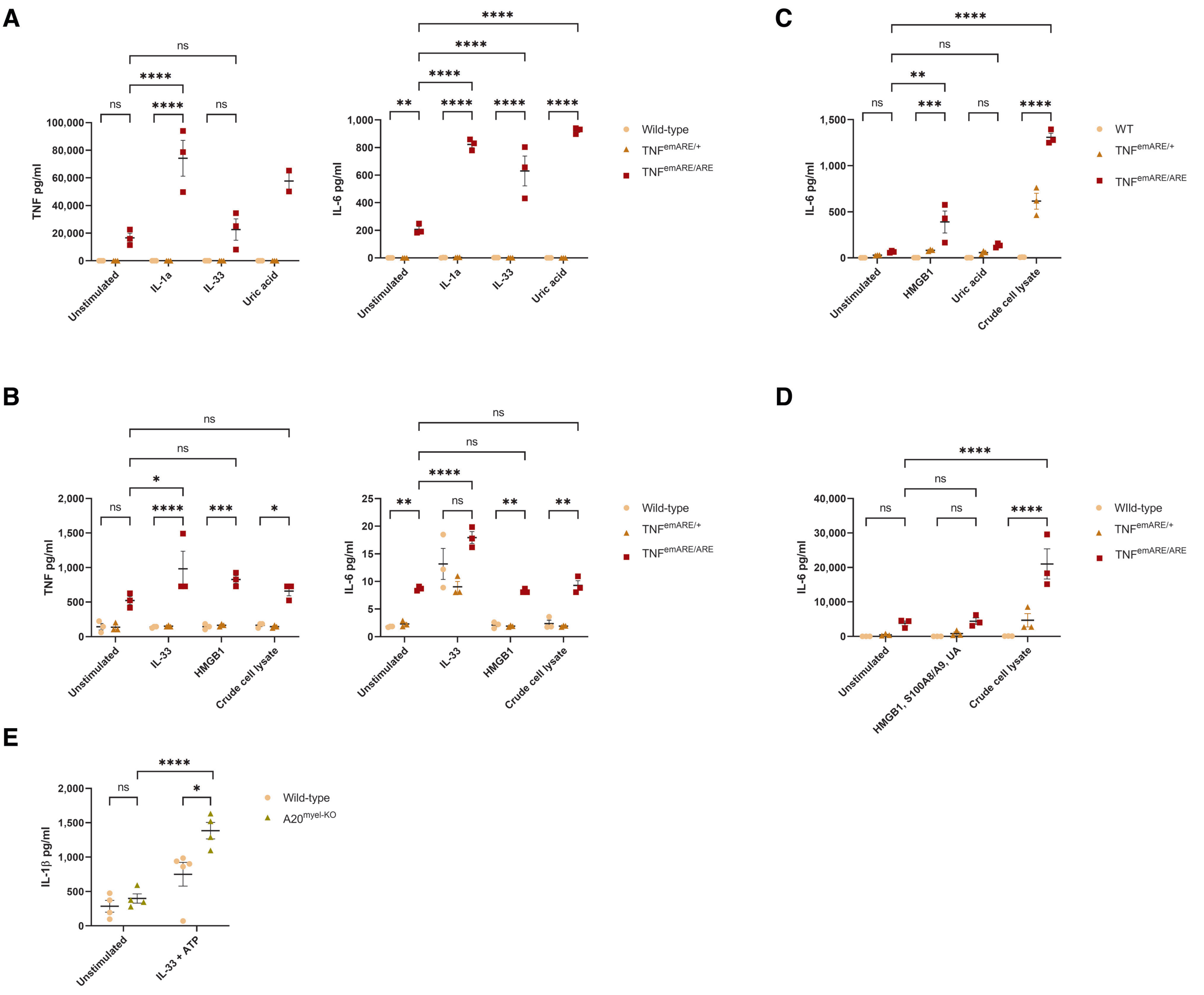

Figure EV5. BMDMs and SFs are hyperresponsive to stimulation with various DAMPs.

A Bio-plex cytokine assay for TNF and IL-6 on supernatant of BMDMs of 30–40 w/o $TNF^{emARE/ARE}$ mice after stimulation with various DAMPs (n = 3/genotype, except for uric acid stimulated $TNF^{emARE/ARE}$ cells n = 2).

B Bio-plex for TNF and IL-6 on supernatant of BMDMs of 7–9 w/o $TNF^{emARE/ARE}$ mice after stimulation with various DAMPs (n = 3/genotype).

C Bio-plex for IL-6 on supernatant of SFs of 30–40 w/o $TNF^{emARE/ARE}$ mice after stimulation with various DAMPs (n = 3/genotype).

D Bio-plex for IL-6 on supernatant of SFs of 7–9 w/o $TNF^{emARE/ARE}$ mice after stimulation with various DAMPs (n = 3/genotype).

E Bio-plex for IL-1beta on supernatant of BMDMs of 7–9 w/o $A20^{myel\text{-}KO}$ mice after stimulation with IL-33 + ATP (n = 4/genotype, except for IL-33 stimulated wild-type cells n = 5).

Data information: For (A, C), data are represented as Mean ± SEM, n = technical triplicates, two-way ANOVA test used with Tukey's multiple comparisons test. ns = P-value > 0.05, * = P-value ≤ 0.05, ** = P-value ≤ 0.01, *** = P-value ≤ 0.001, **** = P-value ≤ 0.0001. For (B, D, E), data are represented as Mean ± SEM, n = biological replicates, two-way ANOVA test used with Tukey's multiple comparisons test. ns = P-value > 0.05, * = P-value ≤ 0.05, ** = P-value ≤ 0.01, *** = P-value ≤ 0.001, **** = P-value ≤ 0.0001.

© 2023 The Authors
