# Supplementary material for: Sterile triggers drive joint inflammation in TNF‐ and IL‐1β‐dependent mouse arthritis models
Source: EMBO Mol Med. 2023 Sep 11;15(10):e17691. doi: 10.15252/emmm.202317691 (PMC10565626; doi:10.15252/emmm.202317691)
Supplement: Supplementary file 7 — PDF+ [file EMMM-15-e17691-s003.pdf]

Article

SOURCE DATA | TRANSPARENT PROCESS | OPEN ACCESS

EMBO Molecular Medicine

Sterile triggers drive joint inflammation in TNF- and IL-1β-dependent mouse arthritis models

Alexandra Thiran[1,2,3], Ioanna Petta[1,2,3], Gillian Blancke[1,2,3], Marie Thorp[1,2,3], Guillaume Planckaert[1,2], Maude Jans[1,2,3,4], Vanessa Andries[1,2,3], Korneel Barbry[2], Elisabeth Gilis[1,2], Julie Coudenys[1,2], Tino Hochepied[2,4], Christian Vanhove[5], Eric Gracey[1,2], Emilie Dumas[1,2], Teddy Manuelo[1,2], Ivan Josipovic[6], Geert van Loo[2,3,4], Dirk Elewaut[1,2,3,†] & Lars Vereecke[1,2,3,*,†]

Abstract

Arthritis is the most common extra-intestinal complication in inflammatory bowel disease (IBD). Conversely, arthritis patients are at risk for developing IBD and often display subclinical gut inflammation. These observations suggest a shared disease etiology, commonly termed "the gut-joint-axis." The clinical association between gut and joint inflammation is further supported by the success of common therapeutic strategies and microbiota dysbiosis in both conditions. Most data, however, support a correlative relationship between gut and joint inflammation, while causative evidence is lacking. Using two independent transgenic mouse arthritis models, either TNF- or IL-1β dependent, we demonstrate that arthritis develops independently of the microbiota and intestinal inflammation, since both lines develop full-blown articular inflammation under germ-free conditions. In contrast, TNF-driven gut inflammation is fully rescued in germ-free conditions, indicating that the microbiota is driving TNF-induced gut inflammation. Together, our study demonstrates that although common inflammatory pathways may drive both gut and joint inflammation, the molecular triggers initiating such pathways are distinct in these tissues.

Keywords arthritis; germ-free; gut-joint axis; intestinal inflammation; microbiome

Subject Categories Immunology; Microbiology, Virology & Host Pathogen Interaction; Musculoskeletal System

DOI 10.15252/emmm.202317691 | Received 9 March 2023 | Revised 21 August 2023 | Accepted 22 August 2023 | Published online 11 September 2023

EMBO Mol Med (2023) 15: e17691

Introduction

There is convincing evidence that gut and joint inflammation are clinically linked, particularly in spondyloarthritis (SpA), a group of inflammatory joint diseases which can affect both peripheral joints and the spine, and often present with extra-articular manifestations including ileitis, colitis, psoriasis, and uveitis (Taurog *et al*, 2016). Interestingly, acute infections with enteric pathogens like *Salmonella*, *Shigella*, and *Campylobacter* can trigger reactive arthritis (Taurog *et al*, 2016). 50% of all SpA patients present with subclinical gut inflammation, diagnosed by histological presence of microscopic gut inflammation, and 10% of all SpA patients eventually develop inflammatory bowel disease (IBD; Leirisalo-Repo *et al*, 1994; Mielants *et al*, 1995a, 1995b, 1995c; Van Praet *et al*, 2013; Kopylov *et al*, 2018). Furthermore, SpA emerges as the most prevalent extra-intestinal manifestation observed in IBD patients. The reported prevalence rates of SpA in individuals with IBD range between 6 and 46% (Brakenhoff *et al*, 2010; Ossum *et al*, 2018). Both axial and peripheral involvements have been reported, with peripheral SpA often coinciding with relapses of intestinal disease, while axial SpA appears to progress independently of the severity and activity of IBD (Sheth *et al*, 2015; Rogler *et al*, 2021; Barkhodari *et al*, 2022). Genome-wide association studies (GWAS) have revealed several shared disease susceptibility loci in IBD and SpA, including genes associated with innate immunity, type 3 immunity, and intestinal barrier integrity (Gracey *et al*, 2020). TNF is a pivotal pro-inflammatory cytokine and therapeutic target in multiple inflammatory conditions such as IBD and SpA, and the response rate to anti-TNF therapy is approximately 60–70%. Interestingly, in very early peripheral SpA, treatment with anti-TNF agents has shown a remarkable ability to induce sustained clinical drug-free remission, indicating the significance of an early intervention window that

1 Department of Internal Medicine and Pediatrics, Ghent University, Ghent, Belgium
2 VIB-UGent Center for Inflammation Research, Ghent, Belgium
3 Ghent Gut Inflammation Group (GGIG), Ghent, Belgium
4 Department of Biomedical Molecular Biology, Ghent University, Ghent, Belgium
5 Department of Electronics and Information Systems, Ghent University, Faculty of Engineering & Architecture, Ghent, Belgium
6 Department of Physics and Astronomy – Radiation Physics, Faculty of Science, RP-UGCT, Ghent University, Ghent, Belgium
*Corresponding author. Tel: +0032(0)93326404; E-mail: lars.vereecke@irc.vib-ugent.be
[†]These authors contributed equally to this work as senior authors

© 2023 The Authors. Published under the terms of the CC BY 4.0 license

maximizes the response to TNF inhibition (Carron *et al*, 2017). Despite its widespread use in clinical practice, TNF inhibition is associated with a notable proportion of non-responders and an increased risk of infection.

Various arthritic diseases, including rheumatoid arthritis (RA) and SpA, are characterized by shifts in intestinal microbial community structure and composition, and while this dysbiosis sometimes precedes arthritic disease onset, it remains unclear whether and to what extent it causally contributes to arthritis development (Breban *et al*, 2017; Ciccia *et al*, 2017; Tito *et al*, 2017; Zaiss *et al*, 2021). These observations have led to the dogma that joint inflammation is triggered or modulated by microbial signals and intestinal inflammation, and thus that intestinal pathology may precede and instigate joint inflammation. Various hypotheses have been suggested to support this idea, including the "arthritogenic peptide hypothesis" which suggests that microbial-derived antigens induce autoreactive T cells trough molecular mimicry, the "aberrant trafficking hypothesis" which claims that immune cells primed in the intestinal mucosa home to synovial tissues and cause inflammation, and the "dysbiosis hypothesis" which states that a shift in microbiota composition drives both intestinal and joint inflammation through various mechanisms (Qaiyum *et al*, 2021). Despite the strong correlation of gut and joint inflammation in SpA, there is no consensus that inflammation in the joint depends on intestinal dysbiosis or inflammatory events in the gut. Shared inflammatory pathways may underlie both gut and joint inflammation, but can be triggered by independent and tissue-specific factors, which can either be microbial-derived or sterile triggers. Previous studies have demonstrated that TNF-driven intestinal inflammation in $TNF^{\Delta ARE}$ mice (Kontoyiannis *et al*, 1999) is microbiota-dependent, as $TNF^{\Delta ARE}$ mice only develop spontaneous ileitis in colonized conditions, but not when raised under germ-free (GF) conditions (Roulis *et al*, 2016; Schaubeck *et al*, 2016). Despite the clear protection from intestinal inflammation, it is not clear whether GF $TNF^{\Delta ARE}$ mice are equally protected from spontaneous joint inflammation. In order to investigate the gut-joint axis in detail, we studied two transgenic mouse models of arthritis, one which depends on the cytokine TNF, and one which is IL-1β-driven, and evaluated the development of gut and joint inflammation in both colonized (specific pathogen free, SPF) and in GF conditions.

Results

Development and characterization of a new TNF-driven transgenic mouse model

Given the importance of TNF in various human inflammatory diseases, including IBD and SpA, we generated a new TNF-driven mouse inflammation model by targeting the AU-Rich element (ARE) of the *Tnf* gene using a double guide-RNA-mediated CRISPR/CAS9 approach, resulting in a 107-bp deletion in the 3′ UTR of the *Tnf* gene on Chromosome 17 (Fig EV1). This deletion is predicted to generate a more stable *Tnf* mRNA compared to wild-type *Tnf* RNA, since binding of the ARE sequence and subsequent mRNA decay by TIS11 family RNA-binding proteins is prevented. The more stable mRNA results in elevated levels of bioactive TNF protein upon translation (Kontoyiannis *et al*, 1999; Makita *et al*, 2021). This new TNF overexpressing mouse line, C57Bl6/J-$Tnf^{emARE1Irc}$ (endonuclease modified, from now on termed TNF^{emARE}), was generated under SPF conditions and later rederived in GF conditions in the GF and gnotobiotic mouse facility at Ghent University. Macroscopically, both homozygous $TNF^{emARE/ARE}$ and heterozygous $TNF^{emARE/+}$ mice showed stunted growth, in contrast to wild-type littermates (Fig 1A and B). Serum TNF levels were slightly elevated in heterozygous $TNF^{emARE/+}$ mice, and significantly higher in homozygous $TNF^{emARE/ARE}$ mice compared to wild-type littermates, confirming TNF overexpression in this model (Fig 1C).

Multiple organs from wild-type, heterozygous $TNF^{emARE/+}$, and homozygous $TNF^{emARE/ARE}$ mice were isolated and evaluated for signs of inflammation. Hematoxylin-eosin (H&E) stained sections of lung, liver, kidney, skin, and colon showed no abnormalities in all three genotypes (Appendix Fig S1A). However, sections of small intestine and joints revealed strong inflammation in $TNF^{emARE/ARE}$ mice, but only minimal inflammation in $TNF^{emARE/+}$ mice,

Figure 1. SPF TNF^{emARE} mice suffer from inflammatory gut and joint disease.

A Macroscopic picture of a male wild-type, male $TNF^{emARE/+}$, and male $TNF^{emARE/ARE}$ mouse showing stunted growth in the hetero- and homozygous conditions. Mice were 27–30 w/o.
B Changes in body weight of wild-type (n = 8; F = 3, M = 5), $TNF^{emARE/+}$ (n = 8; F = 4, M = 4), and $TNF^{emARE/ARE}$ (n = 9; F = 6, M = 3) mice over time for both male and female population. SPF $TNF^{emARE/ARE}$ and $TNF^{emARE/+}$ mice are smaller compared to wild-type littermates, which is reflected in their smaller body weight and limited body weight increase.
C $TNF^{emARE/ARE}$ mice show significantly elevated serum levels of TNF (wild-type n = 5, $TNF^{emARE/+}$ n = 5, $TNF^{emARE/ARE}$ n = 5).
D Histologic H&E sections of ileum and hind paw indicate severe ileitis and arthritis in 20–30 w/o SPF $TNF^{emARE/ARE}$ mice (Scale bars upper panel: 100 µm, scale bars lower panel: 500 µm).
E Quantification of ileitis in wild-type (n = 8), $TNF^{emARE/+}$ (n = 7) and $TNF^{emARE/ARE}$ (n = 5) mice of 10–20 w/o.
F Quantification of musculoskeletal inflammation in hind paws of wild-type (n = 7), $TNF^{emARE/+}$ (n = 9) and $TNF^{emARE/ARE}$ (n = 5) mice of 10–20 w/o.
G tSNE analysis performed on $CD45^+$ lineage (excluding $CD3^+$, $CD19^+$, $NK1.1^+$ fractions) of lamina propria of ileum samples (n = 5/genotype).
H Ileal flow cytometry data of 25 w/o TNF^{emARE} mice show a highly activated innate immune system (n = 5/genotype).
I tSNE analysis performed on $CD3^+$ cells of lamina propria of ileum samples (n = 5/genotype).
J Ileal flow cytometry data show an increase of $CD4^+$ cells in transgenic mice, which can be designated to the enrichment of the $CD4^+RORgt^+$ (Th17) cell population (n = 5/genotype).

Data information: In (B, C, E, F, H, J), data are represented as Mean ± SEM, n = biological replicates. For (B), statistics are explained in the Materials and Methods section, for (C, E, F, H, J), one-way ANOVA test was used with Tukey's multiple comparisons test, ns = P-value > 0.05, * = P-value ≤ 0.05, ** = P-value ≤ 0.01, *** = P-value ≤ 0.001, **** = P-value ≤ 0.0001.
Source data are available online for this figure.

© 2023 The Authors

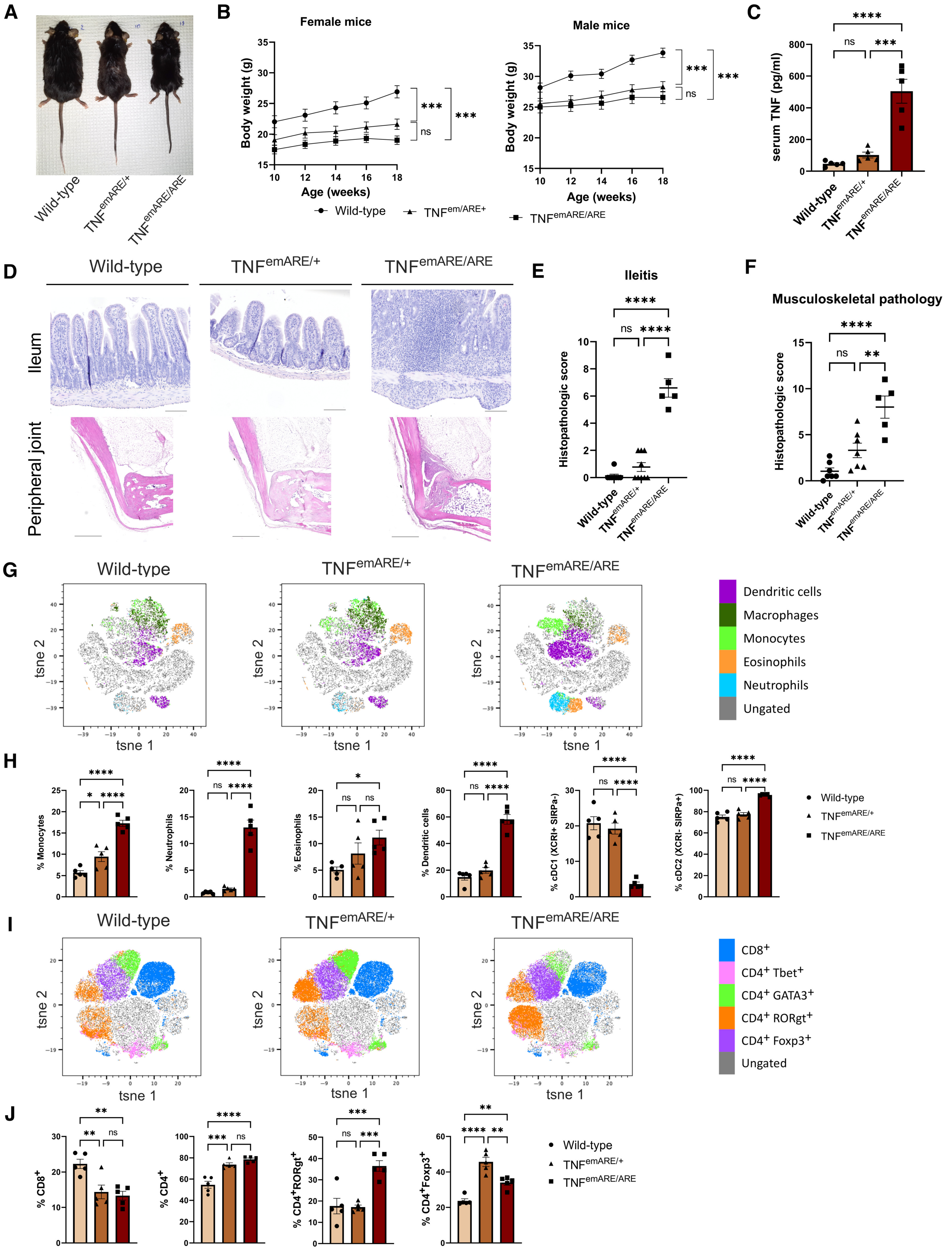
A
Wild-type
TNFemARE/+
TNFemARE/ARE
B
Female mice
Male mice
Body weight (g)
Age (weeks)
Wild-type
TNFem/ARE+
TNFemARE/ARE
C
serum TNF (pg/ml)
D
Ileum
Peripheral joint
E
Ileitis
Histopathologic score
F
Musculoskeletal pathology
G
Dendritic cells
Macrophages
Monocytes
Eosinophils
Neutrophils
Ungated
tsne 1
tsne 2
H
% Monocytes
% Neutrophils
% Eosinophils
% Dendritic cells
% cDC1 (XCRI+ SIRPa-)
% cDC2 (XCRI- SIRPa+)
I
CD8+
CD4+ Tbet+
CD4+ GATA3+
CD4+ RORgt+
CD4+ Foxp3+
J
% CD8+
% CD4+
% CD4+RORgt+
% CD4+Foxp3+

Figure 1.

compared to wild-type littermate controls that showed no inflammation (Fig 1D and Appendix Fig S1A). We assessed *Tnf* expression in multiple tissues of wild-type, heterozygous $TNF^{emARE/+}$, and homozygous $TNF^{emARE/ARE}$ mice by RT-QPCR and detected elevated *Tnf* in $TNF^{emARE/ARE}$ colon, ileum, kidney, spine, and knee synovium, while *Tnf* expression was not significantly upregulated in liver, lung, skin, and spleen (Appendix Fig S1B). Ileal pathology in $TNF^{emARE/ARE}$ mice is characterized by massive immune cell infiltration, loss of goblet cells, and villus atrophy (Fig 1D and E), which is most severe in ileum, and rather modest in the jejunum and duodenum (Appendix Fig S2). Peripheral musculoskeletal pathology was assessed by histological analysis of H&E-stained ankle sections, demonstrating severe inflammation in $TNF^{emARE/ARE}$ mice, characterized by immune cell infiltration along the Achilles tendon, in the synovio-entheseal complex (SEC) and Kargers' fat pad and in the calcaneus (Fig 1D). The calcaneus showed bone erosion and mild bone marrow edema. In contrast, $TNF^{emARE/+}$ mice developed mild to no obvious signs of joint inflammation. Histopathological scoring based on immune cell infiltration, bone remodeling, and bone marrow edema in the calcaneocuboid joint, the calcaneus and the SEC indicated strong arthritis development in $TNF^{emARE/ARE}$ mice (Fig 1F). Both ileal and musculoskeletal pathology have an early onset, as we observed first signs of gut and joint inflammation from the age of 5–6 weeks in $TNF^{emARE/ARE}$ mice (Appendix Fig S3). Quantification of ileal lamina propria leukocytes by flow cytometry showed strongly expanded populations of monocytes, eosinophils, neutrophils, and dendritic cells (DC) in homozygous $TNF^{emARE/ARE}$ mice (Fig 1G and H, and Appendix Fig S4). Increased dendritic cell populations were primarily cDC2s ($XCRI^-SIRPa^+$), as the cDC1 ($XCRI^+SIRPa^-$) population was drastically reduced in $TNF^{emARE/ARE}$ mice. We also observed changes in the T cell compartment, including increased $CD4^+$ T cell numbers and particularly $CD4^+$ RORγt cells (Fig 1I and J). In contrast, in heterozygous $TNF^{emARE/+}$ mice, ileal inflammation, and myeloid and T cell expansion was minimal. To investigate whether similar immune cell profiles could be observed in inflamed joints, synovial leukocytes were quantified by flow cytometry using the same myeloid panel and gating strategy (Fig EV2). For both hetero- and homozygous mice, the population of macrophages was significantly increased and a trend toward an expansion of DCs could be observed. We further confirmed that the inflammatory phenotype of $TNF^{emARE/ARE}$ mice is dependent on TNF, since $TNF^{emARE/ARE}$ mice were fully protected from spontaneous gut and joint inflammation when backcrossed in a TNF receptor 1 (TNFR1 or p55)-deficient background (Fig EV3).

In conclusion, this new TNF^{emARE} mouse line, which is characterized by TNFR1-mediated spontaneous Crohn's-like ileitis and musculoskeletal pathology development, resembles previously generated $TNF^{\Delta ARE}$ mice (Kontoyiannis *et al*, 1999). In contrast to $TNF^{\Delta ARE}$ mice, TNF^{emARE} mice only develop severe inflammatory pathology in homozygous conditions ($TNF^{emARE/ARE}$ mice), while heterozygous $TNF^{emARE/+}$ mice display minimal to no inflammation in gut and joints. We used this new TNF^{emARE} line to study microbiota dependency for the development of intestinal and joint inflammation.

The microbiota instigates TNF-driven ileitis but not arthritis

Previous studies have shown that $TNF^{\Delta ARE}$ mice are characterized by microbial dysbiosis, and that intestinal inflammation is microbiota-dependent (Roulis *et al*, 2016; Schaubeck *et al*, 2016). To investigate the contribution of the microbiota to TNF-driven inflammatory pathology in both gut and joints, TNF^{emARE} mice were rederived in germ-free (GF) conditions by axenic embryo transfer in the GF and gnotobiotic mouse facility at Ghent University. In contrast to SPF-raised mice, GF-raised $TNF^{emARE/ARE}$ and $TNF^{emARE/+}$ mice had similar body weight as their wild-type littermates (Fig 2A and B). Both SPF and GF $TNF^{emARE/ARE}$ mice had significantly elevated TNF serum levels compared to wild-type littermate mice (Fig 2C). Ileal *Tnf* expression was significantly increased in SPF-raised, as well as in GF-raised $TNF^{emARE/ARE}$ mice (Fig 2D). However, expression of *Tnf* in the ileum of homozygote GF mice was lower compared to *Tnf* expression in their SPF counterpart. As TNF-mediated Paneth cell depletion was previously reported (Roulis *et al*, 2016), we performed immunostaining of Paneth cells by anti-lysozyme staining on ileal sections and observed complete Paneth cell depletion in $TNF^{emARE/ARE}$ mice raised in SPF but not under GF conditions (Fig 2E). Ileal inflammation was further evaluated by H&E staining, and unlike SPF $TNF^{emARE/ARE}$ mice which developed

Figure 2. GF TNF^{emARE} mice are rescued from gut pathology.

A Macroscopic picture of a GF male wild-type, male $TNF^{emARE/+}$, and male $TNF^{emARE/ARE}$ mouse shows no large differences in posture, except for a shorter tail of the homozygote. Mice were between 20 and 24 w/o.

B There is a trend in reduced body weight in 20–30 w/o SPF mice, which is less clear in the age-matched GF mice (SPF wild-type $nF = 6$, $nM = 11$; SPF $TNF^{emARE/+}$ $nF = 12$, $nM = 9$; SPF $TNF^{emARE/ARE}$ $nF = 16$, $nM = 9$; GF wild-type $nF = 5$, $nM = 5$; GF $TNF^{emARE/+}$ $nF = 2$, $nM = 8$; GF $TNF^{emARE/ARE}$ $nF = 3$, $nM = 6$).

C Serum levels of TNF are elevated in hetero- and homozygous transgenic mice, in both SPF and GF conditions ($n = 5$ mice/group, except for GF wild-type $n = 4$).

D Quantitative real-time PCR reveals increased expression of the *Tnf* gene in the ileum in homozygous SPF and GF mice (SPF wild-type $n = 10$, SPF $TNF^{emARE/+}$ $n = 10$, SPF $TNF^{emARE/ARE}$ $n = 13$; GF wild-type $n = 3$, GF $TNF^{emARE/+}$ $n = 4$, GF $TNF^{emARE/ARE}$ $n = 4$).

E Immunofluorescent ileal sections indicate Paneth cell depletion in SPF $TNF^{emARE/ARE}$ mice, while these cells are still detected on ileal sections of GF $TNF^{emARE/ARE}$ mice. Paneth cells = red (anti-lysozyme), mucins = green (WGA + UEA-1), nuclei = blue (Hoechst) (Scale bars: 20 μm).

F Comparing SPF versus GF H&E sections of the ileum shows complete rescue of gut inflammation under axenic conditions in 20–30 w/o $TNF^{emARE/ARE}$ mice (Scale bars: 100 μm).

G Histopathological scoring quantitatively confirms rescue of ileal disease when mice are housed GF. Samples of 20–30 w/o mice were scored blindly (SPF wild-type $n = 6$, SPF $TNF^{emARE/+}$ $n = 6$, SPF $TNF^{emARE/ARE}$ $n = 9$; GF wild-type $n = 7$, GF $TNF^{emARE/+}$ $n = 6$, GF $TNF^{emARE/ARE}$ $n = 8$).

Data information: For graph (B), violin plots represent the 25- and 75- percentile with median values as central band, n = biological replicates, two-way ANOVA test used with Tukey's multiple comparisons test, ns = P-value > 0.05, * = P-value ≤ 0.05, ** = P-value ≤ 0.01, *** = P-value ≤ 0.001, **** = P-value ≤ 0.0001. For panels (C, D and G) data are represented as Mean ± SEM, n = biological replicates, two-way ANOVA test used with Tukey's multiple comparisons test, ns = P-value > 0.05, * = P-value ≤ 0.05, ** = P-value ≤ 0.01, *** = P-value ≤ 0.001, **** = P-value ≤ 0.0001.
Source data are available online for this figure.

 © 2023 The Authors

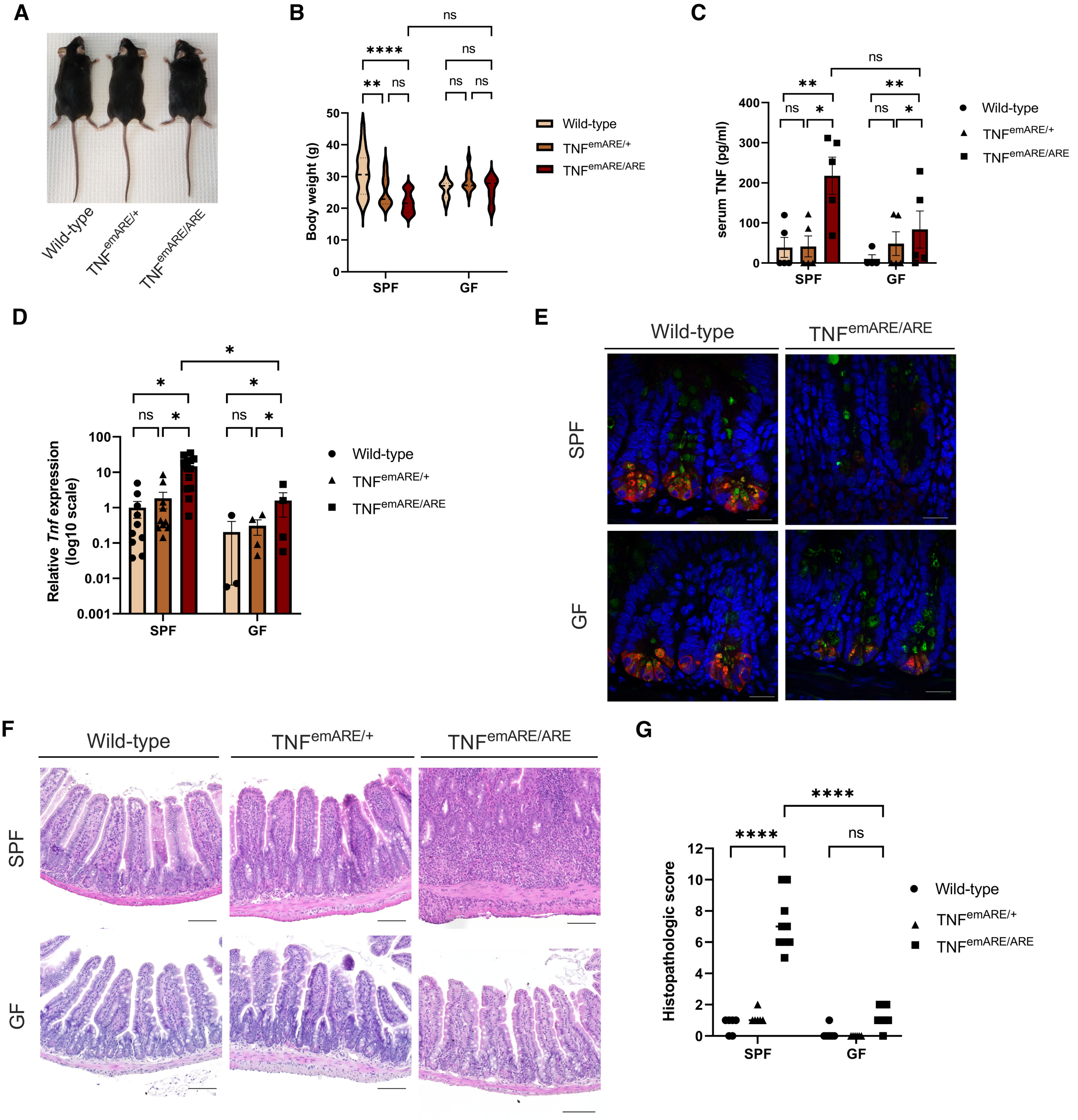

Figure 2.

severe ileitis in SPF conditions, GF TNF$^{emARE/ARE}$ mice were completely protected from ileitis (Fig 2F), which was confirmed by pathophysiological scoring of epithelial damage, structural changes, and immune cell infiltration (Fig 2G). Together, these data confirm previous findings in TNF$^{\Delta ARE}$ mice (Roulis *et al*, 2016; Schaubeck *et al*, 2016) and indicate that TNF-driven intestinal inflammation is fully dependent on the intestinal microbiota.

Live *in vivo* imaging using fluorodeoxyglucose (FDG)-based PET-CT analysis was performed on SPF and GF raised wild-type and TNF$^{emARE/ARE}$ mice, to visualize structural elements and sites of active inflammation in the whole body. PET-CT scans revealed multi-articular inflammation in front and hind paws, spinal column inflammation and non-congenital deformation (hyperkyphosis), in both SPF and GF raised TNF$^{emARE/ARE}$ mice (Fig 3A). SPF TNF$^{emARE/ARE}$ mice displayed elevated FDG signal in the abdominal area, indicating active intestinal inflammation. In contrast, no FDG signal was observed in the abdominal area of GF-raised TNF$^{emARE/ARE}$ mice (Fig 3A). We next performed H&E staining on histological

© 2023 The Authors

sections of the upper axial skeleton and observed immune cell infiltration from the cervical to the thoracic part, mainly along the spinal longitudinal ligament and in the intervertebral discs of both SPF and axenic TNF$^{emARE/ARE}$ mice (Fig 3B). To evaluate peripheral disease, sections of hind paws were stained with H&E (Fig 3C). Severe musculoskeletal pathology was observed in both SPF and GF raised TNF$^{emARE/ARE}$ mice, characterized by strong immune cell infiltration, bone erosion, and bone marrow edema. Musculoskeletal disease was scored for the axial component and peripheral component and confirmed a pathologic signature in both SPF and GF TNF^{e-}mARE/ARE mice in axial and peripheral joints (Fig 3D). In contrast to TNFemARE/ARE mice, heterozygous TNF$^{emARE/+}$ mice did not display inflammatory infiltrates in the joints or spinal deformations in either housing condition, in line with previous observations. To better characterize axial and peripheral pathology, µCT analysis was performed to visualize bone erosions in the calcaneus, and spondylosis and ankylosis of the vertebrae in detail. This high-resolution imaging technique is the preferred approach to study destructive effects of inflammation on the skeleton. Structural deformations (bone erosions) of the posterior calcaneus at the level of synovio-entheseal complex were clearly detectable, in both SPF and GF TNF$^{emARE/ARE}$ mice (Fig 3E). Moreover, caudal vertebrae of the tail displayed vertebral fusion (Fig 3F).

To study the functional joint disability in TNF$^{emARE/ARE}$ mice, we measured their grip strength compared to heterozygous TNF$^{emARE/+}$ and wild-type littermates, both in mice raised in SPF and GF conditions. Both colonized and axenic TNF$^{emARE/ARE}$ mice had strongly reduced grip strength compared to wild-type controls (Fig 4A). Moreover, moving patterns of SPF and GF mice were studied using the CatWalk XT technology (Noldus). This technique allows to study mouse gait and locomotion in detail by capturing footprints as the mouse is traversing a glass walkway (representative videos in Movies EV1–EV4). Both SPF and GF TNF$^{emARE/ARE}$ mice were characterized by aberrant gait, as they displayed smaller footprints, had lower foot surface pressure, and had reduced stride length compared to wild-type and TNF$^{emARE/+}$ littermates (Fig 4B). Via illuminated footprint technology, each part of the paw that is in contact with the walkway can be detected and visualized. Footprints of both TNF$^{emARE/ARE}$ SPF and GF mice showed poorly defined foot contours (Fig 4C). These functional assays indicate reduced grip strength and abnormal gait as a result of severe arthritis in both SPF- and GF-raised TNF$^{emARE/ARE}$ mice.

Microbiota-derived succinate was previously shown to suppress ileal inflammation in TNF$^{\Delta ARE}$ mice, through tuft cell activation and expansion, while no data were shown on possible effects on joint inflammation (Banerjee *et al*, 2020). We investigated the gut-joint axis, and the assumption that joint inflammation is influenced by the intensity of intestinal pathology, by evaluating whether succinate supplementation via the drinking water (ad libitum) to SPF TNF$^{emARE/ARE}$ mice improves not only gut but possibly also joint disease. We found succinate to promote tuft cell expansion and suppress ileal inflammation in TNF$^{emARE/ARE}$ mice (Fig EV4). In contrast, succinate administration had no impact on musculoskeletal inflammation, as shown on histological sections of ankle joints and supportive quantitative scoring of disease severity. These data show that improving gut inflammation does not necessarily improve joint inflammation, and rather support a functional disconnection of gut and joint pathology.

Together, these data clearly indicate that intestinal TNF-driven pathology in TNF$^{emARE/ARE}$ mice is dependent on the intestinal microbiota, as GF TNF$^{emARE/ARE}$ mice are fully protected from spontaneous ileitis development. In contrast, the microbiota is dispensable for TNF-driven musculoskeletal pathology in TNF$^{emARE/ARE}$ mice, as GF TNF$^{emARE/ARE}$ mice still develop severe arthritis. These data clearly indicate a functional disconnection of gut and musculoskeletal pathophysiology in this TNF-driven mouse model.

Joint inflammation in A20$^{myel\text{-}KO}$ mice is driven by sterile triggers

In addition to a TNF-driven arthritis model, we also evaluated the importance of the microbiome for arthritis development in the IL-1β-dependent myeloid-specific A20-deficient (A20$^{myel\text{-}KO}$) mouse model of arthritis (Matmati *et al*, 2011; Walle *et al*, 2014). Inflammasome activation has been observed in both preclinical models and in patients with SpA, which was shown to drive type-3 cytokine production in an IL-1β-dependent mechanism, and found to be

Figure 3. TNF$^{emARE/ARE}$ mice display axial and peripheral arthritis in both SPF and GF conditions. ▶

A Wild-type and TNF$^{emARE/ARE}$ mice housed in either SPF and/or GF conditions were subjected to PET-CT live *in vivo* imaging analysis. SPF TNF$^{emARE/ARE}$ mice only show high uptake of FDG at the level of the intestines (indicated by a white asterisk), while SPF and GF TNF$^{emARE/ARE}$ show inflammation at axial and peripheral joints and kyphosis of the spine. Regions in the mouse body showing high FDG uptake other than gut or joints are mainly artifacts because of tail vein injection, bladder content, physiologic myocardial uptake of FDG or active brown fat.

B Thoracic spinal histological H&E sections of an SPF wild-type versus SPF TNF$^{emARE/+}$ and TNF$^{emARE/ARE}$ and GF TNF$^{emARE/ARE}$ mouse reveal immune cells infiltrating along the spinal longitudinal ligament (indicated by black arrows; Scale bars: 100 µm).

C 20–30 w/o SPF TNF$^{emARE/ARE}$ mice clearly suffer from inflammation in the synovio-entheseal complex, which is not rescued in age-matched GF mice (Scale bars: 200 µm).

D Histopathological scoring indicates a significant increase of immune cells infiltrating along the spinal longitudinal ligament (SPF wild-type $n = 5$, SPF TNF$^{emARE/+}$ $n = 5$, SPF TNF$^{emARE/ARE}$ $n = 8$; GF wild-type $n = 4$, GF TNF$^{emARE/+}$ $n = 3$, GF TNF$^{emARE/ARE}$ $n = 6$) and severe peripheral arthritis development (SPF wild-type $n = 6$, SPF TNF$^{emARE/+}$ $n = 7$, SPF TNF$^{emARE/ARE}$ $n = 9$; GF wild-type $n = 5$, GF TNF$^{emARE/+}$ $n = 5$, GF TNF$^{emARE/ARE}$ $n = 7$) in both SPF and GF TNF$^{emARE/ARE}$ mice.

E µCT images of wild-type versus SPF and GF TNF$^{emARE/ARE}$ mouse show structural deformations (bone erosions) of the posterior calcaneus in homozygous mice as a result of inflammation.

F µCT images of tails of wild-type versus SPF and GF TNF$^{emARE/ARE}$ mouse display fusion of sacral vertebrae in homozygous SPF and GF mice, as indicated by yellow arrows.

Data information: All mice used for these experiments were between 20 and 40 weeks old. For graph (D), data are represented as Mean ± SEM, n = biological replicates, two-way ANOVA test used with Tukey's multiple comparisons test, ns = P-value > 0.05, * = P-value ≤ 0.05, ** = P-value ≤ 0.01, *** = P-value ≤ 0.001, **** = P-value ≤ 0.0001.
Source data are available online for this figure.

© 2023 The Authors

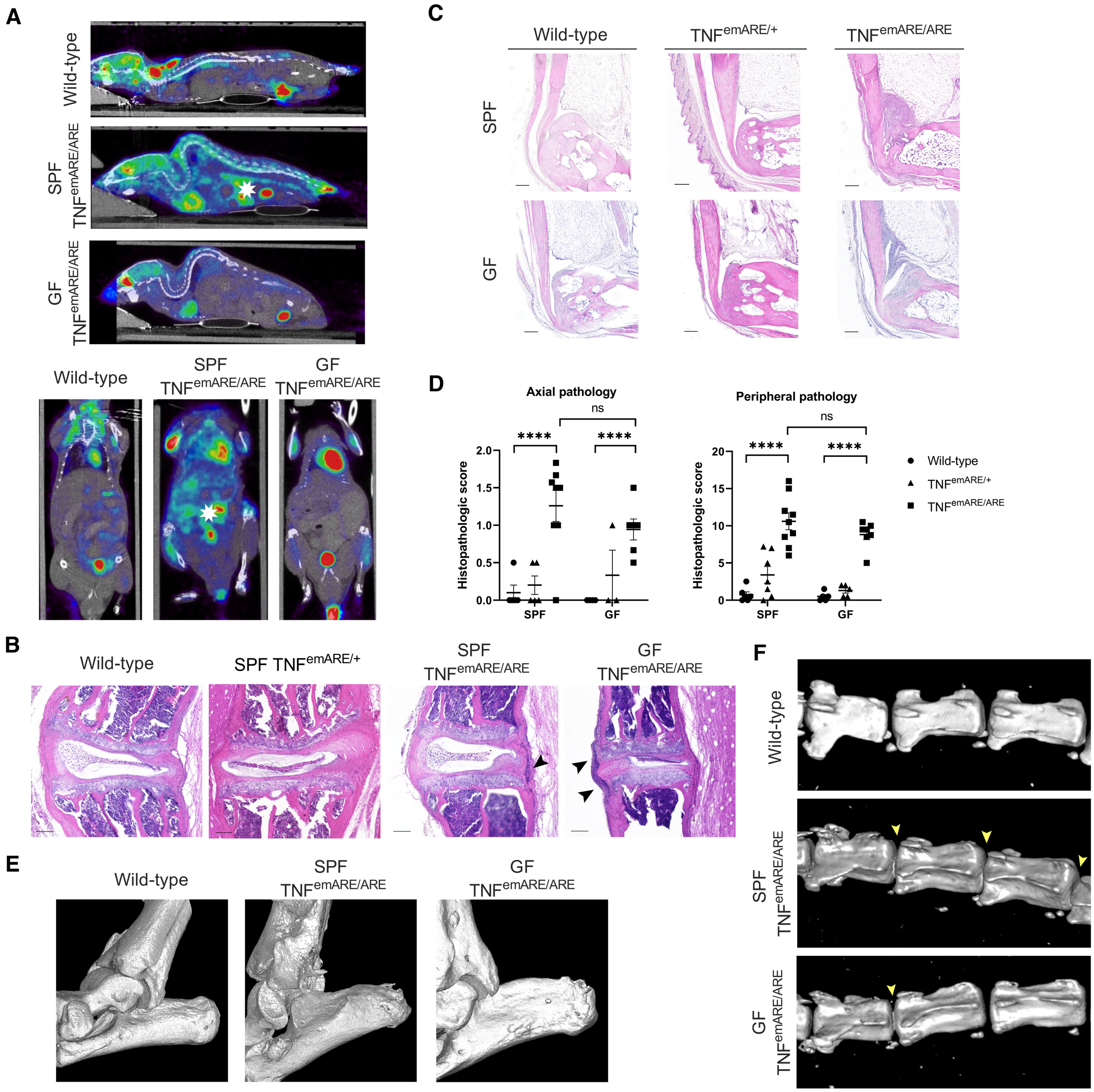

Figure 3.

associated with intestinal dysbiosis (Guggino *et al*, 2021). Anakinra, a human interleukin-1 receptor antagonist, is currently used to treat patients suffering from various inflammasomopathies, including crystal-induced arthropathies such as gout (Malcova *et al*, 2021). A20$^{myel\text{-}KO}$ mice, generated by crossing floxed-*A20/Tnfaip3* mice with Lysozyme-Cre transgenic mice, which leads to conditional *A20* deletion in the myeloid compartment, were previously shown to develop an erosive TNF-independent but NLRP3 inflammasome- and IL-1β-dependent polyarthritis resembling rheumatoid arthritis. In this model, arthritis develops as a result of macrophage necroptosis, which leads to inflammasome activation and the release of IL-1β and intracellular danger-associated molecular patterns (DAMPs; Matmati *et al*, 2011; Walle *et al*, 2014; Polykratis *et al*, 2019). A20$^{myel\text{-}KO}$ mice develop RA-like pathology but do not develop intestinal pathology in the small and large intestines; however, they are characterized by microbial dysbiosis (Matmati *et al*, 2011; Vereecke *et al*, 2014; Walle *et al*, 2014). IL-1β is known to play an important role in rheumatic diseases, but the upstream mechanisms leading to production of this interleukin are still incompletely understood (Lori Broderick, 2022). A20 negatively regulates inflammatory responses initiated by multiple pattern-recognition receptors and cytokine receptors. Moreover, polymorphisms in the *A20/Tnfaip3* locus are

© 2023 The Authors

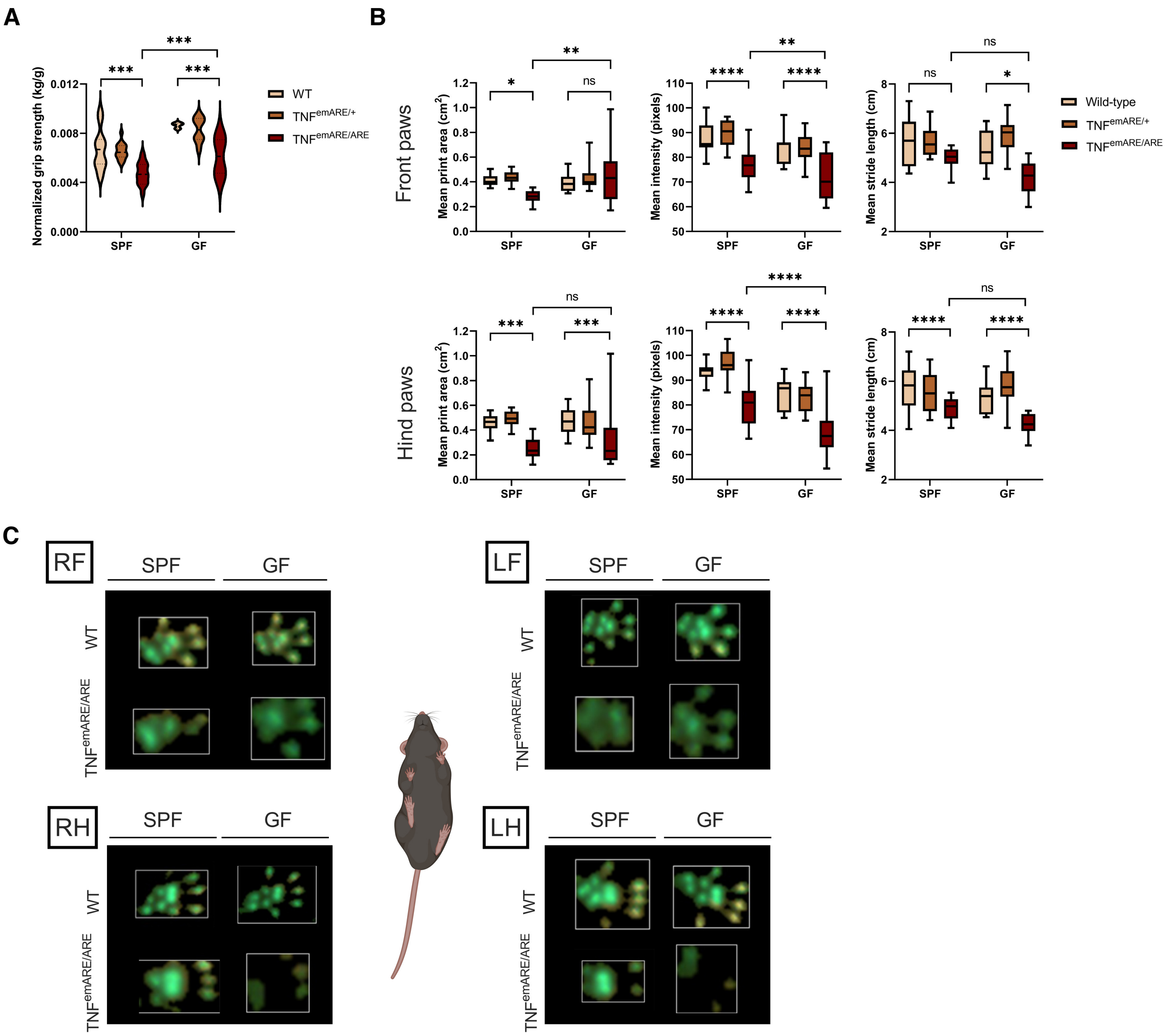

Figure 4. Gait analysis of TNF^{emARE} mice using the Noldus CatWalk gait analysis system.

A Grip strength analysis of four paws of 15–30 w/o TNF^{emARE} mice shows a significant reduction in strength in TNF$^{emARE/ARE}$ mice compared to their wild-type littermates, in both SPF and GF conditions. (SPF wild-type $nF = 4$, $nM = 4$; SPF TNF$^{emARE/+}$ $nF = 4$, $nM = 4$; SPF TNF$^{emARE/ARE}$ $nF = 4$, $nM = 4$; GF wild-type $nF = 4$, $nM = 3$; GF TNF$^{emARE/+}$ $nF = 3$, $nM = 4$; GF TNF$^{emARE/ARE}$ $nF = 3$, $nM = 4$).

B Gait analysis of TNF^{emARE} mice reveals distinct moving patterns of SPF and GF TNF$^{emARE/ARE}$ mice. Homozygous mice tend to have smaller footprints, place their feet with lower mean intensity and generally have a smaller stride length. (SPF wild-type $n = 12$, SPF TNF$^{emARE/+}$ $n = 12$, SPF TNF$^{emARE/ARE}$ $n = 12$; GF wild-type $n = 8$, GF TNF$^{emARE/+}$ $n = 20$, GF TNF$^{emARE/ARE}$ $n = 12$).

C Representative wild-type and TNF$^{emARE/ARE}$ mouse footprints of mice raised under SPF versus GF conditions. Footprints of wild-type mice show precise prints visualizing the whole sole and toes, while in TNF$^{emARE/ARE}$ mice the footprints are not clearly defined and prints of digits are missing. (RF = right front, LF = left front, RH = right hind, LH = left hind).

Data information: Mice used for this analysis were between 20 and 40 weeks old. For graph (A), violin plots represent the 25 and 75 percentile with median values as central band, n = biological replicates, two-way ANOVA test used with Tukey's multiple comparisons test, ns = P-value > 0.05, * = P-value ≤ 0.05, ** = P-value ≤ 0.01, *** = P-value ≤ 0.001, **** = P-value ≤ 0.0001. For panel (B), boxplots represent the 25 and 75 percentile with median values as central band, whiskers span min to max value range, n = biological replicates with every datapoint representing one paw, two-way ANOVA test used with Tukey's multiple comparisons test, ns = P-value > 0.05, * = P-value ≤ 0.05, ** = P-value ≤ 0.01, *** = P-value ≤ 0.001, **** = P-value ≤ 0.0001.
Source data are available online for this figure.

 © 2023 The Authors

associated with many inflammatory and autoimmune diseases, including IBD, SLE, and arthritis (Vereecke *et al*, 2009; Ma & Malynn, 2012; Catrysse *et al*, 2014; Martens & van Loo, 2020). Transgenic A20$^{myel-KO}$ mice were rederived in GF conditions by embryo transfer in the GF and gnotobiotic mouse facility at Ghent University. Since the phenotype of the A20$^{myel-KO}$ model under SPF conditions has already been extensively studied and described (Matmati *et al*, 2011; Walle *et al*, 2014), we here merely focus on the comparison of arthritis features in A20$^{myel-KO}$ mice housed under SPF versus axenic conditions. Histological analysis confirmed destructive arthritis on peripheral joint sections, but we did not observe signs of inflammation in ileum, colon, lung, liver, kidney, and skin sections of SPF A20$^{myel-KO}$ mice (Appendix Fig S5A). However, RT-qPCR data showed significantly increased expression of *Il-1β* in kidney, spleen and knee synovium of A20$^{myel-KO}$ mice, not in colon, ileum, liver, lung, and skin (Appendix Fig S5B).

A20$^{myel-KO}$ mice did not differ in body weight compared to their wild-type littermates, and this for both SPF- and GF-raised mice (Fig 5A). However, macroscopic analysis revealed swollen ankle joints in SPF and GF A20$^{myel-KO}$ mice (Fig 5B). To evaluate arthritis histologically, H&E staining on foot sections was performed and histopathological arthritis features were scored by focusing on the calcaneocuboid joint, the calcaneus and the synovio-entheseal complex (Fig 5C and D). In 5–15-week-old GF- and SPF-raised A20$^{myel-KO}$ mice, immune cell infiltrates were clearly present in the fat pads and the synovium, with occasionally affected cartilage and

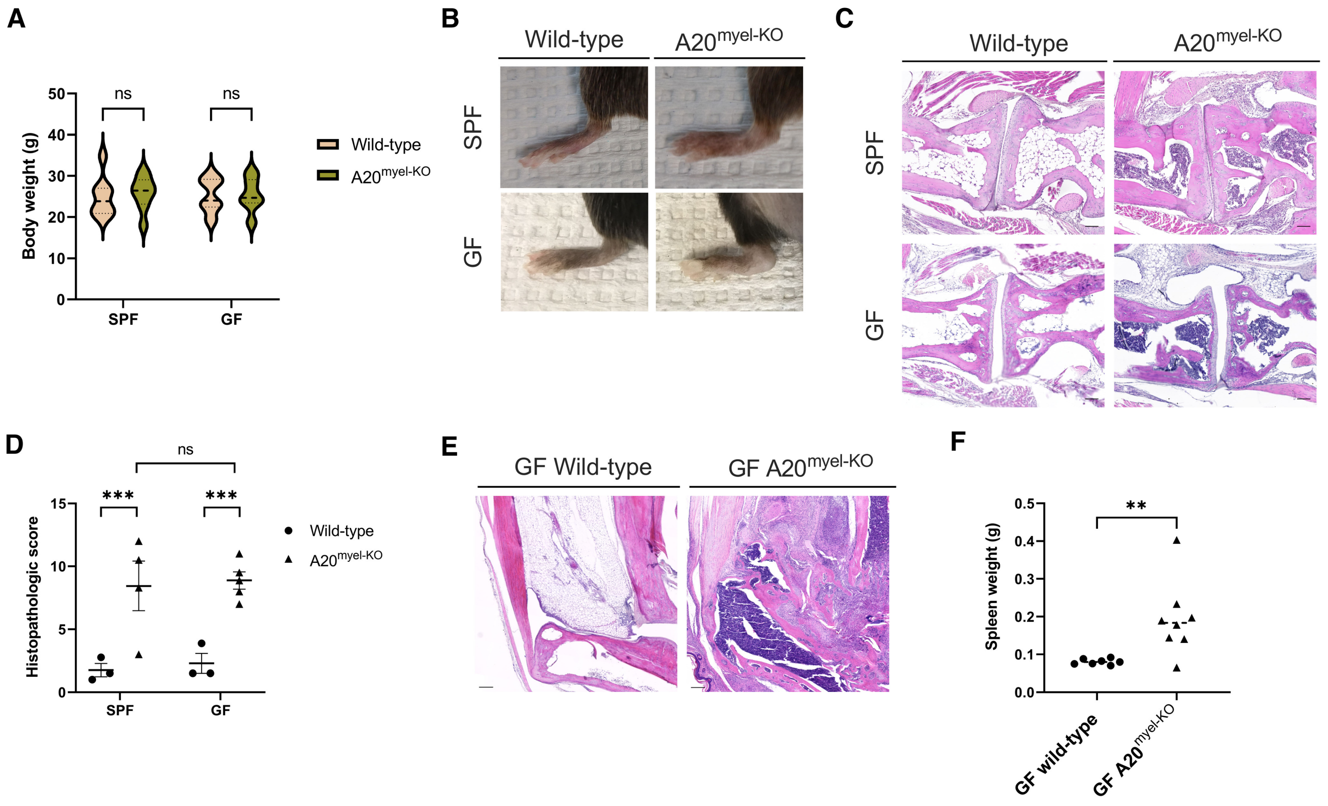

Figure 5. A20$^{myel-KO}$ mice are not rescued from joint inflammation in axenic conditions.

A Body weight between A20$^{myel-KO}$ and wild-type mice does not differ significantly in both housing conditions (SPF wild-type $nF = 5$, $nM = 3$; SPF A20$^{myel-KO}$ $nF = 5$, $nM = 3$; GF wild-type $nF = 7$, $nM = 7$; SPF A20$^{myel-KO}$ $nF = 4$, $nM = 9$).

B Macroscopic image of hind paw of GF and SPF A20$^{myel-KO}$ mouse clearly show swelling of the ankle and toes. (Mice were 26–31 weeks old).

C H&E sections of the calcaneocuboid joint of 5–15 w/o A20$^{myel-KO}$ mice indicate immune cell infiltrates in the synovium and the fat pad and severe bone marrow edema (Scale bars: 100 μm).

D Histopathological scoring of joint inflammation of 5–15 w/o mice quantitatively confirms similar disease development in A20$^{myel-KO}$ SPF and GF mice (SPF wild-type $n = 3$, SPF A20$^{myel-KO}$ $n = 4$; GF wild-type $n = 3$, GF A20$^{myel-KO}$ $n = 5$).

E Histologic images of hind paws of old (44 w/o) GF wild-type (left) versus A20$^{myel-KO}$ (right) mice. The morphology of the paw is completely lost in the transgenic mouse due to severe inflammation (Scale bars: 200 μm).

F Splenomegaly in GF A20$^{myel-KO}$ mice of 15 w/o indicates systemic inflammation in this mouse model (wild-type $n = 7$, A20$^{myel-KO}$ $n = 8$).

Data information: For graph (A), violin plots represent the 25 and 75 percentile with median values as central band. For panels (D, F), data are represented as Mean ± SEM, n = biological replicates. For graph (A, D), two-way ANOVA test was used. For graph (F), two-tailed unpaired t-test was used. ns = P-value > 0.05, * = P-value ≤ 0.05, ** = P-value ≤ 0.01, *** = P-value ≤ 0.001, **** = P-value ≤ 0.0001.
Source data are available online for this figure.

© 2023 The Authors

bone structures and massive bone marrow edema (Fig 5C). In old GF A20$^{myel-KO}$ mice (> 30 weeks), the normal anatomical morphology of the hind paw was completely lost and we observed massive immune cell infiltration and loss of bone, cartilage, and fat pads (Fig 5E). Similar to SPF A20$^{myel-KO}$ mice (Matmati *et al*, 2011), GF A20$^{myel-KO}$ mice showed severe splenomegaly (Fig 5F), confirming a state of systemic inflammation. Together, these data indicate that also in this transgenic mouse model of innate (IL-1β)-driven arthritis, no causative role for the intestinal microbiota can be observed, as GF A20$^{myel-KO}$ mice still develop severe arthritis.

Macrophages and synovial fibroblasts from TNF^{emARE} and A20$^{myel-KO}$ mice are hyperresponsive to DAMP stimulation

Since sterile disease mechanisms drive musculoskeletal pathology in both TNF^{emARE} and A20$^{myel-KO}$ mice, we hypothesize that sterile signals, including DAMPs and mechanical strain, can activate inflammatory responses in stromal and immune cells in the joints. Multiple intracellular DAMPs are known to activate pattern recognition receptors and drive tissue inflammation when released in conditions of cell damage, including HMGB1, S100A8/A9, IL-1α, IL-33, ATP, uric acid (UA), heat shock proteins, mitochondrial DNA, etc. In addition, extracellular matrix components can trigger immune activation and inflammation when released in damaged tissue, including fibronectin, tenascin-C, hyaluronan, etc. (Taniguchi *et al*, 2018; Millerand *et al*, 2019; Danieli *et al*, 2022).

We investigated whether sterile DAMPs can drive inflammatory responses in primary macrophages (bone marrow-derived macrophages, BMDMs) or primary synovial fibroblasts (SFs) derived from A20$^{myel-KO}$ and TNF^{emARE} mice. We stimulated SF and BMDM cells with uric acid (UA), HMGB1, IL-33, IL-1α, S100A8/A9, or with a crude mix of intracellular DAMPs derived from freeze-thawed cells (crude cell lysate). We next performed Luminex Bio-plex cytokine assays on cell supernatant of cells after 48 h of stimulation (Fig EV5). Fibroblasts and BMDMs derived from A20$^{myel-KO}$ and TNF^{emARE} had a baseline activation in unstimulated conditions, indicating that homeostatic inflammatory tone is already elevated in these primary cells. Stimulation of BMDMs derived from old (30–40 weeks) TNF$^{emARE/ARE}$ mice with IL-1α induced significantly increased levels of TNF, compared to cells from wild-type and heterozygous littermate controls. IL-6 production in TNF$^{emARE/ARE}$ BMDMs was significantly higher after stimulation with IL-1α, IL-33, and UA (Fig EV5A). In BMDMs derived from young TNF$^{emARE/ARE}$ mice (7–9 weeks), both TNF and IL-6 secretion was increased upon stimulation with IL-33, HMGB1 and crude cell lysate (Fig EV5B). Similarly, TNF$^{emARE/ARE}$ synovial fibroblasts secreted more IL-6 upon stimulation with crude cell lysate and HMGB1, compared to wild-type and TNF$^{emARE/+}$ control cells (Fig EV5C and D). In A20$^{myel-KO}$ BMDMs, we found higher IL-1β secretion upon stimulation with IL-33 and ATP (Fig EV5E). Together, we show that both macrophages and synovial fibroblasts derived from A20$^{myel-KO}$ and TNF^{emARE} mice secrete elevated levels of inflammatory cytokines in response to multiple DAMPs.

In conclusion, our data demonstrate that in two genetic mouse models of arthritis, driven by either TNF or IL-1β, musculoskeletal disease develops independent of the microbiota, independent of gut inflammation, but instead is driven by joint-specific sterile factors.

Discussion

Clinical observations in human IBD and arthritis suggest a common disease pathophysiology, commonly termed the ‘gut-joint’ axis. The fact that intestinal inflammation, although often subclinical, or an enteric infection, precedes joint inflammation in some patients, may support a causal relationship. Both gut and joint inflammation are associated with intestinal barrier permeability, which is even apparent in non-symptomatic first degree relatives of SpA patients (Hecquet *et al*, 2021).

The strongest genetic risk factor for SpA in humans is the human major histocompatibility complex (*MHC*) class I allele *HLA-B27*, which is believed to contribute to arthritis by promoting intestinal dysbiosis by driving endoplasmic reticulum stress and activation of the unfolded protein response upon HLA-B27 misfolding, and by activating CD8^{+} cytotoxic T cells upon recognition of arthritogenic peptides (Dumas *et al*, 2020; Kavadichanda *et al*, 2021). These arthritogenic peptides may be derived from the intestinal microbiota, and resemble self-peptides (molecular mimicry), which are subsequently targeted by effector T cells and cause joint inflammation (Pedersen & Maksymowych, 2019). Remarkably, HLA-B27 transgenic rats develop spontaneous gut and joint inflammation and are protected in GF conditions (Tautog *et al*, 1994). A recent study identified an arthritogenic strain of *Subdoligranulum* that can drive systemic autoantibody production and joint inflammation in mono-associated mice (Chriswell *et al*, 2022). Other experimental mouse arthritis models, including K/BxN, CIA, and SKG require microbial triggers to drive type-3 immunity and arthritis development (Sakaguchi *et al*, 2003; Rehaume *et al*, 2014; Liu *et al*, 2016; Teng *et al*, 2017; Jubair *et al*, 2018). We, therefore, hypothesize that patients with genetic defects in type-3 immune-associated genes may be more prone to microbial-induced inflammatory pathology, including arthritis. In contrast, inflammatory arthritis caused by increased levels of or response to TNF and IL1-β are less likely to be driven or influenced by microbial triggers. Furthermore, both IBD and SpA are diseases characterized by changes in microbial community structure, composition, and function, termed ‘dysbiosis,’ which is suggested to prime mucosal immune cells, which traffic from the gut to the joints and cause inflammation (Gracey *et al*, 2020; Qaiyum *et al*, 2021). Photoactivatable transgenic Kaede and KikGR mice have provided evidence for trafficking of intestinal immune cells to extraintestinal sites including the joints, and gut-joint trafficking of colonic intraepithelial lymphocytes was demonstrated in TNF$^{\Delta ARE}$ mice (Morton *et al*, 2014; Lefferts *et al*, 2022). Despite various experimental findings supporting a causal link between gut and joint inflammation, it remains unclear whether joint inflammation is critically dependent on microbial triggers or inflammatory cues from the gut. It is noteworthy that half of the SpA patients develop severe axial or peripheral joint inflammation in the absence of subclinical gut inflammation. We, therefore, examined two independent transgenic mouse arthritis models, the TNF^{emARE} model which is driven by TNF, and the A20$^{myel-KO}$ model which is driven by IL-1β. The TNF^{emARE} and A20$^{myel-KO}$ are valuable models not only to unravel the downstream pathogenic mechanisms which are induced by elevated TNF and IL-1β levels, respectively, but also to identify the critical upstream triggers for TNF and IL-1β induction in various tissue compartments. Our data demonstrate that severe arthritis can develop in both mouse models raised in GF conditions. In contrast,

© 2023 The Authors

intestinal inflammation in homozygous $TNF^{emARE/ARE}$ mice is fully microbiota-dependent. We hereby provide evidence that SpA-like arthritis in mice can be induced by sterile factors in the absence of intestinal inflammation and the microbiota. Joint-associated sterile inflammatory triggers include various DAMPs, of which the release is facilitated by mechanical loading, causing damage to the extracellular matrix or cell death in stromal and immune cells of the joint (Nefla *et al*, 2016; Cambré *et al*, 2019; Danieli *et al*, 2022). We could show that both stromal and immune cells in $A20^{myel\text{-}KO}$ and TNF^{emARE} mice are hyperresponsive to stimulation with various DAMPs. *In vivo*, excessive inflammatory responses to DAMPs can promote recruitment of inflammatory immune cells and perpetuation of the inflammatory response through the production of inflammatory cytokines such as TNF, IL-6, IL-1β, and IL-17. The role of mechanical loading in arthritis development was clearly shown in $TNF^{\Delta ARE}$ mice, where hind limb unloading led to reduced entheseal inflammation and prevention of new bone formation (Jacques *et al*, 2014). IBD and SpA are complex multi-factorial diseases influenced by environmental, genetic, and immunological factors, with tightly intertwined inflammatory pathways underlying both diseases. We hypothesize that depending on the type and severity of the underlying genetic predisposition, depending on the microbiota composition, and depending on prior immune education, joint inflammation can be influenced to a varying degree by gut/microbiota-derived mechanisms, but can also develop in response to sterile factors only.

Materials and Methods

Generation of C57Bl6/J-$Tnf^{emARE1Irc}$ mice

C57Bl6/J-$Tnf^{emARE1Irc}$ (in this paper referred to as TNF^{emARE}) mice were generated by the Transgenic Core Facility (TCF) of the VIB-Ugent Center for Inflammation Research (IRC). Guide sequences 5′ GTGCAAATATAAATAGAGGG 3′ (sgRNA1) and 5′ GGAAGGCC GGGGTGTCCTGG 3′ (sgRNA2) were cloned in the BbsI site in the pX330 vector (addgene #42230). For sgRNA synthesis, the T7 promoter sequence was added to sgRNA forward primer and the IVT template generated by PCR amplification using forward primers 5′ TTAATACGACTCACTATAGGTGCAAATATAAATAGAGGG 3′ and 5′ TTAATACGACTCACTATAGGGAAGGCCGGGGTGTCCTGG 3′ for gRNA1 and gRNA2, respectively, and reverse primer 5′ AAAAGC ACCGACTCGGTGCC 3′. The T7-sgRNA PCR product was purified and used as the template for IVT using the MEGAshortscript T7 kit (Thermofisher). Both sgRNAs were purified using the MEGAclear kit (Thermofisher). TNF^{emARE} mice were generated by injecting a mix of gRNA1 (10 ng/μl), gRNA2 (10 ng/μl), Cas9 protein (40 ng/μl; VIB Protein Service Facility) and Cas9 mRNA (20 ng/μl, Thermofisher) in C57BL/6J zygotes. Injected zygotes were incubated overnight in Embryomax KSOM medium (Merck, Millipore) in a CO_2 incubator. The following day, 2-cell embryos were transferred to pseudopregnant B6CBAF1 foster mothers. The resulting pups were screened by PCR over the target region using primers 5′ TCTCATGC ACCACCATCAA 3′ and 5′ GCAGAGGTTCAGTGATGTAG 3′. PCR bands were Sanger sequenced to identify the exact nature of the deletion. Mouse line TNF^{emARE} contains an allele with a deletion of 107 bp in the 3′ UTR of the Tnf gene at Chromosome 17:35418603–35418709 (GRCm39). This mutation is predicted to cause stabilization of the Tnf mRNA.

Animal experiments

TNF^{emARE} mice were generated as described above. Generation of $A20^{myel\text{-}KO}$ mice was described previously (Vereecke *et al*, 2010; Matmati *et al*, 2011). Mice were housed in individually ventilated cages at the campus of UZ Ghent in a specific pathogen-free animal facility. Axenic mice were generated by embryo transfer in axenic recipients at the GF mouse facility of the University of Ghent. Axenic mice were housed under positive-pressure flexible film isolators (North Kent Plastics). All experiments were performed on mice of C57Bl/6 genetic background. All animal experiments were performed and approved according to institutional (Ethical Committee for Animal Experimentation at Ghent University's Faculty of Medicine and Health Science; ECD17-100, ECD18-100, ECD20-94, ECD 21-15), national, and European animal regulations.

Succinate experiment

Sodium succinate dibasic hexahydrate 99% (Sigma-Aldrich; S2378) was given to mice (n = 8/genotype) of approximately 8-week-old ad libitum via drinking water in a concentration of 120 mmol/l, for an average period of 15 weeks. Mice were followed-up by measuring body weight (every 2 weeks) and performing hemoccult fecal tests (week 10 and week 15). Endpoint of the experiment was after 15 weeks of treatment, mice were sacrificed and dissected.

Grip strength test

The Bioseb Grip Strength Test was used to score functional disability in all four paws as well as the two front paws of mice. All scores were corrected for body weight.

CatWalk gait analysis

Noldus CatWalk XT is a gait analysis system for rodents and was used to study differences in walking patterns between different mouse groups. Experiments were set-up with following parameters: minimum run duration of 0.5 s, maximum run duration of 5 s, maximum allowed speed variation at 60%.

Data were gathered and analyzed using the Noldus CatWalk XT software. Text on Movies EV1–EV4 (genotype) was added using Descript 55.1.1 software.

In vivo imaging

PET-CT imaging was performed at the INFINITY lab of University Ghent.

All animals were food deprived for at least 6 h prior to PET imaging. Mice were shortly anesthetized using a mixture of isoflurane and medical oxygen (5% induction, 1.5% maintenance, 0.3 l/min) to insert a catheter in one of the tail veins for tracer injection. Next, animals were intravenously injected with 10 MBq of FDG (Ghent University Hospital, Belgium) dissolved in 200 μl saline. Directly after tracer injection, the catheter was removed, mice were awakened and put into their cages. To reduce FDG uptake in brown fat, a heated blanket was placed under the cage to keep the animals

© 2023 The Authors

warm. In addition, the heated cage was placed in a dark room to minimize tracer uptake into the Harderian glands. Forty minutes after tracer injection, the animals were placed under general anesthesia using an isoflurane mixture (5% induction, 1.5% maintenance, 0.3 l/min) and a 15-min total-body PET scan was acquired on a dedicated small animal PET scanner with sub-mm spatial resolution (B-Cube, Molecubes, Ghent, Belgium). Animals were placed in prone position, receiving further anesthesia through a nose cone. Body temperature was maintained at 37°C by a heated bed. Each PET scan was followed by a total-body spiral high-resolution CT scan (X-Cube, Molecubes). The acquired PET data were iteratively reconstructed into a 192 × 192 × 384 matrix with 400 μm isotropic voxel size. CT data were iteratively reconstructed into a 200 × 200 × 550 matrix with 200 μm isotropic voxel size. PET-CT images were processed and analyzed via Amide software (Loening & Gambhir, 2003).

Ex vivo imaging

Murine paws and spine were dissected, fixed in 4% formaldehyde for 48 h and then kept in 70% EtOH. *Ex vivo* high-resolution X-ray CT imaging of the murine hind paws and spines was performed at the Ghent University Centre for X-ray Tomography (UGCT). The samples were fixed in centrifuge tubes using wet cotton wool to avoid drying out of the samples during the μCT scan. The data were acquired using a commercial high-resolution CT scanner (CoreTom, TESCAN, Ghent, Belgium). For both paws and spines, six samples were imaged. The paws were imaged at a reconstructed voxel size of 7^3 μm^3, while for the spines a voxel size of 45^3 μm^3 was achieved. All samples are scanned using a tube voltage of 120 kV and a hardware filter of 0.5 mm Al. Covering 360°, 2,001 and 1,501 projection images were acquired for the paws and spines, respectively, at an exposure time of 210 ms per projection image. After acquisition, tomographic reconstruction was performed using the proprietary software of the scanner system. The reconstructed volumes were exported as a stack of 16 bit tiff slices, with the gray values representing the local reconstructed attenuation coefficients after rescaling which was fixed for each sample type (spine: −0.3 to 2 cm^{-1}, hind paw: −1 to 2.2 cm^{-1}).

μCT images have been processed using Fiji (Schindelin *et al*, 2012) and the MorpholibJ (Legland *et al*, 2016) plugin before rendering them in 3D. A mask describing the region of interest (hind paw or axial skeleton) has been created by filtering the images using a Gaussian blur with a sigma of 2 on the original image, setting a threshold on the image using the minimum method (Prewitt & Mendelsohn, 1966), filtering out the particles smaller than 50,000 voxels and finally dilating the mask. The mask is then applied on the original image to remove the noise signal. The rendering in three dimensions has been done in Napari, a multidimensional image viewer for Python, using the iso-surface rendering.

Histology and histopathologic scoring

Skin, lung, liver, and kidney were dissected and fixed in 4% formaldehyde for 24 h. Paraffin sections were stained with Hematoxylin-Eosin (H&E) to evaluate inflammation in these organs. Images were acquired using Zeiss Axioscan and Zen Blue software.

Murine gut was dissected and fixed in 4% formaldehyde for 24 h. Paraffin sections were stained with Hematoxylin-Eosin (H&E) and ileal TNF^{emARE} sections were scored blindly by assessing villus architectural distortion (0–4), goblet cell depletion (0–4), and mononuclear cell infiltration (0–4) resulting in an overall score of 0–12 (Appendix Table S1). Images were acquired using Zeiss Axioscan and Zen Blue software.

Murine paws and spine were dissected, fixed in 4% formaldehyde for 48 h and then decalcified using 5% formic acid for 8 consecutive days. Paraffin sections were stained with H&E for evaluation of inflammation and bone erosions. Disease development in hind paws was scored blindly by assessing the parameters in Appendix Table S2, based on Yang–Hamilton (Yang & Hamilton, 2001) scoring and SKG scoring (Ruutu *et al*, 2012). To quantify inflammation in the spine, H&E sections were scored for the extent of immune cell infiltration (0–3) along the longitudinal ligament and in the intervertebral discs at the thoracic segment (Appendix Table S3). Images were acquired using Zeiss Axioscan and Zen Blue software.

Immunofluorescent staining

Ileal sections were incubated in antigen retrieval solution (Dako; H3300, 1/100) while being heated using a PickCell Electric Cooker. After cooling down, ileal sections were incubated with blocking buffer (goat serum; Sigma; S26-100ML, 1/100) for 30 min at room temperature. Subsequently, sections were stained with primary antibody (rabbit anti-lysozyme (Dako; EC 3.2.1.17, 1/500) or rabbit anti-DCAMKL1 (Abcam; Ab31704, 1/500)) overnight at 4°C. After washing the slides with PBS, ileum sections were counterstained with DAPI (Thermofisher; D21490, 1/1,000), UEA-1 Fluorescein (Vector laboratories; FL-1061, 1/1,000), and WGA (ThermoFisher; W11261, 1/200) and incubated with secondary antibody goat anti-rabbit Alexa Fluor 568 (Thermofisher; A11036, 1/1,000). After 1 h, slides were mounted and later imaged with Zeiss AxioScan (10×) and processed with Zen Blue (anti-DCAMKL1 staining) or imaged with Zeiss LSM880 Airyscan (60×) and processed with Zen Black software (anti-lysozyme staining).

Flow cytometry

Small intestine

Lamina propria isolation of small intestine samples of SPF TNF^{emARE} (wild-type $n = 5$, $TNF^{emARE/+}$ $n = 5$, $TNF^{emARE/ARE}$ $n = 5$) mice was performed as described previously (Bain & Mowat, 2012). The isolated cells were used for extra- and intracellular staining for representative markers of the T cells and myeloid cells, in two separate panels (1×10^6 cell/sample). Samples were analyzed using the five-laser BD LSRFortessa.

Synovium

Flow cytometry was performed on synovium of SPF TNF^{emARE} mice (wild-type $n = 8$, $TNF^{emARE/+}$ $n = 8$, $TNF^{emARE/ARE}$ $n = 8$). Mice were sacrificed by cervical dislocation, one hind leg was cut above the knee and the skin was removed. The patella and patellar tendon were isolated to collect knee synovium and kept in RPMI medium. Synovium from tibiotalar joint was isolated to collect foot synovium and kept in RPMI medium. For flow cytometry analysis, the synovia of two mice were pooled to have enough cells. To make

© 2023 The Authors

the enzymatic digest, a final concentration of 0.75 mg/ml of type VIII Collagenase (Sigma, C2139) and 1 mg/ml Dispase (Gibco, 17105041) in warm RPMI medium is needed. A stirrer was placed in the tube containing synovial tissue and the enzymatic digest, and placed on a shaker incubator for 30 min (500 rpm). Following completion of the incubation time, cells and medium were passed through a 100-μm strainer. The strainer was then washed with cold 2% FBS in PBS to collect all cells. Next, tubes were centrifuged at 393 *g* for 7 min at 4°C and cells resuspended in 0.5 ml 2%FBS/PBS and counted. The isolated cells were used for extracellular staining for representative markers of the myeloid cells (1×10^6 cells/sample). Samples were analyzed using the five-laser BD LSRFortessa.

For the myeloid compartment, cells were stained with Fixable Viability Dye eFluor 506 (eBioscience; 65-0866-14, 1/300) for live/dead separation and only extracellular staining was done using following antibodies: CD19 (eBioscience; 15-0193-82, 1/400), CD3 (eBioscience; 15-0031-82, 1/200), NK1.1 (BioLegend; 108716, 1/200), anti-CD45 Alexa Fluor 700 (eBioscience 56-0451-82, 1/800), anti-Ly6G PercpCy5.5 (BD; 560602, 1/200), anti-Ly6C-APC (eBIoscience; 17-5932-80, 1/200), anti-Siglec F BUV395 (BD; 740280, 1/200), anti-CD11b BV506 (BD; 563015, 1/600), anti-CD64 BV711 (BioLegend; 139311, 1/100), anti-F4/80 Biotin (eBioscience; 13-4801-82, 1/100), Streptavidin BV421 (BioLegend; 405226, 1/1,000), anti-CD11c PE-eFluor 610 (eBioscience; 61-0114-82, 1/300), anti MHC class II APC-eFLuor 780 (eBioscience; 47-5321-80, 1/800), anti-XCR1 BV650 (Biolegend, 148220, 1/400), and anti-SIRPa PE-Cy7 (Biolegend, 144007, 1/100). For the T-cell compartment, cells were stained with 7-AAD for live/dead separation, extracellular staining with anti-CD3 APC (eBioscience; 17-0031-83, 1/100), anti-CD4 APC Cy-7(BD; 552051, 1/200), and anti-CD8 V500 (BD; 560776, 1/100). Thereafter, cells were fixed and permeabilized using the Foxp3 Transcription Factor Staining Buffer Set (eBioscience; 00-5523-00). Finally, cells were stained intracellular with anti-Foxp3 Alexa Fluor 488 (eBioscience; 53-5773-82, 1/100), anti-RORγt BV421 (BD; 562894, 1/100), anti-Tbet PE-Cy7 (Invitrogen, 25-5825-82, 1/100), and anti-GATA3 PE (Invitrogen, 12-9966-42, 1/100). Flow cytometry data were analyzed using FlowJo Software 10.8.1 and a sequential gating strategy. Regarding the tSNE plots, for the myeloid panel the analysis was performed on a total of 45,000 $CD45^+$ cells excluding the lineage (CD3, CD19, NK1.1) and for the T cell panel the analysis was performed on 173,600 $CD3^+$ cells. The cells were exported, concatenated, and analyzed with FitSNE (Fast Fourrier Transform-accelerated Interpolation-based t-SNE) Flowjo plugin (version 0.5.1; perplexity: 20, Max iterations: 1,000). Following dimensional reduction, coordinates for each t-SNE dimension (i.e., tSNE1 and tSNE2) in the two-dimensional plots were determined and integrated as novel parameters. For the myeloid panel, the bar plots showing percentages of immune cells represent proportions of every parent population (Fig 1H). For the T cell panel, $CD8^+$ and $CD4^+$ cells are presented as percentages of the $CD3^+$ parent population, $CD4^+RORgt^+$ and $CD4^+Foxp3^+$ cells are presented as percentages of the $CD4^+$ population (Fig 1J). Gating strategy for both myeloid and T cell panel can be found in Appendix Fig S4.

ELISA

Blood was collected postmortem via cardiac puncture. Serum was isolated by 8 min centrifugation at 8,000 *g* and stored at −20°C. Plates were coated with capture antibody anti-mouse/rat TNFα (eBioscience; 14-7423-85, 1/500) overnight at 4°C. Thereafter, plates were blocked with 0.1% casein blocking buffer for 2 h by 27°C. Next, samples were added (undiluted) and incubation took 2 h by 27°C. After washing, detection antibody anti-mouse/rat TNFα (eBioscience; 13-7341-85, 1/500) was added, followed by Avidin HRP enzyme (eBioscience; 18-4100-51, 1/100) and TMB substrate (BD Biosciences; 555214, 1/1). To end the reaction, stop solution (H2SO4, 1 M) was added. Absorbance was read immediately at 450 nm. Concentrations were calculated in Graphpad Prism based on the standard curve.

RNA isolation and quantitative real-time PCR (qPCR)

RNA isolation ileum, colon, skin, kidney, lung, liver, spleen, bone

Tissue was lysed and homogenized using RLT and β-mercaptoethanol and the TissueLyser II (Qiagen). Total RNA was isolated using the RNeasy Mini Kit (Qiagen; 74106), according to the manufacturer's instructions.

RNA isolation synovium

To lyse the synovial tissue, 800 μl TRIsure (Bioline; BIO-38033) was added and a mixer was used. After lysing, 180 μl chloroform was added and samples were centrifuged for 15 min at max speed by 4°C to create different phases. The aqueous phase was transferred into a new tube and RNA isolation was proceeded using the RNeasy Mini kit (Qiagen, 74106).

The synthesis of cDNA was performed using QuantiTect® Reverse Transcription Kit (Qiagen; 205311), following the manufacturer's protocol. For qPCR, SensiFAST SYBR NO-ROX (BioLine; BIO-98005) and specific primers (*Tnf* fwd TGTCTTTGAGATCCATGCCGT; *Tnf* rev TCAAAATTCGAGTGACAAGCCTG and *Il-1β* fwd CACCTCACAAGCAGAGCACAAG; *Il-1β* rev GCATTAGAAACAGTCCAGCCCATAC) were used on LightCycler 480 (Roche). The reactions were performed in triplicates and the results were analyzed with $qbase^+$ software. As housekeeping genes, GAPDH, Actb, Tbp, Ubc, Hprt1, and Rpl13a were used.

Isolation, cultivation, and stimulation of murine synovial fibroblasts

Under sterile conditions, mice were sacrificed and the paws were cut above the ankle. The skin, sinews, and toes were removed and the joints were transferred to a sterile glass bottle with 5 ml of digestion medium (DMEM with 4.5 g/l glucose + 1 mg/ml Collagenase IV (Worthington; LS004188)) and a sterile small agitator. Incubation took 45–60 min at 37°C on a magnetic stirrer. The cell suspension was then transferred to a 15-ml falcon and the cell suspension was centrifuged for 10 min at 456 *g*. The supernatant was discarded and the pellet resuspended in 10–15 ml of cultivation medium (DMEM with 4.5 g/l glucose + 10% of heat-inactivated FCS (Biochrom AG; S0115) + 1% Penicillin/Streptomycin (Sigma-Aldrich; P4333)). Next, the cells were seeded in T75 flasks and cultivated until confluent. Synovial fibroblasts were cultivated at 37°C, 5% CO_2, 95% relative humidity. Cells were split using Trypsin–EDTA solution, and only passages 3–5 were used for stimulation experiments.

After 3–5 passages, cells were seeded and stimulated for 48 h with either HMGB1 (Abcam; ab255799, 100 ng/ml), uric acid

© 2023 The Authors

(Sigma Aldrich, U2625-25G, 200 μM), S100A8/A9 (R&D Systems; 8916-S8, 100 ng/ml), a combination of HMGB1, uric acid, and S100A8/A9 or a crude mix of intracellular DAMPs derived from freeze-thawed cells (crude cell lysate, 2,500,000 cells/ml).

Isolation, cultivation, and stimulation of bone marrow-derived macrophages (BMDMs)

For isolation and cultivation of BMDMs, mice were sacrificed and hind legs were dislocated to preserve the full bone. The skin and muscles were removed, tibia and femur were cut at the edges. In a Petri dish with fresh medium (RPMI +1% FCS), marrow from the bones was flushed with a 1-ml syringe with 26G needle. The cell solution was then passed through a 100 mm strainer and centrifuged for 5 min at 1,000 rpm. The pellet was resuspended in 3 ml ACK lysis buffer (Lonza; BP10-548E) and incubated for 2–3 min at RT. 10 ml PBS was then added to neutralize the lysis buffer and the solution was again centrifuged for 5 min at 233 *g*. Next, the pellet was resuspended in 30 ml culture medium (RPMI +10% FCS + Penicillin/Streptomycin) + 40 ng/ml m-CSF and cells were seeded. On day 3, 1 ml of fresh culture medium +400 ng/ml m-CSF (final concentration: 40 g/ml) was added to the culture. On day 5, culture medium was refreshed and supplemented with 40 ng/ml m-CSF. On day 7, stimulation assays were performed.

TNF^{emARE}

On day 7, cells were seeded and stimulated for 48 h with either IL-33 (BioLegend; 580502, 10 ng/ml), IL-1α (R&D Systems; 400-ML, 10 ng/ml), HMGB1 (Abcam; ab255799, 100 ng/ml), uric acid (Sigma Aldrich, U2625-25G, 200 μM), or a crude mix of intracellular DAMPs derived from freeze-thawed cells (crude cell lysate, 2,500,000 cells/ml).

$A20^{myel\text{-}KO}$

On day 7, cells were seeded and stimulated for 3 h with IL-33 (BioLegend; 580502, 10 ng/ml), followed by ATP (Sigma-Aldrich; 1191-5GM, 3 mM) for 1 h for NLRP3 activation.

Quantification of cytokines by Luminex technology

After stimulation of BMDM and synovial fibroblast cultures, cytokine concentrations (in culture medium were determined by magnetic bead-based multiplex assays using Luminex technology (BioRad) on the Bio-plex 200 system (BioRad), according to the manufacturer's instructions. Bio-plex was performed for quantification of cytokines IL-6 (BioPlex mouse IL-6 assay; BioRad, 171G5007M), TNFa (Bio-Plex Pro Mouse Cytokine TNFa assay; BioRad, 171G5023M), and IL1-β (Bio-Plex Pro Mouse Cytokine IL1-beta; BioRad, 171G5002M).

Statistics

Statistical analysis was performed using analysis of variance (ANOVA) in Prism V9.2.0. Graphics were created using GraphPad Prism V9.2.0. Data are presented as mean ± SEM and *P*-values below 0.005 were considered statistically significant. Interpretation of asterisks on graphics: ns = P-value > 0.05, * = P-value ≤ 0.05, ** = P-value ≤ 0.01, *** = P-value ≤ 0.001, **** = P-value ≤ 0.0001.

Body weight kinetic analysis

Body weights were analyzed as repeated measurements using the method of residual maximum likelihood (REML), as implemented in Genstat version 22. A linear mixed model (random terms underlined) of the form: body weight = constant + gender + genotype + time + gender × time + genotype × time + subject × time was fitted to the body weight data. The term subject × time represents the residual error term with dependent errors because the repeated measurements are taken in the same individual, causing correlations among observations. The uniform correlation structure was selected as best model fit based on the Akaike Information Coefficient. Times of measurement were set as equally spaced. The significance of the fixed main and interaction terms in the model, and of pairwise comparisons between genotypes across the time series, were assessed by an approximate *F*-test as implemented in Genstat version 22 (Gentstat.co.uk).

The paper explained

Problem

Intestinal and joint inflammation frequently coexist, with a notable link between inflammatory bowel disease (IBD) and joint inflammation in patients. Additionally, individuals with spondyloarthritis (SpA) often exhibit subclinical gut inflammation, a fraction of which evolves into full-blown IBD. These clinical observations have sparked several hypotheses proposing a mechanistic pathophysiological connection between gut and joint inflammation. Moreover, IBD, SpA, and rheumatoid arthritis (RA) are characterized by alterations in the community structure of the intestinal microbiota, termed dysbiosis, which is believed to contribute to both gut and joint pathology.

Results

In this study, we investigated the role of the intestinal microbiota in the development of gut and joint inflammation. For this, we utilized transgenic mouse models and germ-free (GF) mouse technology. We developed a novel mouse model, TNF^{emARE} mice, which exhibit TNF-driven gut and joint inflammation, and characterized these mice under colonized (specific pathogen-free, SPF) and GF conditions. SPF-raised $TNF^{emARE/ARE}$ mice develop musculoskeletal pathology in both peripheral and axial regions, as well as ileitis. Surprisingly, when maintained under GF conditions, $TNF^{emARE/ARE}$ mice are completely protected from intestinal inflammation, yet still developed severe inflammation in the axial and peripheral joints. Furthermore, we investigated transgenic $A20^{myel\text{-}KO}$ mice, which develop IL-1β-driven arthritis in the absence of intestinal inflammation, and observed that GF conditions did not prevent joint inflammation in these mice.

Impact

Our findings provide compelling evidence that the presence of the intestinal microbiota is crucial for TNF-mediated intestinal inflammation, yet dispensable for TNF- and IL-1β-driven joint inflammation. Notably, arthritis develops even in axenic conditions, suggesting that sterile mechanisms, rather than microbial factors, drive joint inflammation. This study establishes a clear mechanistic disconnection between gut and joint inflammation, highlighting the potential for tissue-specific therapeutic interventions targeting the underlying disease-driving triggers.

© 2023 The Authors

Data availability

The datasets produced in this study are available in the following databases:

- Microscopic images: BioImage Archive S-BIAD813 (https://www.ebi.ac.uk/biostudies/bioimages/studies/S-BIAD813).
- Flow cytometry data: BioStudies S-BSST1139 (https://www.ebi.ac.uk/biostudies/studies/S-BSST1139).

Expanded View for this article is available online.

Acknowledgements

We would like to thank the HMI lab and the Dirk Elewaut laboratory for critical discussion and providing expert input, Kelly Lemeire from the IRC Immunohistochemistry core for assisting in staining protocols, Marnik Vuylsteke for assisting in statistical analysis, the VIB Bioimaging Core Ghent for assisting in data acquisition and providing scripts to analyze microscopy data, the Animal House Facility staff in MRBII UZ Ghent for support with animal maintenance, the IRC Transgenic Core Facility for generating TNF^{emARE} mice and for rederiving TNF^{emARE} and $A20^{myel-KO}$ mice germ-free, in collaboration with the Ghent germ-free and gnotobiotic mouse facility. We thank the VIB Flow Cytometry core facility for flow cytometry experiments and analyses. We thank Ghent University (BOF-01N01019, GOA031-22BOF, STA010-19BOF), FWO (EOS-G0H2522N-40007505), and the FOREUM Foundation for Research in Rheumatology for funding this project.

Author contributions

Alexandra Thiran: Data curation; formal analysis; validation; investigation; visualization; methodology; writing – original draft; project administration; writing – review and editing. **Ioanna Petta:** Formal analysis; investigation; methodology. **Gillian Blancke:** Data curation; formal analysis; investigation. **Marie Thorp:** Data curation; investigation. **Guillaume Planckaert:** Data curation; investigation. **Maude Jans:** Data curation; investigation. **Vanessa Andries:** Investigation. **Korneel Barbry:** Methodology. **Elisabeth Gilis:** Investigation; methodology. **Julie Coudenys:** Investigation; methodology. **Tino Hochepied:** Investigation; methodology. **Christian Vanhove:** Investigation; methodology. **Eric Gracey:** Investigation; methodology. **Emilie Dumas:** Investigation. **Teddy Manuelo:** Investigation. **Ivan Josipovic:** Methodology. **Geert van Loo:** Conceptualization; writing – review and editing. **Dirk Elewaut:** Supervision; writing – review and editing. **Lars Vereecke:** Conceptualization; resources; supervision; funding acquisition; investigation; methodology; writing – original draft; project administration; writing – review and editing.

Disclosure and competing interests statement

The authors declare that they have no conflict of interest.

References

Bain CC, Mowat AMI (2012) CD200 receptor and macrophage function in the intestine. *Immunobiology* 217: 643–651

Banerjee A, Herring CA, Chen B, Kim H, Simmons AJ, Southard-Smith AN, Allaman MM, White JR, Macedonia MC, Mckinley ET *et al* (2020) Succinate produced by intestinal microbes promotes specification of tuft cells to suppress ileal inflammation. *Gastroenterology* 159: 2101–2115

Barkhodari A, Lee KE, Shen M, Shen B, Yao Q (2022) Inflammatory bowel disease: focus on enteropathic arthritis and therapy. *Rheumatol Immunol Res* 3: 69–76

Brakenhoff LKPM, van der Heijde DM, Hommes DW, Huizinga TWJ, Fidder HH (2010) The joint-gut axis in inflammatory bowel diseases. *J Crohns Colitis* 4: 257–268

Breban M, Tap J, Leboime A, Said-Nahal R, Langella P, Chiocchia G, Furet JP, Sokol H (2017) Faecal microbiota study reveals specific dysbiosis in spondyloarthritis. *Ann Rheum Dis* 76: 1614–1622

Cambré I, Gaublomme D, Schryvers N, Lambrecht S, Lories R, Venken K, Elewaut D (2019) Running promotes chronicity of arthritis by local modulation of complement activators and impairing T regulatory feedback loops. *Ann Rheum Dis* 78: 787–795

Carron P, Varkas G, Cypers H, Van Praet L, Elewaut D, Van Den Bosch F (2017) Anti-TNF-induced remission in very early peripheral spondyloarthritis: the CRESPA study. *Ann Rheum Dis* 76: 1389–1395

Catrysse L, Vereecke L, Beyaert R, van Loo G (2014) A20 in inflammation and autoimmunity. *Trends Immunol* 35: 22–31

Chriswell ME, Lefferts AR, Clay MR, Hsu AR, Seifert J, Feser ML, Rims C, Bloom MS, Bemis EA, Liu S *et al* (2022) Clonal IgA and IgG autoantibodies from individuals at risk for rheumatoid arthritis identify an arthritogenic strain of Subdoligranulum. *Sci Transl Med* 14: eabn5166

Ciccia F, Guggino G, Rizzo A, Alessandro R, Luchetti MM, Milling S, Saieva L, Cypers H, Stampone T, Di Benedetto P *et al* (2017) Dysbiosis and zonulin upregulation alter gut epithelial and vascular barriers in patients with ankylosing spondylitis. *Ann Rheum Dis* 76: 1123–1132

Danieli MG, Antonelli E, Piga MA, Claudi I, Palmeri D, Tonacci A, Allegra A, Gangemi S (2022) Alarmins in autoimmune diseases. *Autoimmun Rev* 21: 103142

Dumas E, Venken K, Rosenbaum JT, Elewaut D (2020) Intestinal microbiota, HLA-B27, and spondyloarthritis: dangerous liaisons. *Rheum Dis Clin North Am* 46: 213–224

Gracey E, Vereecke L, McGovern D, Fröhling M, Schett G, Danese S, De Vos M, Van den Bosch F, Elewaut D (2020) Revisiting the gut–joint axis: links between gut inflammation and spondyloarthritis. *Nat Rev Rheumatol* 16: 415–433

Guggino G, Mauro D, Rizzo A, Alessandro R, Raimondo S, Bergot AS, Rahman MA, Ellis JJ, Milling S, Lories R *et al* (2021) Inflammasome activation in ankylosing spondylitis is associated with gut dysbiosis. *Arthritis Rheumatol* 73: 1189–1199

Hecquet S, Totoson P, Prati C, Wendling D, Demougeot C, Verhoeven F (2021) Intestinal permeability in spondyloarthritis and rheumatoid arthritis: a systematic review of the literature. *Semin Arthritis Rheum* 51: 712–718

Jacques P, Lambrecht S, Verheugen E, Pauwels E, Kollias G, Armaka M, Verhoye M, Van Der Linden A, Achten R, Lories RJ *et al* (2014) Proof of concept: enthesitis and new bone formation in spondyloarthritis are driven by mechanical strain and stromal cells. *Ann Rheum Dis* 73: 437–445

Jubair WK, Hendrickson JD, Severs EL, Schulz HM, Adhikari S, Ir D, Pagan JD, Anthony RM, Robertson CE, Frank DN *et al* (2018) Modulation of inflammatory arthritis in mice by gut microbiota through mucosal inflammation and autoantibody generation. *Arthritis Rheumatol* 70: 1220–1233

Kavadichanda CG, Geng J, Bulusu SN, Negi VS, Raghavan M (2021) Spondyloarthritis and the human leukocyte antigen (HLA)-B*27 connection. *Front Immunol* 12: 601518

© 2023 The Authors

Kontoyiannis D, Pasparakis M, Pizarro TT, Cominelli F, Kollias G (1999) Impaired On/Off regulation of TNF biosynthesis in mice lacking TNF AU-rich elements. *Immunity* 10: 387 – 398

Kopylov U, Starr M, Watts C, Dionne S, Girardin M, Seidman EG (2018) Detection of Crohn disease in patients with spondyloarthropathy: the SpACE capsule study. *J Rheumatol* 45: 498 – 505

Lefferts AR, Norman E, Claypool DJ, Kantheti U, Kuhn KA (2022) Cytokine competent gut-joint migratory T Cells contribute to inflammation in the joint. *Front Immunol* 13: 932393

Legland D, Arganda-Carreras I, Andrey P (2016) MorphoLibJ: integrated library and plugins for mathematical morphology with ImageJ. *Bioinformatics* 32: 3532 – 3534

Leirisalo-Repo M, Turunen U, Stenman S, Helenius P, Seppälä K (1994) High frequency of silent inflammatory bowel disease in spondylarthropathy. *Arthritis Rheum* 37: 23 – 31

Liu X, Zeng B, Zhang J, Li W, Mou F, Wang H, Zou Q, Zhong B, Wu L, Wei H *et al* (2016) Role of the gut microbiome in modulating arthritis progression in mice. *Sci Rep* 6: 30594

Loening AM, Gambhir SS (2003) AMIDE: a free software tool for multimodality medical image analysis. *Mol Imaging* 2: 131 – 137

Lori Broderick HMH (2022) IL-1 and autoinflammatory disease: biology, pathogenesis and therapeutic targeting. *Nat Rev Rheumatol* 18: 448 – 463

Ma A, Malynn BA (2012) A20: linking a complex regulator of ubiquitylation to immunity and human disease. *Nat Rev Immunol* 12: 774 – 785

Makita S, Takatori H, Nakajima H (2021) Post-transcriptional regulation of immune responses and inflammatory diseases by RNA-binding ZFP36 family proteins. *Front Immunol* 12: 711633

Malcova H, Milota T, Strizova Z, Cebecauerova D, Striz I, Sediva A, Horvath R (2021) Interleukin-1 blockade in polygenic autoinflammatory disorders: where are we now? *Front Pharmacol* 11: 619273

Martens A, van Loo G (2020) A20 at the crossroads of cell death, inflammation, and autoimmunity. *Cold Spring Harb Perspect Biol* 12: a036418

Matmati M, Jacques P, Maelfait J, Verheugen E, Kool M, Sze M, Geboes L, Louagie E, Guire CM, Vereecke L *et al* (2011) A20 (TNFAIP3) deficiency in myeloid cells triggers erosive polyarthritis resembling rheumatoid arthritis. *Nat Genet* 43: 908 – 912

Mielants H, Veys EM, Cuvelier C, De Vos M, Goemaere S, De Clercq L, Schatteman L, Elewaut D (1995a) The evolution of spondyloarthropathies in relation to gut histology. II. Histological aspects. *J Rheumatol* 22: 2279 – 2284

Mielants H, Veys EM, Cuvelier C, De Vos M, Goemaere S, De Clercq L, Schatteman L, Gyselbrecht L, Elewaut D (1995b) The evolution of spondyloarthropathies in relation to gut histology. III. Relation between gut and joint. *J Rheumatol* 22: 2279 – 2284

Mielants H, Veys EM, De Vos M, Cuvelier C, Goemaere S, De Clercq L, Schatteman L, Elewaut D (1995c) The evolution of spondyloarthropathies in relation to gut histology. I. Clinical aspects. *J Rheumatol* 22: 2279 – 2284

Millerand M, Berenbaum F, Jacques C (2019) Danger signals and inflammaging in osteoarthritis. *Clin Exp Rheumatol* 37: 48 – 56

Morton AM, Sefik E, Upadhyay R, Weissleder R, Benoist C, Mathis D (2014) Endoscopic photoconversion reveals unexpectedly broad leukocyte trafficking to and from the gut. *Proc Natl Acad Sci USA* 111: 6696 – 6701

Nefla M, Holzinger D, Berenbaum F, Jacques C (2016) The danger from within: alarmins in arthritis. *Nat Rev Rheumatol* 12: 669 – 683

Ossum AM, Palm Ø, Cvancarova M, Solberg IC, Vatn M, Moum B, Høivik ML (2018) Peripheral arthritis in patients with long-term inflammatory bowel disease. Results from 20 years of follow-up in the IBSEN study. *Scand J Gastroenterol* 53: 1250 – 1256

Pedersen SJ, Maksymowych WP (2019) The pathogenesis of ankylosing spondylitis: an update. *Curr Rheumatol Rep* 21: 58

Polykratis A, Martens A, Eren RO, Shirasaki Y, Yamagishi M, Yamaguchi Y, Uemura S, Miura M, Holzmann B, Kollias G *et al* (2019) A20 prevents inflammasome-dependent arthritis by inhibiting macrophage necroptosis through its ZnF7 ubiquitin-binding domain. *Nat Cell Biol* 21: 731 – 742

Prewitt JMS, Mendelsohn ML (1966) The analysis of cell images. *Ann N Y Acad Sci* 128: 1035 – 1053

Qaiyum Z, Lim M, Inman RD (2021) The gut-joint axis in spondyloarthritis: immunological, microbial, and clinical insights. *Semin Immunopathol* 43: 173 – 192

Rehaume LM, Mondot S, Aguirre De Cárcer D, Velasco J, Benham H, Hasnain SZ, Bowman J, Ruutu M, Hansbro PM, McGuckin MA *et al* (2014) ZAP-70 genotype disrupts the relationship between microbiota and host, leading to spondyloarthritis and ileitis in SKG mice. *Arthritis Rheum* 66: 2780 – 2792

Rogler G, Singh A, Kavanaugh A, Rubin DT (2021) Extraintestinal manifestations of inflammatory bowel disease: current concepts, treatment, and implications for disease management. *Gastroenterology* 161: 1118 – 1132

Roulis M, Bongers G, Armaka M, Salviano T, He Z, Singh A, Seidler U, Becker C, Demengeot J, Furtado GC *et al* (2016) Host and microbiota interactions are critical for development of murine Crohn's-like ileitis. *Mucosal Immunol* 9: 787 – 797

Ruutu M, Thomas G, Steck R, Degli-Esposti MA, Zinkernagel MS, Alexander K, Velasco J, Strutton G, Tran A, Benham H *et al* (2012) β-Glucan triggers spondylarthritis and Crohn's disease-like ileitis in SKG mice. *Arthritis Rheum* 64: 2211 – 2222

Sakaguchi N, Takahashi T, Hata H, Nomura T, Tagami T, Yamazaki S, Sakihama T, Matsutani T, Negishi I, Nakatsuru S *et al* (2003) Altered thymic T-cell selection due to a mutation of the ZAP-70 gene causes autoimmune arthritis in mice. *Nature* 426: 454 – 460

Schaubeck M, Clavel T, Calasan J, Lagkouvardos I, Haange SB, Jehmlich N, Basic M, Dupont A, Hornef M, Von Bergen M *et al* (2016) Dysbiotic gut microbiota causes transmissible Crohn's disease-like ileitis independent of failure in antimicrobial defence. *Gut* 65: 225 – 237

Schindelin J, Arganda-Carreras I, Frise E, Kaynig V, Longair M, Pietzsch T, Preibisch S, Rueden C, Saalfeld S, Schmid B *et al* (2012) Fiji: an open-source platform for biological-image analysis. *Nat Methods* 9: 676 – 682

Sheth T, Pitchumoni CS, Das KM (2015) Management of musculoskeletal manifestations in inflammatory bowel disease. *Gastroenterol Res Pract* 2015: 387891

Taniguchi N, Kawakami Y, Maruyama I, Lotz M (2018) HMGB proteins and arthritis. *Hum Cell* 31: 1 – 9

Taurog J, Chhabra A, Colbert RA (2016) Ankylosing spondylitis and axial spondyloarthritis. *N Engl J Med* 374: 2563 – 2574

Tautog JD, Richardson JA, Croft JT, Simmons WA, Zhou M, Luis Fernáindez-Sueiro J, Balish E, Hammerll RE (1994) The germfree state prevents development of gut and joint inflammatory disease in HLA-B27 transgenic rats. *J Exp Med* 180: 2359 – 2364

Teng F, Felix KM, Bradley CP, Naskar D, Ma H, Raslan WA, Wu HJJ (2017) The impact of age and gut microbiota on Th17 and Tfh cells in K/BxN autoimmune arthritis. *Arthritis Res Ther* 19: 188

Tito RY, Cypers H, Joossens M, Varkas G, Van Praet L, Glorieus E, Van den Bosch F, De Vos M, Raes J, Elewaut D (2017) Brief report: dialister as a microbial marker of disease activity in spondyloarthritis. *Arthritis Rheum* 69: 114 – 121

 © 2023 The Authors

Van Praet L, Van Den Bosch FE, Jacques P, Carron P, Jans L, Colman R, Glorieus E, Peeters H, Mielants H, De Vos M *et al* (2013) Microscopic gut inflammation in axial spondyloarthritis: a multiparametric predictive model. *Ann Rheum Dis* 72: 414 – 417

Vereecke L, Beyaert R, van Loo G (2009) The ubiquitin-editing enzyme A20 (TNFAIP3) is a central regulator of immunopathology. *Trends Immunol* 30: 383 – 391

Vereecke L, Sze M, Guire CM, Rogiers B, Chu Y, Schmidt-Supprian M, Pasparakis M, Beyaert R, van Loo G (2010) Enterocyte-specific A20 deficiency sensitizes to tumor necrosis factor–induced toxicity and experimental colitis. *J Exp Med* 207: 1513 – 1523

Vereecke L, Vieira-Silva S, Billiet T, Van Es JH, Mc Guire C, Slowicka K, Sze M, Van Den Born M, De Hertogh G, Clevers H *et al* (2014) A20 controls intestinal homeostasis through cell-specific activities. *Nat Commun* 5: 5103

Walle LV, Van Opdenbosch N, Jacques P, Fossoul A, Verheugen E, Vogel P, Beyaert R, Elewaut D, Kanneganti TD, Van Loo G *et al* (2014) Negative regulation of the NLRP3 inflammasome by A20 protects against arthritis. *Nature* 512: 69 – 73

Yang YH, Hamilton JA (2001) Dependence of interleukin-1-induced arthritis on granulocyte-macrophage colony-stimulating factor. *Arthritis Rheum* 44: 111 – 119

Zaiss MM, Wu H-JJ, Mauro D, Schett G, Ciccia F (2021) The gut–joint axis in rheumatoid arthritis. *Nat Rev Rheumatol* 17: 224 – 237

License: This is an open access article under the terms of the Creative Commons Attribution License, which permits use, distribution and reproduction in any medium, provided the original work is properly cited.

Expanded View Figures

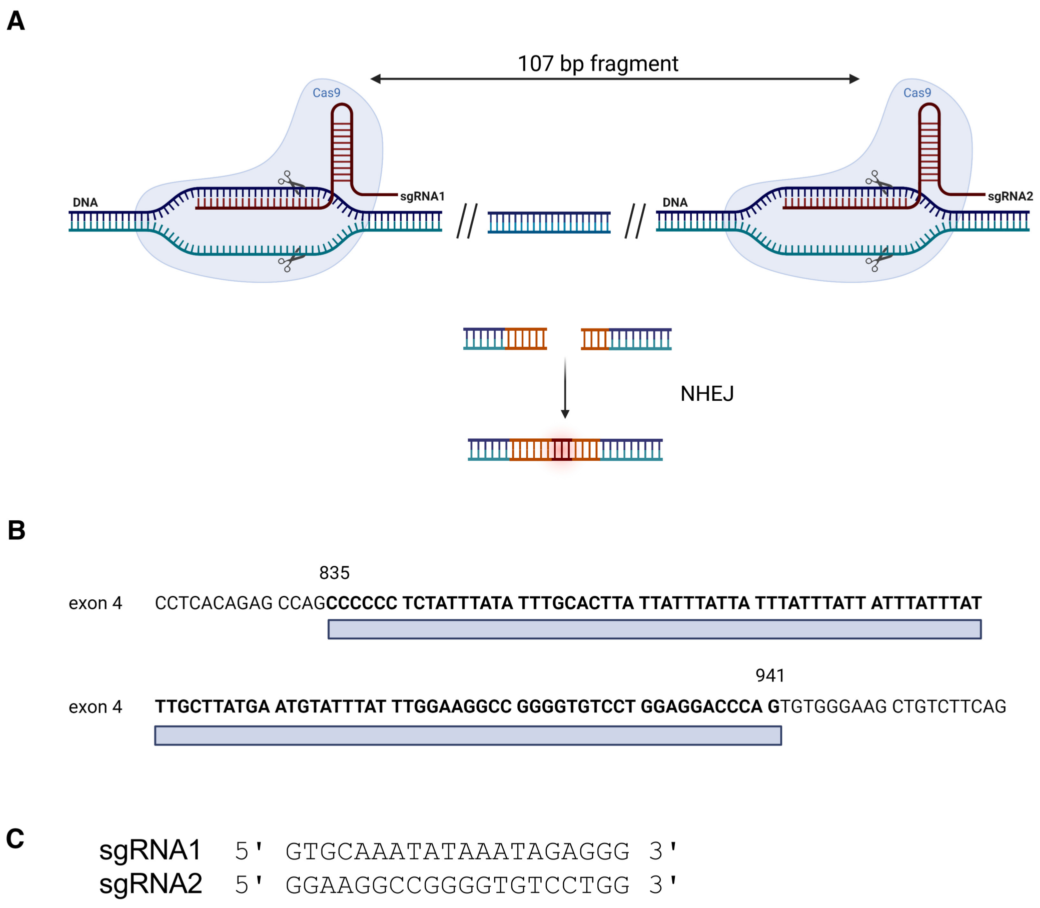

Figure EV1. Generation of a TNF-driven inflammation model by targeting the AU-rich element of the Tnf gene.

A Schematic overview of a deletion of a 107 bp fragment by CRISPR-Cas9 technology, followed by non-homologous end-joining (NHEJ).
B A 107-bp fragment was deleted in the 3′UTR region (exon 4) of the *Tnf* gene on chromosome 17, exon 4.
C Two guide RNA's were used to specifically delete the targeted region.

Data information: Figure created with Biorender.com.

© 2023 The Authors

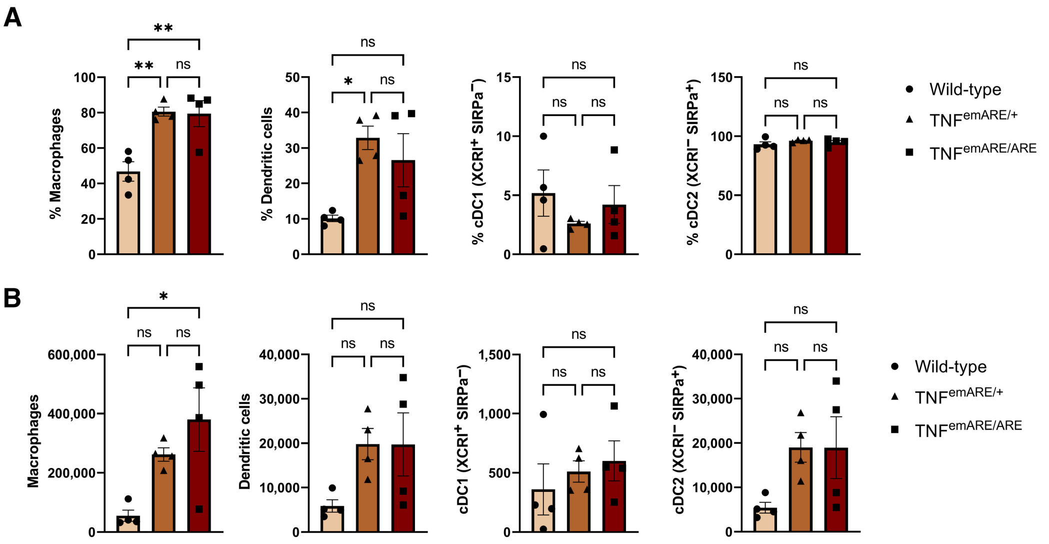

Figure EV2. Flow cytometry data of synovial leukocytes reveals a significant expansion of macrophages in TNF$^{emARE/+}$ and TNF$^{emARE/ARE}$ mice.

A Synovial flow cytometry data of 25 w/o TNF^{emARE} mice show an activated innate immune system (n = 8 mice/genotype, each datapoint represents data from two pooled mice).

B Absolute cell counts of synovial flow cytometry data (n = 8 mice/genotype, each datapoint represents data from two pooled mice).

Data information: Data are represented as Mean ± SEM, n = biological replicates with each datapoint on the graphs representing data of two pooled mice, one-way ANOVA test used with Tukey's multiple comparisons test. ns = P-value > 0.05, * = P-value ≤ 0.05, ** = P-value ≤ 0.01, *** = P-value ≤ 0.001, **** = P-value ≤ 0.0001.

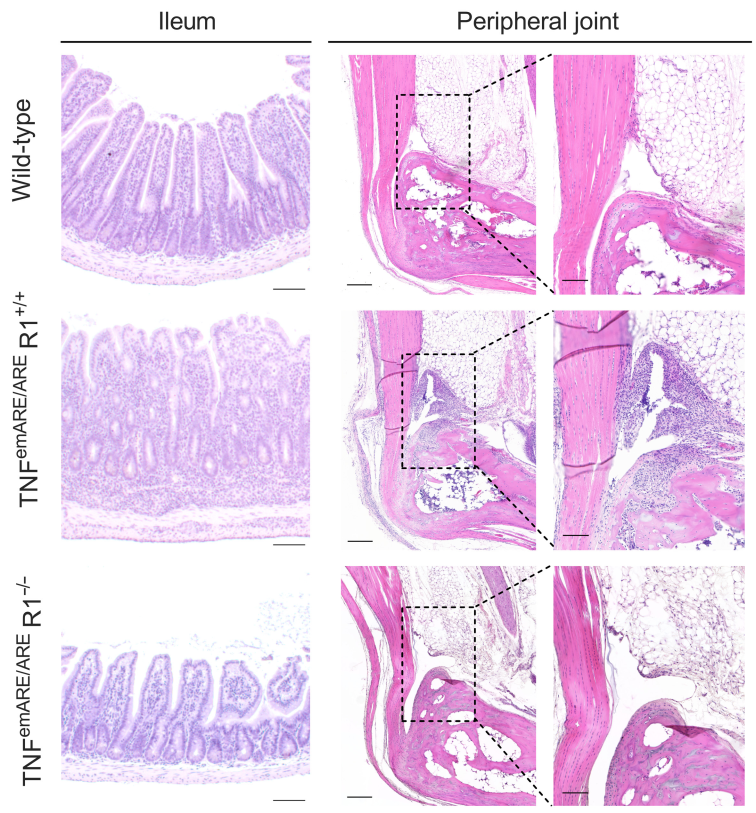

Figure EV3. TNF$^{emARE/ARE}$R1$^{-/-}$ mice are rescued from gut and joint pathology.

TNF$^{emARE/ARE}$R1$^{-/-}$ mice are rescued and do not display ileal pathology nor arthritis (Scale bars ileum: 100 μm, scale bars hind paws: 200 μm, scale bar focused images of SEC region: 100 μm).

© 2023 The Authors

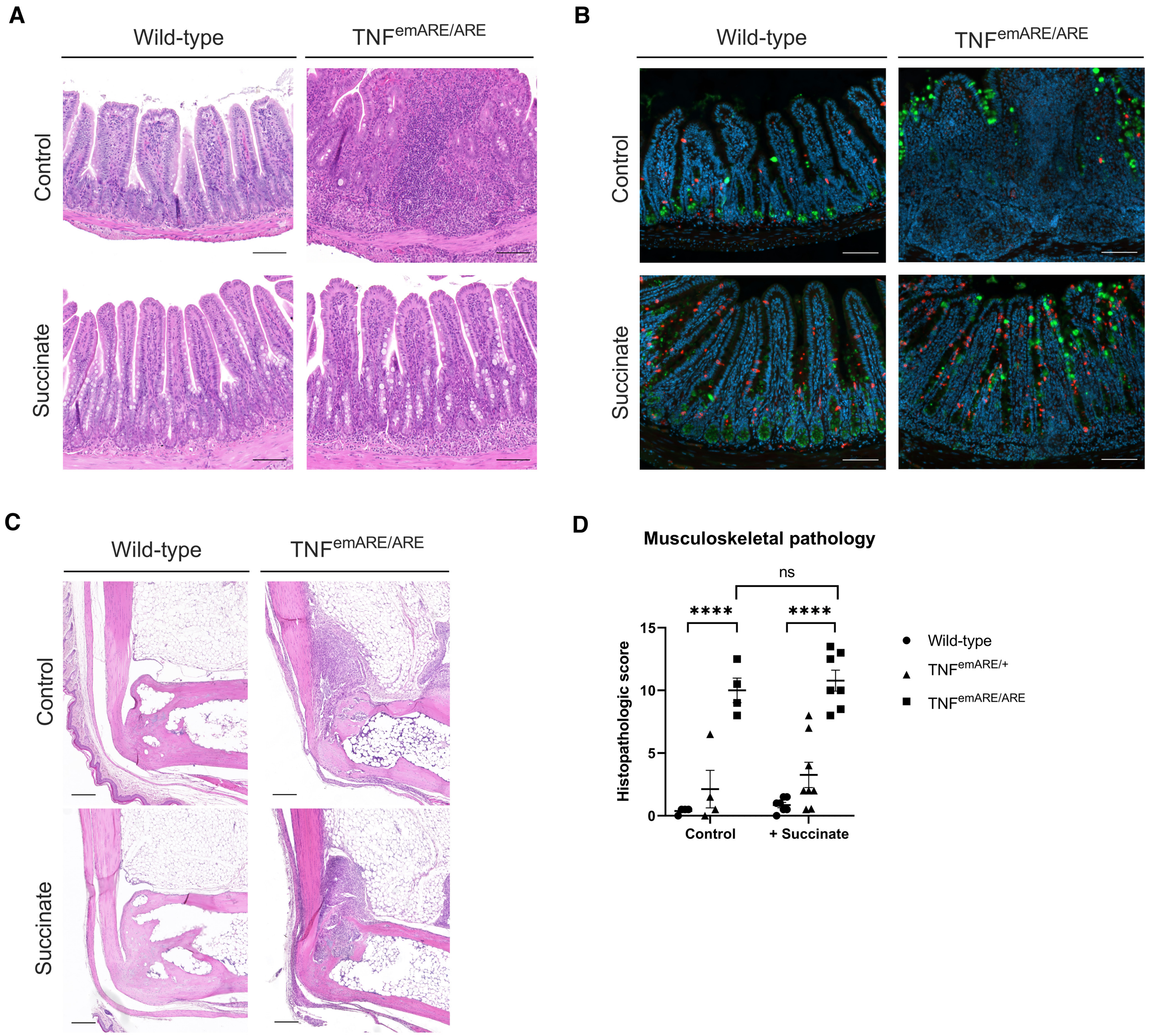

Figure EV4. Succinate supplementation induces tuft cell expansion and improvement of gut but not joint disease.

A Histological H&E sections of ileum indicate that succinate supplementation leads to improvement of ileal pathology (Scale bars: 100 μm).

B Immunofluorescent staining of $DCLK1^+$ cells (red) shows expansion of tuft cells in succinate-treated animals, in both wild-types and $TNF^{emARE/ARE}$ mice. Mucins = green (WGA + UEA-1), nuclei = blue (Hoechst; Scale bars: 100 μm).

C Succinate-treated mice are not rescued from arthritis development, disease severity is similar to control mice (Scale bars: 200 μm).

D Quantitative analysis of musculoskeletal pathology confirms no amelioration of joint disease in mice that received succinate treatment (Control groups n = 4/genotype; succinate-treated mice n = 8/genotype).

Data information: For (D), data are represented as Mean ± SEM, n = biological replicates, two-way ANOVA test used with Tukey's multiple comparisons test. ns = P-value > 0.05, * = P-value ≤ 0.05, ** = P-value ≤ 0.01, *** = P-value ≤ 0.001, **** = P-value ≤ 0.0001.

 © 2023 The Authors

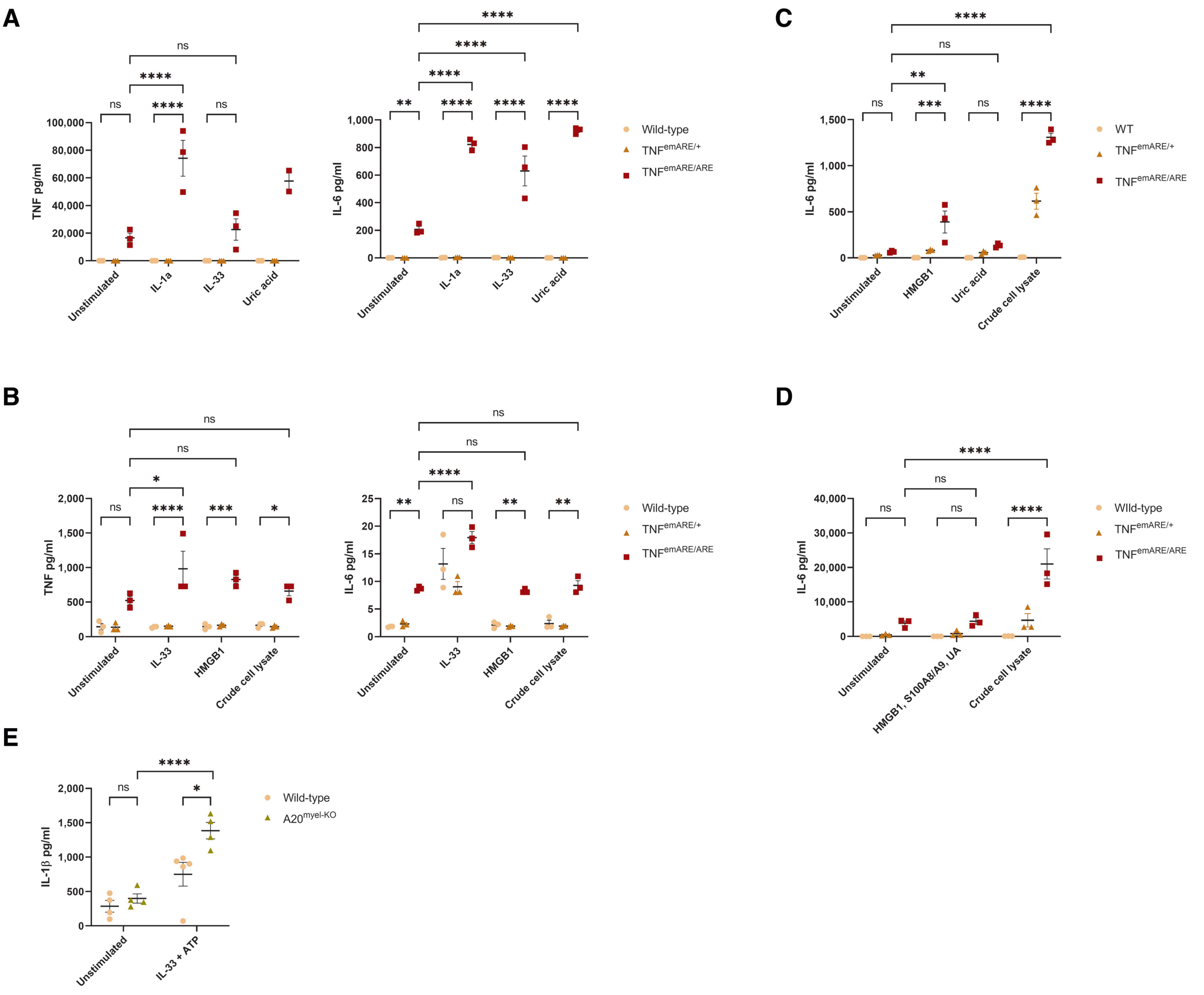

Figure EV5. BMDMs and SFs are hyperresponsive to stimulation with various DAMPs.

A Bio-plex cytokine assay for TNF and IL-6 on supernatant of BMDMs of 30–40 w/o TNF$^{emARE/ARE}$ mice after stimulation with various DAMPs (*n* = 3/genotype, except for uric acid stimulated TNF$^{emARE/ARE}$ cells *n* = 2).

B Bio-plex for TNF and IL-6 on supernatant of BMDMs of 7–9 w/o TNF$^{emARE/ARE}$ mice after stimulation with various DAMPs (*n* = 3/genotype).

C Bio-plex for IL-6 on supernatant of SFs of 30–40 w/o TNF$^{emARE/ARE}$ mice after stimulation with various DAMPs (*n* = 3/genotype).

D Bio-plex for IL-6 on supernatant of SFs of 7–9 w/o TNF$^{emARE/ARE}$ mice after stimulation with various DAMPs (*n* = 3/genotype).

E Bio-plex for IL-1beta on supernatant of BMDMs of 7–9 w/o A20$^{myel-KO}$ mice after stimulation with IL-33 + ATP (*n* = 4/genotype, except for IL-33 stimulated wild-type cells *n* = 5).

Data information: For (A, C), data are represented as Mean ± SEM, *n* = technical triplicates, two-way ANOVA test used with Tukey's multiple comparisons test. ns = *P*-value > 0.05, * = *P*-value ≤ 0.05, ** = *P*-value ≤ 0.01, *** = *P*-value ≤ 0.001, **** = *P*-value ≤ 0.0001. For (B, D, E), data are represented as Mean ± SEM, *n* = biological replicates, two-way ANOVA test used with Tukey's multiple comparisons test. ns = *P*-value > 0.05, * = *P*-value ≤ 0.05, ** = *P*-value ≤ 0.01, *** = *P*-value ≤ 0.001, **** = *P*-value ≤ 0.0001.

© 2023 The Authors

Appendix

Table of contents

- Appendix Figure S1: Phenotyping the TNF^{emARE} model: evaluating affected organs……1
- Appendix Figure S2: Gut phenotype of the TNF^{emARE} mouse model……………………………2
- Appendix Figure S3: Early onset of gut and joint disease in TNF$^{emARE/ARE}$ mice…………..3
- Appendix Figure S4: Absolute cell counts and gating strategy of small intestine flow cytometry…….4
- Appendix Figure S5: Phenotyping the A20$^{myel\text{-}KO}$ model: evaluating affected organs….6
- Appendix Table S1: Histopathological ileitis scoring…………………………………………………..7
- Appendix Table S2: Histopathological peripheral musculoskeletal disease scoring…….7
- Appendix Table S3: Histopathological axial musculoskeletal disease scoring…………….7

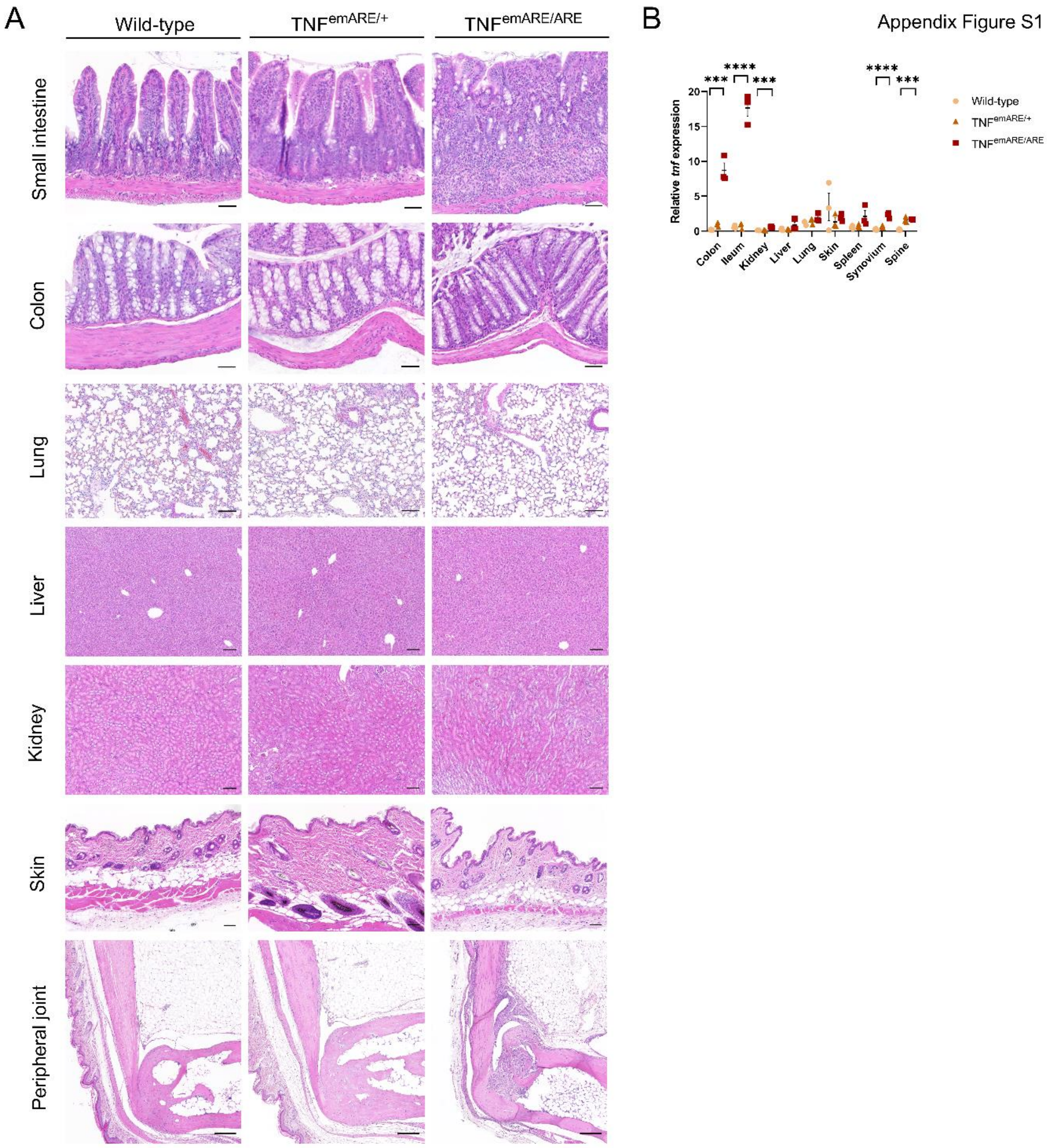

Figure S1: Phenotyping the TNF^{emARE} model: evaluating inflammation in multiple organs. (A) Histological evaluation of inflammation in sections of small intestine (ileum), colon, lung, liver, kidney, skin and peripheral joint (ankle) of wild-type, TNF$^{emARE/+}$ and TNF$^{emARE/ARE}$ mice (15-20 weeks old). (Scale bars colon, ileum, skin: 50 μm; kidney, liver, lung: 100 μm; ankle: 200 μm) (B) qPCR data on multiple tissues of TNF^{emARE} mice to evaluate expression of the *tnf* gene (15-20 weeks old mice) (n=3/genotype for every tissue).

Data information: For (B), data are represented as Mean +/- SEM, n=biological replicates, one-way ANOVA test used with Tukey's multiple comparisons test for every organ. ns= p-value > 0.05, * = p-value ≤ 0.05, ** = p-value ≤ 0.01, *** = p-value ≤ 0.001, **** = p-value ≤ 0.0001. Only significant differences between wild-types and homozygotes is shown.

Appendix Figure S2

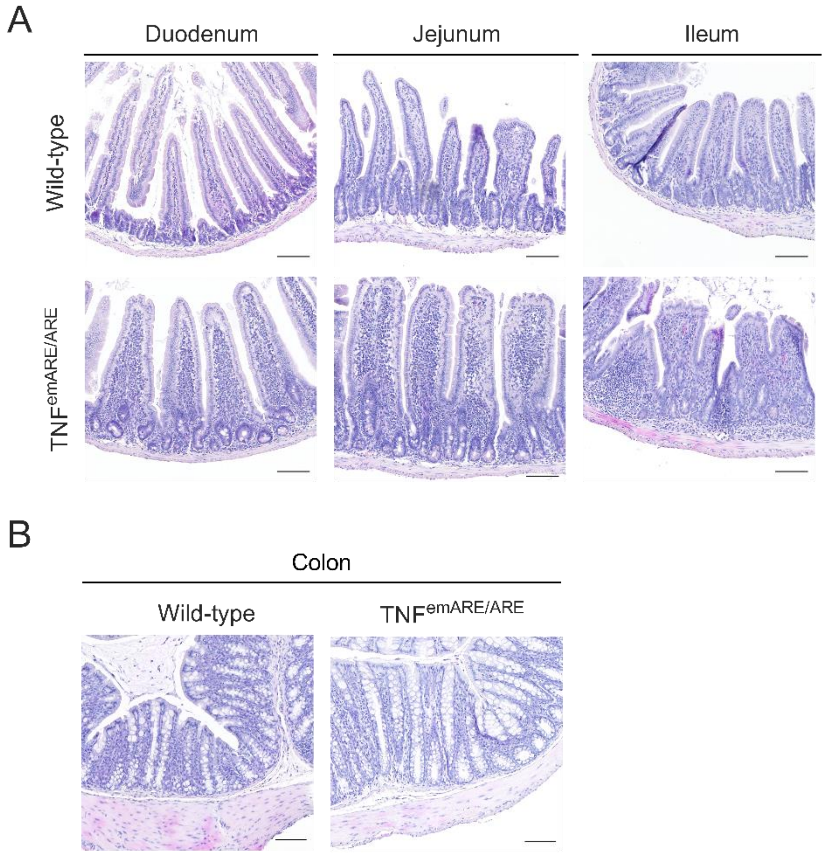

Figure S2: Gut phenotype of TNF^{emARE} mice. (A) Histological H&E stained sections of duodenum, jejunum, ileum and (B) colon of SPF wild-type and TNF$^{emARE/ARE}$ mice. (Scale bars: 100 µm). B) *il-1β* qPCR performed on multiple tissues in A20$^{myel-KO}$ and WT mice (20 weeks old) (n=3/genotype).

Data information: For (A, B), data are represented as Mean +/- SEM, n=biological replicates, one-way ANOVA test used with Tukey's multiple comparisons test for every organ. ns= p-value > 0.05, * = p-value ≤ 0.05, ** = p-value ≤ 0.01, *** = p-value ≤ 0.001, **** = p-value ≤ 0.0001.

Appendix Figure S3

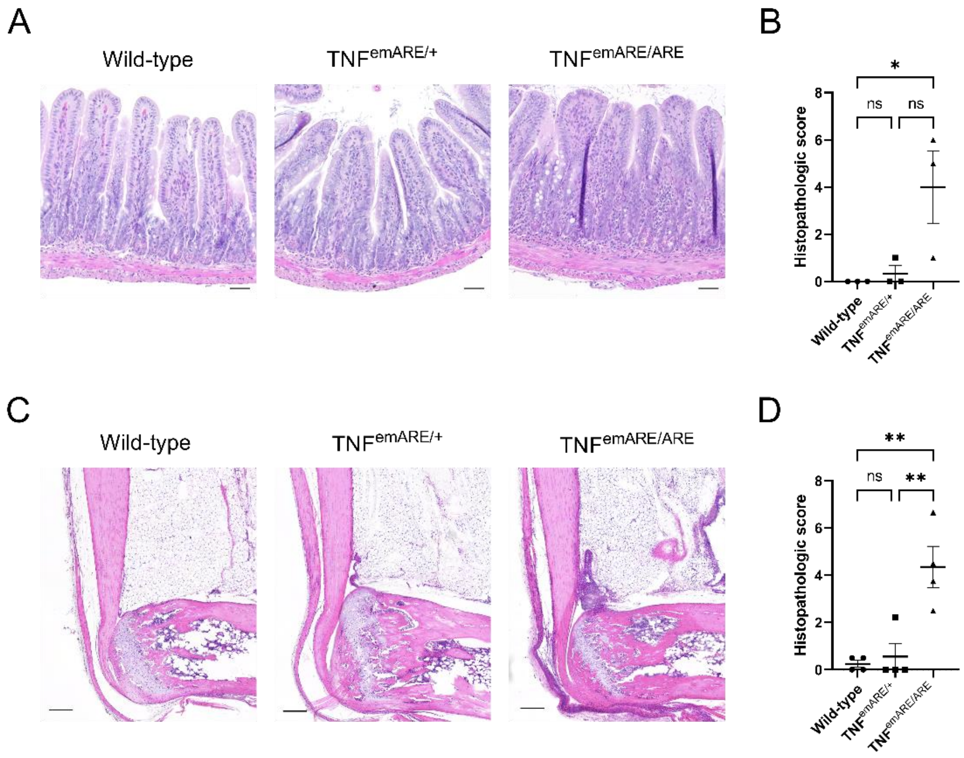

Figure S3: Early onset of gut and joint disease in TNF$^{emARE/ARE}$ mice. (A) Histological H&E stained ileal sections of 5-6 w/o SPF TNF^{emARE} mice. (Scale bars: 50µm) (B) Histopathological scoring for ileal disease in young (5-6 w/o) wild-type, TNF$^{emARE/+}$ and TNF$^{emARE/ARE}$ mice (n=3/genotype) (C) H&E stained ankle sections of young SPF TNF$^{emARE/ARE}$ mice (5-6 w/o). (Scale bars: 200µm) (D) Histopathologic scoring of musculoskeletal disease in 5-6 w/o wild-type (n=4), TNF$^{emARE/+}$ (n=4) and TNF$^{emARE/ARE}$ mice (n=4).

Data information: For graphs (B, D), data are represented as Mean +/- SEM, n=biological replicates. For graph (B), one-way ANOVA test was used with Tukey's multiple comparisons test. For graph (D), one-way ANOVA test was used with Holm-Šídák's multiple comparisons test. ns= p-value > 0.05, * = p-value ≤ 0.05, ** = p-value ≤ 0.01, *** = p-value ≤ 0.001, **** = p-value ≤ 0.0001.

Appendix Figure S4

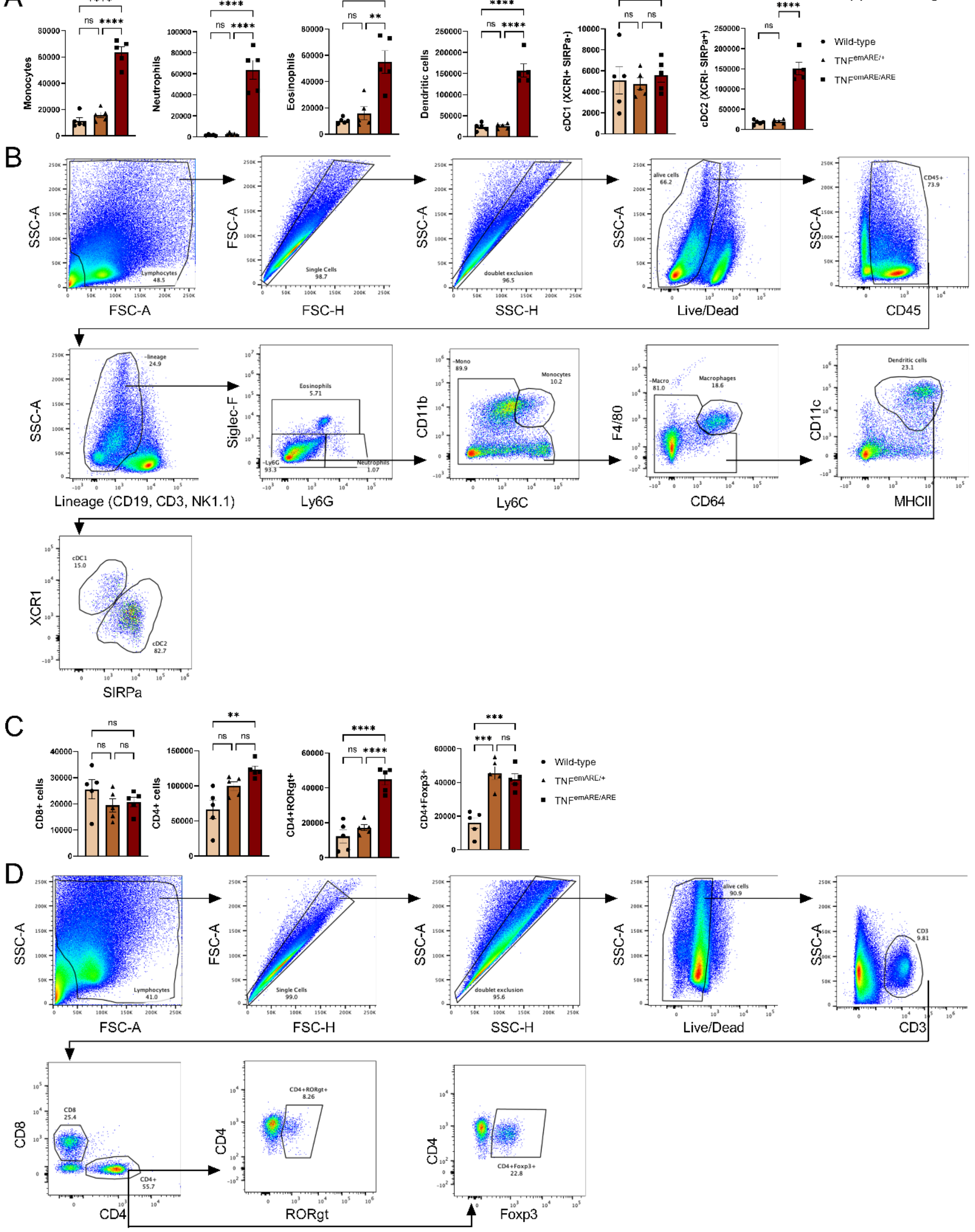

Figure S4: Absolute cell counts and gating strategy of small intestinal flow cytometry analysis. (A) Absolute cell counts of monocytes, neutrophils, eosinophils, dendritic cells, cDC1 and cDC2 cells in small intestinal lamina propria of wildtype, heterozygote and homozygote TNF^{emARE} mice. (n=5 mice/genotype) (B) Gating strategy of flow cytometry analysis of small intestinal lamina propria myeloid cells. (C) Absolute cell counts of CD8+, CD4+, Th17 and Treg cells in small intestinal lamina propria of wild-type, heterozygote and homozygote TNF^{emARE} mice. (n=5 mice/genotype) (D) Gating strategy of flow cytometry analysis of small intestinal lamina propria T cells.

Data information: For (A, C), data are represented as Mean +/- SEM, n=biological replicates, one-way ANOVA test used with Tukey's multiple comparisons test. ns= p-value > 0.05, * = p-value ≤ 0.05, ** = p-value ≤ 0.01, *** = p-value ≤ 0.001, **** = p-value ≤ 0.0001.

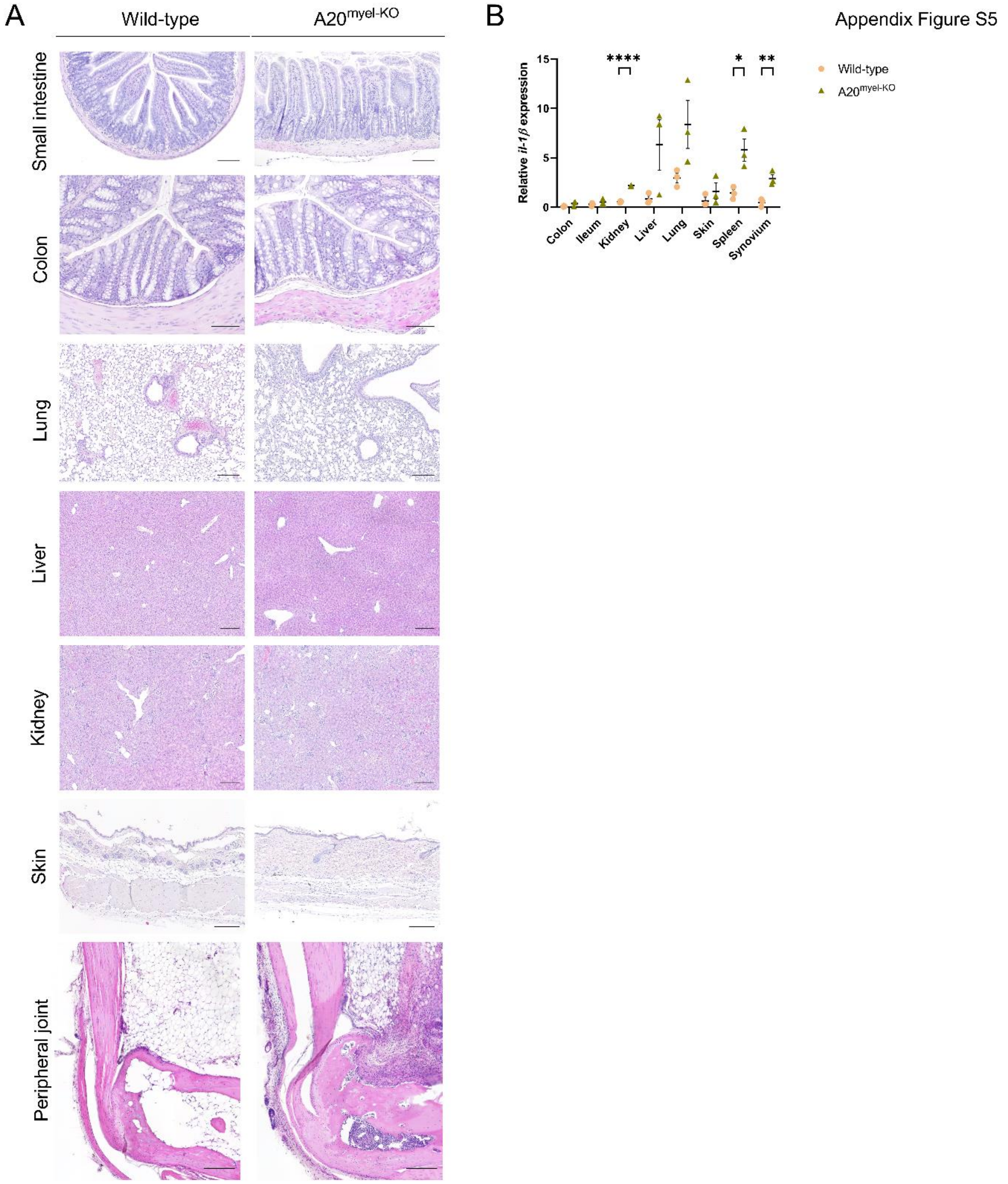

Figure S5: Phenotyping the A20myel-KO model: evaluating inflammation in multiple organs. Histological evaluation of inflammation in sections of small intestine, colon, lung, liver, kidney, skin and peripheral joint of wild-type versus A20myel-KO mice (20 weeks old). (Scale bars colon, ileum: 100 μm; kidney, liver, lung, skin, ankle: 200 μm). (B) qPCR data on multiple tissues of A20myel-KO mice to evaluate expression of the *il-1β* gene (20 weeks old mice) (n=3/genotype for every tissue).

Data information: For (B), data are represented as Mean +/- SEM, n=biological replicates, two-tailed unpaired t-test used for every organ. ns= p-value > 0.05, * = p-value ≤ 0.05, ** = p-value ≤ 0.01, *** = p-value ≤ 0.001, **** = p-value ≤ 0.0001.

Tables

Goblet cell count	- Normal, no GC loss (0)<br>- Minimal GC loss: loss of 1-30% (1)<br>- Clear GC loss: loss of 30-60% (2)<br>- Severe GC loss: loss of 60-90% (3)<br>- Maximal GC loss: loss of > 90% (4)
Immune cell infiltration	- Normal, no immune cell infiltration (0)<br>- Lamina propria immune cell infiltration (1)<br>- Mucosal infiltration: lamina propria and villi (2)<br>- Submucosal infiltration (3)<br>- Transmural infiltration (4)
Villus architecture	- Normal villus architecture (0)<br>- Minimal villus blunting (1)<br>- Blunted villi, only 50% of crypt remained (2)<br>- Blunted villi, only 25% of crypt remained (3)<br>- Maximal blunting, complete loss of villus-crypt architecture (4)

Appendix Table S1: Histopathological ileitis scoring

Immune cell infiltration	- immune cell infiltration in 0-15% of intervertebral discs or in the longitudinal ligament at the intervertebral disc level (0)<br>- immune cell infiltration in 15-50% of intervertebral discs or in the longitudinal ligament at the intervertebral disc level (1)<br>- immune cell infiltration in 50-90% of intervertebral discs or in the longitudinal ligament at the intervertebral disc level, or mild inflammation at the level of all discs (2)<br>- severe immune cell infiltration in all intervertebral discs/severe immune cell infiltration in the longitudinal ligament (3)

Appendix Table S2: Histopathological axial musculoskeletal disease scoring

Cuboidal joint:	- Immune infiltrates in synovium (0-2)<br>- Immune infiltrates in fat pad (0-2)<br>- Immune infiltrates in joint space (0-2)<br>- Immune infiltrates in cartilage (0-2)
Calcaneus:	- Bone erosion (0-2)<br>- Bone marrow edema (0-2)
Achilles tendon and Kargers' fat pad:	- Tendonitis (0-2)<br>- Immune infiltrates in synovium (0-2)<br>- Immune infiltrates in Kargers' fat pad (0-2)<br>- Immune infiltrates in joint space (0-2)

Appendix Table S3: Histopathological peripheral musculoskeletal disease scoring
