# Supplementary figures and images for: Sterile triggers drive joint inflammation in TNF‐ and IL‐1β‐dependent mouse arthritis models
Source: EMBO Mol Med. 2023 Sep 11;15(10):e17691. doi: 10.15252/emmm.202317691 (PMC10565626; doi:10.15252/emmm.202317691)

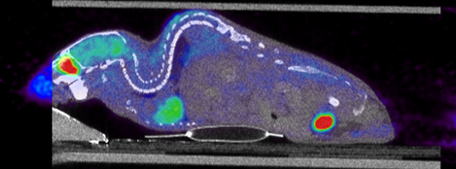

Supplement: Supplementary file 10 — Source Data for Figure 3 [file EMMM-15-e17691-s011.zip › Figure 3/3A/GF_ARE-ARE PETCT A.png]

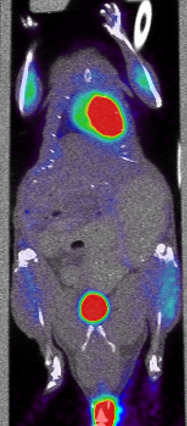

Supplement: Supplementary file 10 — Source Data for Figure 3 [file EMMM-15-e17691-s011.zip › Figure 3/3A/GF_ARE-ARE PETCT B.png]

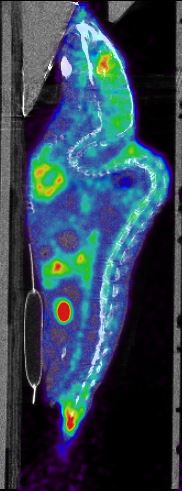

Supplement: Supplementary file 10 — Source Data for Figure 3 [file EMMM-15-e17691-s011.zip › Figure 3/3A/SPF_ARE-ARE_A.JPG]

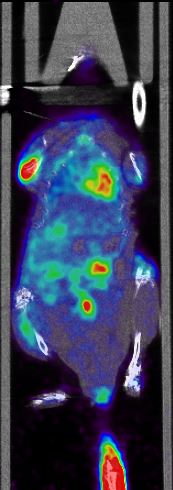

Supplement: Supplementary file 10 — Source Data for Figure 3 [file EMMM-15-e17691-s011.zip › Figure 3/3A/SPF_ARE-ARE_B.JPG]

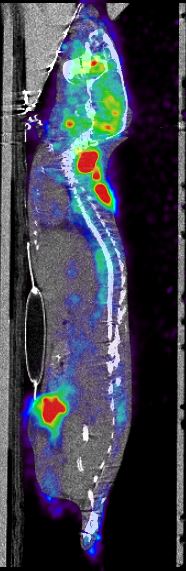

Supplement: Supplementary file 10 — Source Data for Figure 3 [file EMMM-15-e17691-s011.zip › Figure 3/3A/SPF_CONTROL_A.JPG]

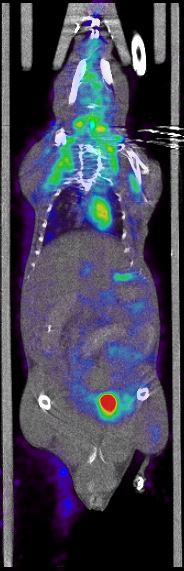

Supplement: Supplementary file 10 — Source Data for Figure 3 [file EMMM-15-e17691-s011.zip › Figure 3/3A/SPF_CONTROL_B.JPG]

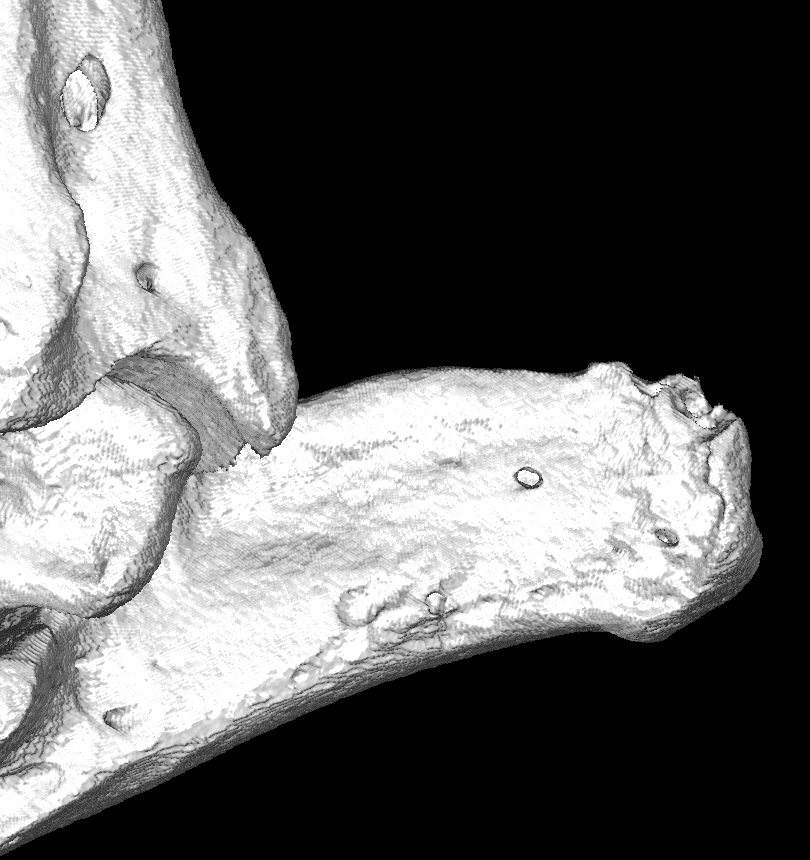

Supplement: Supplementary file 10 — Source Data for Figure 3 [file EMMM-15-e17691-s011.zip › Figure 3/3E/GF ARE-ARE_calcaneus2_croppedv.png]

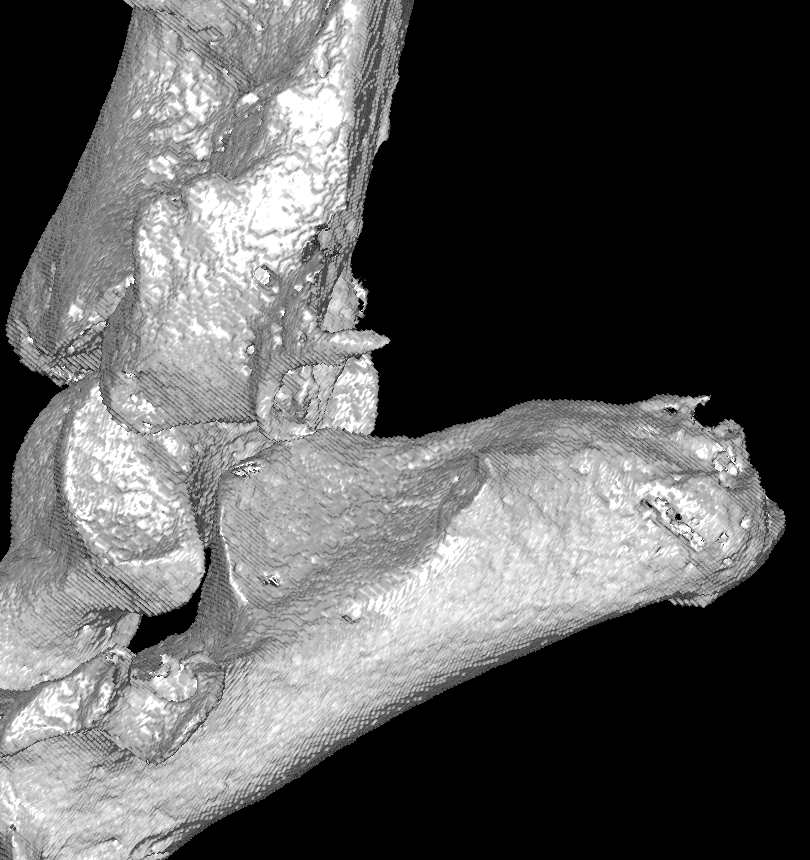

Supplement: Supplementary file 10 — Source Data for Figure 3 [file EMMM-15-e17691-s011.zip › Figure 3/3E/SPF ARE-ARE_calcaneus3_croppedv2.png]

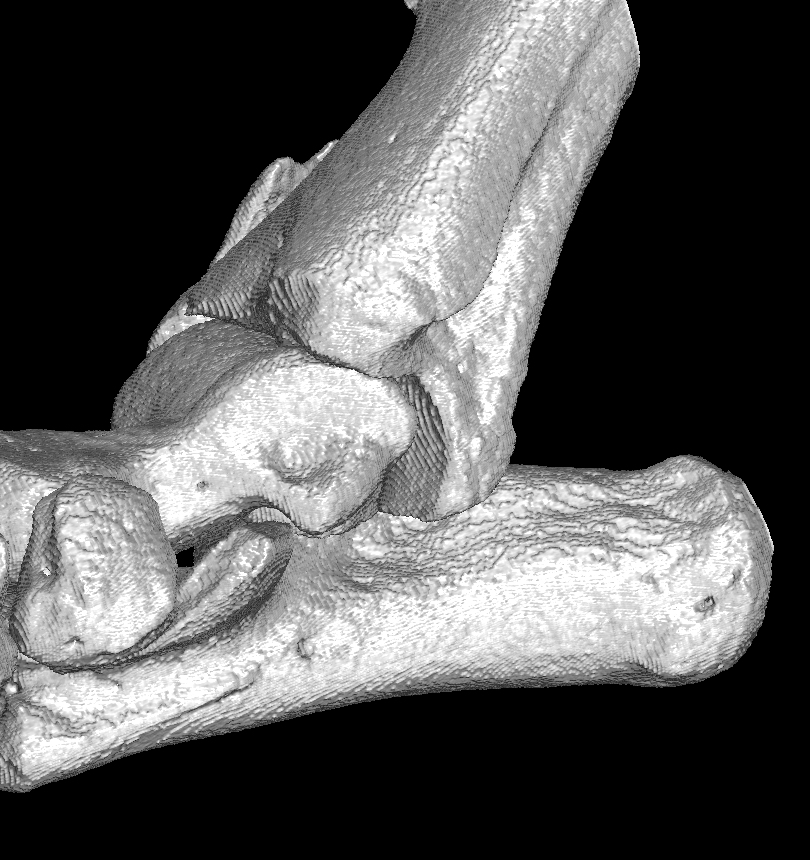

Supplement: Supplementary file 10 — Source Data for Figure 3 [file EMMM-15-e17691-s011.zip › Figure 3/3E/SPF WT_calcaneus4_cropped v2.png]

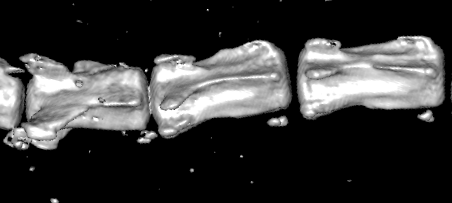

Supplement: Supplementary file 10 — Source Data for Figure 3 [file EMMM-15-e17691-s011.zip › Figure 3/3F/GF ARE-ARE_tail bridging_3vert.tif]

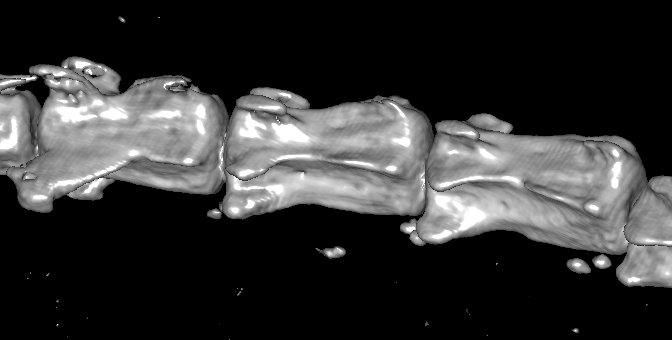

Supplement: Supplementary file 10 — Source Data for Figure 3 [file EMMM-15-e17691-s011.zip › Figure 3/3F/SPF ARE-ARE 739_tail bridging_3vert.tif]

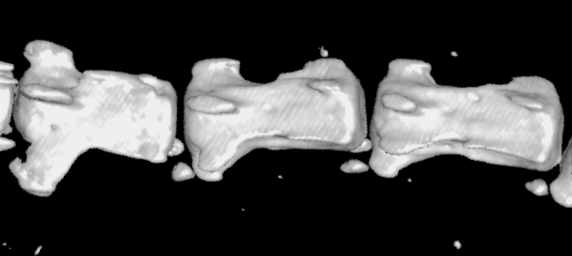

Supplement: Supplementary file 10 — Source Data for Figure 3 [file EMMM-15-e17691-s011.zip › Figure 3/3F/SPF WT_tail_3vert.tif]
