# Supplementary material for: Sterile triggers drive joint inflammation in TNF‐ and IL‐1β‐dependent mouse arthritis models
Source: EMBO Mol Med. 2023 Sep 11;15(10):e17691. doi: 10.15252/emmm.202317691 (PMC10565626; doi:10.15252/emmm.202317691)
Supplement: Supplementary file 13 [file EMMM-15-e17691-s004.pdf]

EMBO Molecular Medicine

Sterile triggers drive joint inflammation in TNF and IL-1β dependent mouse arthritis models

Alexandra Thiran, Ioanna Petta, Gillian Blancke, Marie Thorp, Guillaume Planckaert, Maude Jans, Vanessa Andries, Korneel Barbry, Elisabeth Gilis, Julie Coudenys, Tino Hochepied, Christian Vanhove, Eric Gracey, Emilie Dumas, Teddy Manuello, Ivan Josipovic, Geert van Loo, Dirk Elewaut, and Lars Vereecke
DOI: 10.15252/emmm.202317691

Corresponding author(s): Lars Vereecke (Lars.Vereecke@irc.vib-ugent.be)

Review Timeline:

Submission Date:	9th Mar 23
Editorial Decision:	6th Apr 23
Revision Received:	25th Jul 23
Editorial Decision:	15th Aug 23
Revision Received:	21st Aug 23
Accepted:	22nd Aug 23

Editor: Zeljko Durdevic

Transaction Report:

(Note: With the exception of the correction of typographical or spelling errors that could be a source of ambiguity, letters and reports are not edited. Depending on transfer agreements, referee reports obtained elsewhere may or may not be included in this compilation. Referee reports are anonymous unless the Referee chooses to sign their reports.)

6th Apr 2023

Dear Prof. Vereecke,

Thank you for the submission of your manuscript to EMBO Molecular Medicine. We have now received feedback from the three reviewers who agreed to evaluate your manuscript. As you will see from the reports below, all three referees recognize interest of the study but also raise serious concerns that should be addressed in a major revision of the current manuscript. Providing more mechanistic insight is of course welcomed but not essential for further consideration of the manuscript. If you would like to discuss further the points raised by the referees, I am available to do so via email or video. Let me know if you are interested in this option.

Further consideration of a revision that addresses reviewers' concerns in full will entail a second round of review. EMBO Molecular Medicine encourages a single round of revision only and therefore, acceptance or rejection of the manuscript will depend on the completeness of your responses included in the next, final version of the manuscript. For this reason, and to save you from any frustrations in the end, I would strongly advise against returning an incomplete revision.

We would welcome the submission of a revised version within three months for further consideration. Please let us know if you require longer to complete the revision.

Please use this link to login to the manuscript system and submit your revision: https://embomolmed.msubmit.net/cgi-bin/main.plex

I look forward to receiving your revised manuscript.

Yours sincerely,

Zeljko Durdevic

Zeljko Durdevic
Editor
EMBO Molecular Medicine

*****

When submitting your revised manuscript, please carefully review the instructions that follow below. We perform an initial quality control of all revised manuscripts before re-review; failure to include requested items will delay the evaluation of your revision.

We require:

1) A .docx formatted version of the manuscript text (including legends for main figures, EV figures and tables). Please make sure that the changes are highlighted to be clearly visible.

2) Individual production quality figure files as .eps, .tif, .jpg (one file per figure). For guidance, download the 'Figure Guide PDF': (https://www.embopress.org/page/journal/17574684/authorguide#figureformat).

3) A .docx formatted letter INCLUDING the reviewers' reports and your detailed point-by-point responses to their comments. As part of the EMBO Press transparent editorial process, the point-by-point response is part of the Review Process File (RPF), which will be published alongside your paper.

4) A complete author checklist, which you can download from our author guidelines (https://www.embopress.org/page/journal/17574684/authorguide#submissionofrevisions). Please insert information in the checklist that is also reflected in the manuscript. The completed author checklist will also be part of the RPF.

5) Please note that all corresponding authors are required to supply an ORCID ID for their name upon submission of a revised manuscript.

6) It is mandatory to include a 'Data Availability' section after the Materials and Methods. Before submitting your revision, primary datasets produced in this study need to be deposited in an appropriate public database, and the accession numbers and database listed under 'Data Availability'. Please remember to provide a reviewer password if the datasets are not yet public (see https://www.embopress.org/page/journal/17574684/authorguide#dataavailability).

In case you have no data that requires deposition in a public database, please state so in this section. Note that the Data Availability Section is restricted to new primary data that are part of this study.

7) For data quantification: please specify the name of the statistical test used to generate error bars and P values, the number (n) of independent experiments (specify technical or biological replicates) underlying each data point and the test used to calculate p-values in each figure legend. The figure legends should contain a basic description of n, P and the test applied. Graphs must include a description of the bars and the error bars (s.d., s.e.m.). See also 'Figure Legend' guidelines: https://www.embopress.org/page/journal/17574684/authorguide#figureformat

8) At EMBO Press we ask authors to provide source data for the main manuscript figures. Our source data coordinator will contact you to discuss which figure panels we would need source data for and will also provide you with helpful tips on how to upload and organize the files.

9) Our journal encourages inclusion of *data citations in the reference list* to directly cite datasets that were re-used and obtained from public databases. Data citations in the article text are distinct from normal bibliographical citations and should directly link to the database records from which the data can be accessed. In the main text, data citations are formatted as follows: "Data ref: Smith et al, 2001" or "Data ref: NCBI Sequence Read Archive PRJNA342805, 2017". In the Reference list, data citations must be labeled with "[DATASET]". A data reference must provide the database name, accession number/identifiers and a resolvable link to the landing page from which the data can be accessed at the end of the reference. Further instructions are available at .

10) We replaced Supplementary Information with Expanded View (EV) Figures and Tables that are collapsible/expandable online. A maximum of 5 EV Figures can be typeset. EV Figures should be cited as 'Figure EV1, Figure EV2" etc... in the text and their respective legends should be included in the main text after the legends of regular figures.

- For the figures that you do NOT wish to display as Expanded View figures, they should be bundled together with their legends in a single PDF file called *Appendix*, which should start with a short Table of Content. Appendix figures should be referred to in the main text as: "Appendix Figure S1, Appendix Figure S2" etc.

- Additional Tables/Datasets should be labeled and referred to as Table EV1, Dataset EV1, etc. Legends have to be provided in a separate tab in case of .xls files. Alternatively, the legend can be supplied as a separate text file (README) and zipped together with the Table/Dataset file.

See detailed instructions here:

.

11) The paper explained: EMBO Molecular Medicine articles are accompanied by a summary of the articles to emphasize the major findings in the paper and their medical implications for the non-specialist reader. Please provide a draft summary of your article highlighting

- the medical issue you are addressing,

- the results obtained and

- their clinical impact.

This may be edited to ensure that readers understand the significance and context of the research. Please refer to any of our published articles for an example.

12) For more information: There is space at the end of each article to list relevant web links for further consultation by our readers. Could you identify some relevant ones and provide such information as well? Some examples are patient associations, relevant databases, OMIM/proteins/genes links, author's websites, etc...

13) Author contributions: You will be asked to provide CRediT (Contributor Role Taxonomy) terms in the submission system. These replace a narrative author contribution section in the manuscript.

14) A Conflict of Interest statement should be provided in the main text.

15) Every published paper now includes a 'Synopsis' to further enhance discoverability. Synopses are displayed on the journal webpage and are freely accessible to all readers. They include a short stand first (maximum of 300 characters, including space) as well as 2-5 one-sentences bullet points that summarizes the paper. Please write the bullet points to summarize the key NEW findings. They should be designed to be complementary to the abstract - i.e. not repeat the same text. We encourage inclusion of key acronyms and quantitative information (maximum of 30 words / bullet point). Please use the passive voice. Please attach these in a separate file or send them by email, we will incorporate them accordingly.

Please also suggest a striking image or visual abstract to illustrate your article as a PNG file 550 px wide x 300-600 px high.

EMBO Molecular Medicine has a "scooping protection" policy, whereby similar findings that are published by others during review or revision are not a criterion for rejection. Should you decide to submit a revised version, I do ask that you get in touch after three months if you have not completed it, to update us on the status.

Please note: When submitting your revision you will be prompted to enter your funding and payment information. This will allow Wiley to send you a quote for the article processing charge (APC) in case of acceptance. This quote takes into account any reduction or fee waivers that you may be eligible for. Authors do not need to pay any fees before their manuscript is accepted and transferred to the publisher.

EMBO Press participates in many Publish and Read agreements that allow authors to publish Open Access with reduced/no publication charges. Check your eligibility: https://authorservices.wiley.com/author-resources/Journal-Authors/open-access/affiliation-policies-payments/index.html

***** Reviewer's comments *****

Referee #1 (Remarks for Author):

Previously, the conventional thought regarding SpA/arthritis development is considered as a microbiota-dependent immune cells-related pathology, also refers to as 'the gut-joint-axis'.

In this study, the authors for the first time provide the evidence that arthritis can be developed in germ-free conditions (microbiota-independent) by employing two transgenic mouse arthritis models,

The authors utilize a wide range of histological techniques to study the manifestations. Although these research data well support the intriguing conclusions, there are a few issues required to be addressed.

Major comments

The main issue remained to study here is the mechanism underlying how arthritis is developed independent of microbiota. In other words, what kind of 'joint-associated sterile factors' contribute to joint inflammation under germ free conditions?
As it comes to this issue, the authors simply mentioned DAMPs that might be the causal factors in the Discussion without further study in depth. The author should perform a series of studies to pinpoint which DAMP is attributed to joint inflammation under axenic conditions.

Minor comments

1, In terms of the histological staining data, as we can see in Fig. 1E, Fig. 2F (both upper and lower panel), Fig. 3B and 3D, Fig. 5C and 5E, sup Fig.2A and 2B, the scale bars are not equivalent in length from intra-group comparison,
Please explain the rationale behind these.

2, Supplementary Figure 2B should be annotated as 'Colon'.

3, In the document of Supplementary Figures, 'Supplementary Figure 2, Supplementary Figure 3, Supplementary Figure 4' should be indicated.

4, Figure S5A and S5B mentioned in the context line 209 were mislabeled. I suppose they should be referred to as Figure S4A and S4B.

5, There is a typo 'patters' in line 304.

Referee #2 (Remarks for Author):

In this manuscript, the authors have investigated the role of TNF-α in the gut-joint axis using a novel TNFemARE model, designed using CRISPR-Cas9 to create stablised Tnf mRNA, that shows severe intestinal and joint inflammation only in homozygous mice. The authors have used this model to investigate whether intestinal inflammation is critical for driving joint inflammation by housing mice in germ-free conditions, finding that when mice were housed in GF conditions, intestinal pathology was mitigated while joint pathology persisted.

The manuscript is written well overall, the findings are novel and offer insights into the importance of intestinal inflammation in arthritic diseases such as spondyloarthritis. The authors have drawn an overall balanced and proportional conclusion from the data.

Minor Comments:
1. Please provide more information on the relevance of TNF-α and IL-1β in spondyloarthritis, and how these models may aid in the understanding of these diseases. Serum IL-1β levels are generally not increased in people with axial spondyloarthritis. Please also specify which arthritic disease(s) IL-1β is thought to be important in (lines 232-234 & 236-237). Could you please include some discussion of increased intestinal permeability in healthy first-degree relatives of people with axial spondyloarthritis in either the discussion or the introduction?
See Bal et al., 2007 doi: https://doi.org/10.1007/s10067-006-0283-5; Rosine et al., 2018 doi: 10.1038/s41598-018-25722-z; Hecquet et al., 2021 doi: https://doi.org/10.1016/j.semarthrit.2021.04.015

2. In line 131, the conclusion that the TNFemARE mice show axial arthritis development is not supported by the data in the figure. Please include data that demonstrates axial arthritis in this model, or re-word this line.

3. In figures 1 & 2, serum TNFα was measured in each of the three groups of mice in SPF conditions (and in GF conditions in figure 2). Could you please explain the 10-fold discrepancy in concentration of serum TNFα between these two experiments?

4. In figure 3, it would be helpful to include thoracic spinal histology of TNFemARE/+ mice to support that there were no inflammatory infiltrates in this tissue. Please also include quantification of the thoracic spinal histology to support the argument that these mice develop axial arthritis.

5. Please explain the A20myel-KO model in more detail, include which cells/gene are targeted in this knock out. Please also include histology to confirm that there is no intestinal inflammation in this model in figure 5.

6. In the discussion (lines 272-274), please include other hypotheses about HLA-B27 involved in spondyloarthritis, or please highlight that the arthritogenic peptide is only one of the possible hypotheses.

7. I noticed that the ELISA incubation temperature is noted at 27 C, is this correct? And the antibody list in the flow cytometry method section seems to be missing the details for SIRPα, T-bet, and GATA3, along with some of the fluorophore information for some other antibodies?

8. Please include the gating strategy and graphs showing the numbers of each of the immune cell subsets for the flow cytometry experiment in the supplementary figures.

Referee #3 (Remarks for Author):

This article from Thiran et al is an important contribution to the 'gut-joint axis" field. Authors describe how germ-free conditions affect differently gut and joint inflammation in 2 novel models of arthritis. They describe that while gut inflammation is abolished by germ-free conditions, arthritis still occurs in both models.
Type 3 immune response (Th17, ILC3) develop and mature in contact with microbiota, how the authors discuss this in the context of GF mice developing arthritis which is known/believed mainly type 3 dependent ?
p10 l 306, can the authors provide references for these affirmations.
Age of SpA appearance in TNFemARE ? is Succinate experiment conclusive is enthesistis present before the age of 8 weeks ? this should be at least discussed.

While data appear convincing, no mechanistic explanations are offered.
Main concern:
- Is inflammation only present in joint ? Authors should observe other organs like skin, lungs, etc...
- Is the inflammation driven tissue specifically ? Can authors provide for instance RT-PCR measurement for TNF in the first model and for IL1b in the second models in joint, intestine, spleen, lungs and skin ?
- Which cells are expressing TNF in joint of the TNFemARE ?

- What are the molecular triggers of joint inflammation ? while I don't really understand the interest of scRNAseq in figure 1, it would have been very helpful on joint of TNFemARE in GF conditions...

Minor concerns

Please cite more recent clinical studies for IBD and SPA co-occurence.
Please discuss the term arthritis in the context of IBD
Please introduce more precisely the second model MyeloidA20KO,
and discuss the arthritis occuring in these mice (seems not enthesitis driven spA, more ressembling to animal model of RA)

Dear Editor,

We thank the Reviewers for their thorough evaluation of the manuscript, and for providing instructive and considerate comments. We are happy that Reviewers acknowledge the importance of our findings, but we also recognize that several issues needed to be addressed in the manuscript. We have put in a considerable effort to meet all of the points that were raised by the Reviewers by conducting novel experiments and by adapting the manuscript text, as appropriate. Together, we hope the revised manuscript will be positively evaluated by all Reviewers.

Below, we provide a 'point-by-point' response to Reviewer's comments:

Referee #1:

"Previously, the conventional thought regarding SpA/arthritis development is considered as a microbiota-dependent immune cells-related pathology, also refers to as 'the gut-joint-axis'. In this study, the authors for the first time provide the evidence that arthritis can be developed in germ-free conditions (microbiota-independent) by employing two transgenic mouse arthritis models, The authors utilize a wide range of histological techniques to study the manifestations. Although these research data well support the intriguing conclusions, there are a few issues required to be addressed. "

We thank the reviewer for appreciating the novelty of our study.

Major comments:

"The main issue remained to study here is the mechanism underlying how arthritis is developed independent of microbiota. In other words, what kind of 'joint-associated sterile factors' contribute to joint inflammation under germ free conditions? As it comes to this issue, the authors simply mentioned DAMPs that might be the causal factors in the Discussion without further study in depth. The author should perform a series of studies to pinpoint which DAMP is attributed to joint inflammation under axenic conditions."

We agree that it is important to identify DAMPs that induce sterile inflammation which contribute to arthritis development. Multiple intracellular DAMPs are known to activate pattern recognition receptors and drive tissue inflammation when released in conditions of cell damage, including HMGB1, S100A8/A9, IL1α, IL33, ATP, uric acid, Heat shock proteins, mitochondrial DNA, etc. In addition, extracellular matrix components can trigger immune activation and inflammation when released in damaged tissue, including fibronectin, tenacin-C, hyaluronan, etc. Given the wide diversity of intra- and extracellular DAMPs, we hypothesize that homeostatic DAMP concentrations are sufficient to drive inflammation in joints of genetically susceptible mice ($A20^{myel\text{-}KO}$ and TNF^{emARE}). Each DAMP can contribute to some extent to inflammation, or can act in synergy with other DAMPs, while it is currently unclear whether dominant DAMPs are responsible for arthritis development in $A20^{myel\text{-}KO}$ and TNF^{emARE} mice. Generating conditional gene knockouts of specific DAMPs in $A20^{myel\text{-}KO}$

and TNF^{emARE} background can provide more insights but we believe this was not feasible within a reasonable time frame. We hypothesize that multiple DAMPs may act synergistically to instigate arthritis in A20$^{myel\text{-}KO}$ and TNF^{emARE}, so knocking-out individual DAMPs in-vivo may not be sufficient to provide protection. Instead, we investigated whether sterile DAMPs can drive inflammatory responses in primary macrophages (bone marrow derived macrophages, BMDMs) or primary synovial fibroblasts (SFs) derived from A20$^{myel\text{-}KO}$ and TNF^{emARE} mice. We stimulated SF and BMDM cells with uric-acid (UA), HMGB1, IL-33, IL-1α, S100A8/A9 and with a crude mix of intracellular DAMPs derived from freeze-thawed cells (crude cell lysate). We performed Luminex Bioplex cytokine assays for TNF, IL-6 and IL-1β on cell supernatant of 48hours stimulated BMDM and SF cells and included the results in the revised manuscript (lines 348-379) and in an extra figure (Figure EV5):

"We investigated whether sterile DAMPs can drive inflammatory responses in primary macrophages (bone marrow-derived macrophages, BMDMs) or primary synovial fibroblasts (SFs) derived from A20$^{myel\text{-}KO}$ and TNF^{emARE} mice. We stimulated SF and BMDM cells with uric acid (UA), HMGB1, IL-33, IL-1α, S100A8/A9 or with a crude mix of intracellular DAMPs derived from freeze-thawed cells (crude cell lysate). We next performed Luminex Bio-plex cytokine assays on cell supernatant of cells after 48h of stimulation (Figure EV5). Fibroblasts and BMDMs derived from A20$^{myel\text{-}KO}$ and TNF^{emARE} had a baseline activation in unstimulated conditions, indicating that homeostatic inflammatory tone is already elevated in these primary cells. Stimulation of BMDMs derived from old (30-40w) TNF$^{emARE/ARE}$ mice with IL-1α induced significantly increased levels of TNF, compared to cells from wild-type and heterozygous littermate controls. IL-6 production in TNF$^{emARE/ARE}$ BMDMs was significantly higher after stimulation with IL-1 α, IL-33, and UA (Figure EV5 A). In BMDMs derived from young TNF$^{emARE/ARE}$ mice (7-9 weeks), both TNF and IL-6 secretion was increased upon stimulation with IL-33, HMGB1 and crude cell lysate (Figure EV5 B). Similarly, TNF$^{emARE/ARE}$ synovial fibroblasts secreted more IL-6 upon stimulation with crude cell lysate and HMGB1, compared to wildtype and TNF$^{emARE/+}$ control cells (Figure EV5 C,D). In A20$^{myel\text{-}KO}$ BMDMs, we found higher IL-1β secretion upon stimulation with IL-33 and ATP (Figure EV5 E). Together, we show that both macrophages and synovial fibroblasts derived from A20$^{myel\text{-}KO}$ and TNF^{emARE} mice secrete elevated levels of inflammatory cytokines in response to multiple DAMPs."

Minor comments

"1, In terms of the histological staining data, as we can see in Fig. 1E, Fig. 2F (both upper and lower panel), Fig. 3B and 3D, Fig. 5C and 5E, sup Fig.2A and 2B, the scale bars are not equivalent in length from intra-group comparison, Please explain the rationale behind these."

All scale bars are corrected and now have the same length.

"2, Supplementary Figure 2B should be annotated as 'Colon'."

Supplementary Figure 2B is now annotated as 'Colon'.

“3, In the document of Supplementary Figures, 'Supplementary Figure 2, Supplementary Figure 3, Supplementary Figure 4' should be indicated.”

Supplementary figure names are now indicated on the figures.

“4, Figure S5A and S5B mentioned in the context line 209 were mislabeled. I suppose they should be referred to as Figure S4A and S4B.”

Labeling is now corrected.

“5, There is a typo 'patters' in line 304.”

The type is corrected to ‘patterns’.

Referee #2:

“In this manuscript, the authors have investigated the role of TNF-α in the gut-joint axis using a novel TNF^{emARE} model, designed using CRISPR-Cas9 to create stablised Tnf mRNA, that shows severe intestinal and joint inflammation only in homozygous mice. The authors have used this model to investigate whether intestinal inflammation is critical for driving joint inflammation by housing mice in germ-free conditions, finding that when mice were housed in GF conditions, intestinal pathology was mitigated while joint pathology persisted.

The manuscript is written well overall, the findings are novel and offer insights into the importance of intestinal inflammation in arthritic diseases such as spondyloarthritis. The authors have drawn an overall balanced and proportional conclusion from the data.“

We thank the reviewer for acknowledging the novelty and significance of our study.

Minor Comments:

“1.a Please provide more information on the relevance of TNF-α and IL-1β in spondyloarthritis, and how these models may aid in the understanding of these diseases. Serum IL-1β levels are generally not increased in people with axial spondyloarthritis. Please also specify which arthritic disease(s) IL-1β is thought to be important in (lines 232-234 & 236-237). See Bal et al., 2007 doi: https://doi.org/10.1007/s10067-006-0283-5; Rosine et al., 2018 doi: 10.1038/s41598-018-25722-z;”

We have now included following text in the manuscript to clarify the importance of TNF and IL-1β in SpA:

TNF in SpA (lines 112-119): “TNF is a pivotal pro-inflammatory cytokine and therapeutic target in multiple inflammatory conditions such as IBD and SpA, and the response rate to anti-TNF therapy is approximately 60-70%. Interestingly, in very early peripheral SpA, treatment with anti-TNF agents has shown a remarkable ability to induce sustained clinical

drug-free remission, indicating the significance of an early intervention window that maximizes the response to TNF inhibition (Carron *et al.*, 2017). Despite its widespread use in clinical practice, TNF inhibition is associated with a notable proportion of non-responders and an increased risk of infection."

Relevance of mouse models (Lines 424-427): "The TNF^{emARE} and $A20^{myel\text{-}KO}$ are valuable models to unravel the downstream pathogenic mechanisms which are induced by elevated TNF and IL-1β levels respectively, but also to identify the critical upstream triggers for TNF and IL-1β induction in various tissue compartments "

IL1β in SpA (lines 297-302):" Inflammasome activation has been observed in both preclinical models and in patients with SpA, which was shown to drive type-3 cytokine production in an IL-1β–dependent mechanism, and found to be associated with intestinal dysbiosis (Guggino *et al.*, 2021). Anakinra, a human interleukin-1 receptor antagonist, is currently used to treat patients suffering from various inflammasomopathies, including crystal-induced arthropathies such as gout (Malcova *et al.*, 2021)"

"1.b Could you please include some discussion of increased intestinal permeability in healthy first-degree relatives of people with axial spondyloarthritis in either the discussion or the introduction? Hecquet et al., 2021 doi: https://doi.org/10.1016/j.semarthrit.2021.04.015 "

We added following text related to intestinal permeability in lines 387-392 and added the reference Hecquet et al., 2021 as suggested:
"Clinical observations in human IBD and arthritis suggest a common disease pathophysiology, commonly termed the 'gut-joint' axis. The fact that intestinal inflammation, though often subclinical, or an enteric infection, precedes joint inflammation in some patients, may support a causal relationship. Both gut and joint inflammation are associated with intestinal barrier permeability, which is even apparent in non-symptomatic first degree relatives of SpA patients (Hecquet *et al.*, 2021)."

"2. In line 131, the conclusion that the TNFemARE mice show axial arthritis development is not supported by the data in the figure. Please include data that demonstrates axial arthritis in this model, or re-word this line. "

Axial arthritis is present, as shown in figure 3 (SPF versus GF comparison). As we do not show axial arthritis in Figure 1, we rephrased the sentence.

"3. In figures 1 & 2, serum TNFα was measured in each of the three groups of mice in SPF conditions (and in GF conditions in figure 2). Could you please explain the 10-fold discrepancy in concentration of serum TNFα between these two experiments? "

We repeated the ELISA assay (Figure 1), re-analyzed and adjusted the graphs according to the new data. In Figure 1, serum was provided from mice of 35 weeks old, while in Figure 2, serum of 25 weeks old mice was used. In Figure 1, levels of serum TNF are overall higher compared to the serum levels in Figure 2, what we attribute to the age difference; older mice (with a more severe disease phenotype) have higher serum TNF levels compared to

younger mice. Besides this, experimental variation between ELISA experiments can occur (caused by storage-time of samples, number of freeze/thaw cycles, standard quality,…etc), so accurate comparisons can only be made between experimental groups in the same experiment.

"4. In figure 3, it would be helpful to include thoracic spinal histology of TNFemARE/+ mice to support that there were no inflammatory infiltrates in this tissue. Please also include quantification of the thoracic spinal histology to support the argument that these mice develop axial arthritis. "

We included a representative picture of $TNF^{emARE/+}$ spine, and also quantified immune cell infiltration along the spinal longitudinal ligament (Figure 3B, Figure 3D)

"5. Please explain the A20myel-KO model in more detail, include which cells/gene are targeted in this knock out. Please also include histology to confirm that there is no intestinal inflammation in this model in figure 5."

The $A20^{myel\text{-}KO}$ model was clarified and included in lines 306-3012:
"$A20^{myel\text{-}KO}$ mice, generated by crossing floxed-*A20/Tnfaip3* mice with Lysozyme-Cre transgenic mice, which leads to conditional *A20* deletion in the myeloid compartment, were previously shown to develop an erosive TNF-independent but NLRP3 inflammasome- and IL-1β–dependent polyarthritis resembling rheumatoid arthritis. In this model, arthritis develops as a result of macrophage necroptosis, which leads to inflammasome activation and the release of IL-1β and intracellular danger-associated molecular patterns (DAMPs) (Matmati *et al.*, 2011; Walle *et al.*, 2014; Polykratis *et al.*, 2019) "

Histological sections of colon and small intestine of 20 weeks-old $A20^{myel\text{-}KO}$ mice were included in Appendix Figure S5 to confirm there is no intestinal inflammation in these mice.

"6. In the discussion (lines 272-274), please include other hypotheses about HLA-B27 involved in spondyloarthritis, or please highlight that the arthritogenic peptide is only one of the possible hypotheses. "

The hypotheses about HLA-B27 in SpA were expanded as follows (lines 394-398): "The strongest genetic risk factor for SpA in humans is the human major histocompatibility complex (*MHC*) class I allele *HLA-B27*, which is believed to contribute to arthritis by promoting intestinal dysbiosis by driving endoplasmic reticulum stress and activation of the unfolded protein response upon HLA-B27 misfolding, and by activating CD8+ cytotoxic T cells upon recognition of arthritogenic peptides (Dumas *et al.*, 2020; Kavadichanda *et al.*, 2021)"

"7. I noticed that the ELISA incubation temperature is noted at 27℃, is this correct? And the antibody list in the flow cytometry method section seems to be missing the details for SIRPα, T-bet, and GATA3, along with some of the fluorophore information for some other antibodies?"

The ELISA incubation temperature is 27°C according to our protocol. The antibody list in the flow cytometry method section is now updated and includes details on all antibodies used.

"8. Please include the gating strategy and graphs showing the numbers of each of the immune cell subsets for the flow cytometry experiment in the supplementary figures. "

We included the gating strategy and graphs of individual immune cell subsets in Appendix figure S4

Referee #3:

"This article from Thiran et al is an important contribution to the 'gut-joint axis" field. Authors describe how germ-free conditions affect differently gut and joint inflammation in 2 novel models of arthritis. They describe that while gut inflammation is abolished by germ-free conditions, arthritis still occurs in both models. "

We thank the reviewer for evaluating our study as an important contribution to the field.

"1. Type 3 immune response (Th17, ILC3) develop and mature in contact with microbiota, how the authors discuss this in the context of GF mice developing arthritis which is known/believed mainly type 3 dependent?"

This is a very valid remark. The microbiota is very important for immune maturation, and especially for activation of type-3 immunity, including Th17 and ILC3 cells. Type-3 immunity is known to contribute to arthritis development in various preclinical mouse models, including the K/BxN and SKG mouse arthritis models. The K/BxN autoimmune arthritis model is dependent on the microbiota, and particularly on segmented filamentous bacteria (SFB), for the autoimmune phenotype (Block et al., J. Immunol., 2016). Germ-free SKG mice develop arthritis upon curdlan injection, but to a milder degree compared to SPF raised SKG mice (Rehaume et al., Arthritis & Rheumatology 2014). However, we show that both TNF^{emARE} and $A20^{myel\text{-}KO}$ mice develop full-blown arthritis in germ-free conditions, indicating that these models are not affected by the premature type-3 immune arm, and thus probably develop independent of type-3 immunity. We hypothesize that also in humans, type-3 immunity contributes to a varying degree to joint pathology. This can explain why certain patients benefit from anti-TNF therapy, while other benefit better from anti-IL-17 or anti-IL-23 therapies. Therefore, we believe the microbiota could play a more significant role in strong type-3 driven arthritic disease, but not necessarily in strong innate cytokine (TNF & IL-1β) driven disease.

We modified the in line 405-411: "Other experimental mouse arthritis models, including K/BxN, CIA and SKG require microbial triggers to drive type-3 immunity and arthritis development (Sakaguchi *et al.*, 2003; Rehaume *et al.*, 2014; Liu *et al.*, 2016; Teng *et al.*, 2017; Jubair *et al.*, 2018). We therefore hypothesize that patients with genetic defects in type-3 immune-associated genes may be more prone to microbial-induced inflammatory pathology, including arthritis. In contrast, inflammatory arthritis caused by increased levels of or response to TNF and IL1-⍰ are less likely to be driven or influenced by microbial triggers "

"2. p10 l 306, can the authors provide references for these affirmations. "

References are now added to the statements in line 431-434.

"3. Age of SpA appearance in TNFemARE ? Is enthesistis present before the age of 8 weeks ?"

We observe arthritis development in homozygous TNF^{emARE} mice from the age of 5-6 weeks. We included histological evidence in Appendix Figure S3.

"4. is Succinate experiment conclusive ?"

The rationale behind the succinate experiment was to evaluate whether a microbial metabolite previously shown to improve TNF-driven gut inflammation, also improved joint inflammation. Our data however show no clinical arthritis improvement in response to succinate, and thereby further emphasize the functional disconnection between TNF driven gut & joint pathology.

"5. While data appear convincing, no mechanistic explanations are offered."
Main concern:
- Is inflammation only present in joint ? Authors should observe other organs like ***skin, lungs****, etc..."*

We evaluated inflammation in other organs than gut & joint, and provided histological sections of liver, kidney, skin and lungs of TNF^{emARE} and $A20^{myel\text{-}KO}$ mice (Appendix Figure S1 and S5). We observe no clear signs of inflammation in liver, kidney, skin, gut and lung sections of 20 weeks old $A20^{myel\text{-}KO}$ mice. In 15 weeks old TNF^{emARE} mice, we observe no inflammation in liver, kidney, skin and lung sections.

"6. - Is the inflammation driven tissue specifically ? Can authors provide for instance RT-PCR measurement for TNF in the first model and for IL1b in the second models in joint (ankle/Knee/spines), intestine (SI/Colon), spleen, lungs and skin ?"

We performed RT-qPCR on colon, ileum, kidney, liver, lung, spleen, skin, knee synovium and spine for *Tnf* in TNF^{emARE} and for *Il-1*β in $A20^{myel\text{-}KO}$ mice. We observed an upregulation of *Tnf* expression in ileum, colon, kidney, knee synovium and spine in $TNF^{emARE/ARE}$ mice compared to wild-type and heterozygote $TNF^{emARE/+}$ mice, though highest TNF levels are found in ileum. In $A20^{myel\text{-}KO}$ mice, we see a significant increase of *Il-1*β expression in spleen, kidney and knee synovium, not in colon, ileum, ,liver, lung and skin. These data are included in extra data figures: Appendix Figure S1 and Appendix Figure S5.

Figure 2: (A)

"7. - Which cells are expressing TNF in joint of the TNFemARE ? "

Various cell types can produce TNF and influence pathology in the joints, including myeloid cells, T cells, B-cells and fibroblasts. A recent study examined the contribution of various TNF-producing cell types using conditional TNF knockout mice (T, B and Myeloid cells) in the collagen-induced arthritis model. Myeloid -cell derived TNF was found to contribute to both induction and progression of arthritis, while T-cell derived TNF was found protective in the induction phase. B-cell derived TNF contributes to arthritis by influencing autoantibody production (Kruglov et al;, Ann Rheum Dis., 2020). In TNFdARE mice, mesenchymal cells were identified as most critical TNF-responding cells during the development of both gut and joint inflammation (Armaka et al., J Exp Med., 2008). In TNFemARE mice, we observe increased baseline TNF expression in both macrophages and synovial fibroblasts (EV Figure 5).

"8. - What are the molecular triggers of joint inflammation ? "

This remark was also made by Referee #1, we provide the same response:

We agree that it is important to identify DAMPs that induce sterile inflammation which contribute to arthritis development. Multiple intracellular DAMPs are known to activate pattern recognition receptors and drive tissue inflammation when released in conditions of cell damage, including HMGB1, S100A8/A9, IL1α, IL33, ATP, uric acid, Heat shock proteins, mitochondrial DNA, etc. In addition, extracellular matrix components can trigger immune activation and inflammation when released in damaged tissue, including fibronectin, tenacin-C, hyaluronan, etc. Given the wide diversity of intra- and extracellular DAMPs, we hypothesize that homeostatic DAMP concentrations are sufficient to drive inflammation in joints of genetically susceptible mice (A20$^{myel-KO}$ and TNF^{emARE}). Each DAMP can contribute to some extent to inflammation, or can act in synergy with other DAMPs, while it is currently unclear whether dominant DAMPs are responsible for arthritis development in A20$^{myel-KO}$ and TNF^{emARE} mice. Generating conditional gene knockouts of specific DAMPs in A20$^{myel-KO}$ and TNF^{emARE} background can provide more insights but we believe this was not feasible within a reasonable time frame. We hypothesize that multiple DAMPs may act synergistically to instigate arthritis in A20$^{myel-KO}$ and TNF^{emARE}, so knocking-out individual DAMPs in-vivo may not be sufficient to provide protection. Instead, we investigated whether sterile DAMPs can drive inflammatory responses in primary macrophages (bone marrow derived macrophages, BMDMs) or primary synovial fibroblasts (SFs) derived from A20$^{myel-KO}$ and TNF^{emARE} mice. We stimulated SF and BMDM cells with uric-acid (UA), HMGB1, IL-33, IL-1α, S100A8/A9 and with a crude mix of intracellular DAMPs derived from freeze-thawed cells (crude cell lysate). We performed Luminex Bioplex cytokine assays for TNF, IL-6 and IL-1β on cell supernatant of 48hours stimulated BMDM and SF cells and included the results in the revised manuscript (lines 348-379) and in an extra figure (Figure EV5):

"We investigated whether sterile DAMPs can drive inflammatory responses in primary macrophages (bone marrow-derived macrophages, BMDMs) or primary synovial fibroblasts (SFs) derived from A20$^{myel-KO}$ and TNF^{emARE} mice. We stimulated SF and BMDM cells with uric acid (UA), HMGB1, IL-33, IL-1α, S100A8/A9 or with a crude mix of intracellular DAMPs derived from freeze-thawed cells (crude cell lysate). We next performed Luminex Bio-plex cytokine assays on cell supernatant of cells after 48h of stimulation (Figure EV5). Fibroblasts and BMDMs derived from A20$^{myel-KO}$ and TNF^{emARE} had a baseline activation in unstimulated

conditions, indicating that homeostatic inflammatory tone is already elevated in these primary cells. Stimulation of BMDMs derived from old (30-40w) TNF$^{emARE/ARE}$ mice with IL-1α induced significantly increased levels of TNF, compared to cells from wild-type and heterozygous littermate controls. IL-6 production in TNF$^{emARE/ARE}$ BMDMs was significantly higher after stimulation with IL-1 α, IL-33, and UA (Figure EV5 A). In BMDMs derived from young TNF$^{emARE/ARE}$ mice (7-9 weeks), both TNF and IL-6 secretion was increased upon stimulation with IL-33, HMGB1 and crude cell lysate (Figure EV5 B). Similarly, TNF$^{emARE/ARE}$ synovial fibroblasts secreted more IL-6 upon stimulation with crude cell lysate and HMGB1, compared to wildtype and TNF$^{emARE/+}$ control cells (Figure EV5 C,D). In A20$^{myel-KO}$ BMDMs, we found higher IL-1β secretion upon stimulation with IL-33 and ATP (Figure EV5 E). Together, we show that both macrophages and synovial fibroblasts derived from A20$^{myel-KO}$ and TNF^{emARE} mice secrete elevated levels of inflammatory cytokines in response to multiple DAMPs."

"9. while I don't really understand the interest of scRNAseq in figure 1, it would have been very helpful on joint of TNFemARE in GF conditions... "

There is no scRNAseq data in Figure 1, the t-sne plots (Fig1G, I) represent concatenated flow-cytometry data of ileal myeloid and T cell subsets, respectively. We now provide flow-cytometry data on synovial cells from WT and TNF^{emARE} SPF mice (Figure EV2). We observe an increase of macrophages and dendritic cells, while for the other cell populations, no significant changes could be observed. Since flow-cytometry analyses require large groups of mice, and GF TNF^{emARE} mice are only available at low numbers, we were not able to replicate these analyses on GF TNF^{emARE} mice. However, since histopathology in SPF and GF TNF^{emARE} mice is very similar, we expect similar immune cell profiles in synovia from GF TNF^{emARE} mice.

Minor concerns

"10. Please cite more recent clinical studies for IBD and SPA co-occurence. "

More recent reference was added (line 103): Kopylov *et al.*, 2018

"11. Please discuss the term arthritis in the context of IBD "

We included information about SpA occurrence in the context of IBD in lines (103-109): "Furthermore, SpA emerges as the most prevalent extra-intestinal manifestation observed in IBD patients. The reported prevalence rates of SpA in individuals with IBD range between 6 and 46% (Brakenhoff *et al.*, 2010; Ossum *et al.*, 2018). Both axial and peripheral involvements have been reported, with peripheral SpA often coinciding with relapses of intestinal disease, while axial SpA appear to progress independently of the severity and activity of IBD (Sheth, Pitchumoni and Das, 2015; Rogler *et al.*, 2021; Barkhodari *et al.*, 2022)."

"12. Please introduce more precisely the second model MyeloidA20KO, and discuss the arthritis occuring in these mice (seems not enthesitis driven spA, more ressembling to animal model of RA)"

The A20$^{myelo\text{-}KO}$ model is indeed rather a model for RA, the model was clarified and included in lines 306-313:

"A20$^{myel\text{-}KO}$ mice, generated by crossing floxed-*A20/Tnfaip3* mice with Lysozyme-Cre transgenic mice, which leads to conditional *A20* deletion in the myeloid compartment, were previously shown to develop an erosive TNF-independent but NLRP3 inflammasome- and IL-1β dependent polyarthritis resembling rheumatoid arthritis. In this model, arthritis develops as a result of macrophage necroptosis, which leads to inflammasome activation and the release of IL-1 β and intracellular danger-associated molecular patterns (DAMPs) (Matmati *et al.*, 2011; Walle *et al.*, 2014; Polykratis *et al.*, 2019)"

15th Aug 2023

Dear Prof. Vereecke,

Thank you for the submission of your revised manuscript to EMBO Molecular Medicine. I am pleased to inform you that we will be able to accept your manuscript pending the following final amendments:

1) In the main manuscript file, please do the following:
- Correct/answer the track changes suggested by our data editors by working from the attached document.
- Remove text highlight color.
- Remove "Abbreviations".
- Please rename "Conflict of Interest" to "Disclosure Statement & Competing Interests". We updated our journal's competing interests policy in January 2022 and request authors to consider both actual and perceived competing interests. Please review the policy https://www.embopress.org/competing-interests and update your competing interests if necessary.
- Author contributions: Please remove it from the manuscript and specify author contributions in our submission system. CRediT has replaced the traditional author contributions section because it offers a systematic machine-readable author contributions format that allows for more effective research assessment. You are encouraged to use the free text boxes beneath each contributing author's name to add specific details on the author's contribution. More information is available in our guide to authors:
https://www.embopress.org/page/journal/17574684/authorguide#authorshipguidelines
- Data availability: Please use the following format to report the accession number of your data:

The datasets produced in this study are available in the following databases:
[data type]: [full name of the resource] [accession number/identifier] ([doi or URL or identifiers.org/DATABASE:ACCESSION])

Please check "Author Guidelines" for more information.
https://www.embopress.org/page/journal/17574684/authorguide#availabilityofpublishedmaterial
- Correct the reference citation in the reference list. Where there are more than 10 authors on a paper, 10 will be listed, followed by "et al.". R Please check "Author Guidelines" for more information.
https://www.embopress.org/page/journal/17574684/authorguide#referencesformat
2) EV Figures: Please upload EV Figures as Figure files, not as Expanded View files.
3) Movies: Please remove the legend for EV Movies 1-4 from the main manuscript file. Each movie needs to have a separate legend as a README.txt file zipped together with corresponding movie file and uploaded as a separate zip file. Nomenclature is Movie EV1, etc. also in the main text.
4) Appendix: Please remove all individual Appendix figure files and only leave combined .pdf file.
5) Synopsis:
- Synopsis text: Please remove it from the main manuscript file and upload it as a separate .doc file.
- Synopsis image: Please adjust the size of the synopsis image to 550 px-wide x (250-400)-px high.
- Please check your synopsis text and image before submission with your revised manuscript. Please be aware that in the proof stage minor corrections only are allowed (e.g., typos).
6) For more information: This space should be used to list relevant web links for further consultation by our readers. Could you identify some relevant ones and provide such information as well? Some examples are patient associations, relevant databases, OMIM/proteins/genes links, author's websites, etc...
7) As part of the EMBO Publications transparent editorial process initiative (see our Editorial at http://embomolmed.embopress.org/content/2/9/329), EMBO Molecular Medicine will publish online a Review Process File (RPF) to accompany accepted manuscripts. This file will be published in conjunction with your paper and will include the anonymous referee reports, your point-by-point response and all pertinent correspondence relating to the manuscript. Let us know whether you agree with the publication of the RPF and as here, if you want to remove or not any figures from it prior to publication. Please note that the Authors checklist will be published at the end of the RPF.
8) Please provide a point-by-point letter INCLUDING my comments as well as the reviewer's reports and your detailed responses (as Word file).

I look forward to reading a new revised version of your manuscript as soon as possible.

Yours sincerely,
Zeljko Durdevic

Yours sincerely,

Zeljko Durdevic

Zeljko Durdevic
Editor
EMBO Molecular Medicine

*** Instructions to submit your revised manuscript ***

*** PLEASE NOTE *** As part of the EMBO Publications transparent editorial process initiative (see our Editorial at https://www.embopress.org/doi/pdf/10.1002/emmm.201000094), EMBO Molecular Medicine will publish online a Review Process File to accompany accepted manuscripts.

In the event of acceptance, this file will be published in conjunction with your paper and will include the anonymous referee reports, your point-by-point response and all pertinent correspondence relating to the manuscript. If you do NOT want this file to be published, please inform the editorial office at contact@embomolmed.org.

To submit your manuscript, please follow this link:

https://embomolmed.msubmit.net/cgi-bin/main.plex

When submitting your revised manuscript, please include:

1) a .docx formatted version of the manuscript text (including Figure legends and tables)

2) Separate figure files*

3) supplemental information as Expanded View and/or Appendix. Please carefully check the authors guidelines for formatting Expanded view and Appendix figures and tables at https://www.embopress.org/page/journal/17574684/authorguide#expandedview

4) a letter INCLUDING the reviewer's reports and your detailed responses to their comments (as Word file).

5) The paper explained: EMBO Molecular Medicine articles are accompanied by a summary of the articles to emphasize the major findings in the paper and their medical implications for the non-specialist reader. Please provide a draft summary of your article highlighting
- the medical issue you are addressing,
- the results obtained and
- their clinical impact.
This may be edited to ensure that readers understand the significance and context of the research.
Please refer to any of our published articles for an example.

6) For more information: There is space at the end of each article to list relevant web links for further consultation by our readers. Could you identify some relevant ones and provide such information as well? Some examples are patient associations, relevant databases, OMIM/proteins/genes links, author's websites, etc...

7) Author contributions: the contribution of every author must be detailed in a separate section.

8) EMBO Molecular Medicine now requires a complete author checklist (https://www.embopress.org/page/journal/17574684/authorguide) to be submitted with all revised manuscripts. Please use the checklist as guideline for the sort of information we need WITHIN the manuscript. The checklist should only be filled with page numbers were the information can be found. This is particularly important for animal reporting, antibody dilutions (missing) and exact values and n that should be indicted instead of a range.

9) Every published paper now includes a 'Synopsis' to further enhance discoverability. Synopses are displayed on the journal webpage and are freely accessible to all readers. They include a short stand first (maximum of 300 characters, including space) as well as 2-5 one sentence bullet points that summarise the paper. Please write the bullet points to summarise the key NEW findings. They should be designed to be complementary to the abstract - i.e. not repeat the same text. We encourage inclusion of key acronyms and quantitative information (maximum of 30 words / bullet point). Please use the passive voice. Please attach these in a separate file or send them by email, we will incorporate them accordingly.

You are also welcome to suggest a striking image or visual abstract to illustrate your article. If you do please provide a jpeg file 550 px-wide x 400-px high.

10) A Conflict of Interest statement should be provided in the main text

11) Please note that we now mandate that all corresponding authors list an ORCID digital identifier. This takes <90 seconds to complete. We encourage all authors to supply an ORCID identifier, which will be linked to their name for unambiguous name identification.

Currently, our records indicate that the ORCID for your account is 0000-0002-1593-862X.

Please click the link below to modify this ORCID:
Link Not Available

12) The system will prompt you to fill in your funding and payment information. This will allow Wiley to send you a quote for the article processing charge (APC) in case of acceptance. This quote takes into account any reduction or fee waivers that you may be eligible for. Authors do not need to pay any fees before their manuscript is accepted and transferred to our publisher.

*Additional important information regarding Figures

Each figure should be given in a separate file and should have the following resolution:
Graphs 800-1,200 DPI
Photos 400-800 DPI
Colour (only CMYK) 300-400 DPI"

Figures are not edited by the production team. All lettering should be the same size and style; figure panels should be indicated by capital letters (A, B, C etc). Gridlines are not allowed except for log plots. Figures should be numbered in the order of their appearance in the text with Arabic numerals. Each Figure must have a separate legend and a caption is needed for each panel.

*Additional important information regarding figures and illustrations can be found at https://bit.ly/EMBOPressFigurePreparationGuideline. See also figure legend preparation guidelines: https://www.embopress.org/page/journal/17574684/authorguide#figureformat

The system will prompt you to fill in your funding and payment information. This will allow Wiley to send you a quote for the article processing charge (APC) in case of acceptance. This quote takes into account any reduction or fee waivers that you may be eligible for. Authors do not need to pay any fees before their manuscript is accepted and transferred to our publisher.

***** Reviewer's comments *****

Referee #1 (Remarks for Author):

Is suitable for publication.

Referee #2 (Remarks for Author):

Thank you for your careful and thorough responses to all my questions.

Referee #3 (Remarks for Author):

My concerns have been addressed.

The authors have addressed all minor editorial requests.

22nd Aug 2023

Dear Prof. Vereecke,

We are pleased to inform you that your manuscript is accepted for publication and is now being sent to our publisher to be included in the next available issue of EMBO Molecular Medicine.

Please read below for additional IMPORTANT information regarding your article, its publication and the production process.

Congratulations on your interesting work,

Zeljko Durdevic

Zeljko Durdevic
Editor
EMBO Molecular Medicine

Follow us on Twitter @EmboMolMed
Sign up for eTOCs at embopress.org/alertsfeeds

*** *** *** IMPORTANT INFORMATION *** *** ***

SPEED OF PUBLICATION
The journal aims for rapid publication of papers, using using the advance online publication "Early View" to expedite the process: A properly copy-edited and formatted version will be published as "Early View" after the proofs have been corrected. Please help the Editors and publisher avoid delays by providing e-mail address(es), telephone and fax numbers at which author(s) can be contacted.

Should you be planning a Press Release on your article, please get in contact with embomolmed@wiley.com as early as possible, in order to coordinate publication and release dates.

LICENSE AND PAYMENT:

All articles published in EMBO Molecular Medicine are fully open access: immediately and freely available to read, download and share.

EMBO Molecular Medicine charges an article processing charge (APC) to cover the publication costs. You, as the corresponding author for this manuscript, should have already received a quote with the article processing fee separately. Please let us know in case this quote has not been received.

Once your article is at Wiley for editorial production you will receive an email from Wiley's Author Services system, which will ask you to log in and will present you with the publication license form for completion. Within the same system the publication fee can be paid by credit card, an invoice, pro forma invoice or purchase order can be requested.

Payment of the publication charge and the signed Open Access Agreement form must be received before the article can be published online.

PROOFS

You will receive the proofs by e-mail approximately 2 weeks after all relevant files have been sent o our Production Office. Please return them within 48 hours and if there should be any problems, please contact the production office at embopressproduction@wiley.com.

Please inform us if there is likely to be any difficulty in reaching you at the above address at that time. Failure to meet our

deadlines may result in a delay of publication.

All further communications concerning your paper proofs should quote reference number EMM-2023-17691-V3 and be directed to the production office at embopressproduction@wiley.com.

Thank you,

Zeljko Durdevic
Editor
EMBO Molecular Medicine

EMBO Press Author Checklist

Corresponding Author Name: Lars Vereecke	
Journal Submitted to: EMBO Molecular Medicine	
Manuscript Number: EMM-2023-17691	

USEFUL LINKS FOR COMPLETING THIS FORM
The EMBO Journal - Author Guidelines
EMBO Reports - Author Guidelines
Molecular Systems Biology - Author Guidelines
EMBO Molecular Medicine - Author Guidelines

Reporting Checklist for Life Science Articles (updated January 2022)

This checklist is adapted from Materials Design Analysis Reporting (MDAR) Checklist for Authors. MDAR establishes a minimum set of requirements in transparent reporting in the life sciences (see Statement of Task: 10.31222/osf.io/9sm4x). Please follow the journal's guidelines in preparing your manuscript.

Please note that a copy of this checklist will be published alongside your article.

Abridged guidelines for figures

1. Data

The data shown in figures should satisfy the following conditions:

- the data were obtained and processed according to the field's best practice and are presented to reflect the results of the experiments in an accurate and unbiased manner.
- ideally, figure panels should include only measurements that are directly comparable to each other and obtained with the same assay.
- plots include clearly labeled error bars for independent experiments and sample sizes. Unless justified, error bars should not be shown for technical replicates.
- if n<5, the individual data points from each experiment should be plotted. Any statistical test employed should be justified.
- Source Data should be included to report the data underlying figures according to the guidelines set out in the authorship guidelines on Data Presentation.

2. Captions

Each figure caption should contain the following information, for each panel where they are relevant:

- a specification of the experimental system investigated (eg cell line, species name).
- the assay(s) and method(s) used to carry out the reported observations and measurements.
- an explicit mention of the biological and chemical entity(ies) that are being measured.
- an explicit mention of the biological and chemical entity(ies) that are altered/varied/perturbed in a controlled manner.
- the exact sample size (n) for each experimental group/condition, given as a number, not a range;
- a description of the sample collection allowing the reader to understand whether the samples represent technical or biological replicates (including how many animals, litters, cultures, etc.).
- a statement of how many times the experiment shown was independently replicated in the laboratory.
- definitions of statistical methods and measures:
  - common tests, such as t-test (please specify whether paired vs. unpaired), simple χ2 tests, Wilcoxon and Mann-Whitney tests, can be unambiguously identified by name only, but more complex techniques should be described in the methods section;
  - are tests one-sided or two-sided?
  - are there adjustments for multiple comparisons?
  - exact statistical test results, e.g., P values = x but not P values < x;
  - definition of 'center values' as median or average;
  - definition of error bars as s.d. or s.e.m.

Please complete ALL of the questions below.
Select "Not Applicable" only when the requested information is not relevant for your study.

Materials

Newly Created Materials	Information included in the manuscript?	In which section is the information available? (Reagents and Tools Table, Materials and Methods, Figures, Data Availability Section)
New materials and reagents need to be available; do any restrictions apply?	Not Applicable	

Antibodies	Information included in the manuscript?	In which section is the information available? (Reagents and Tools Table, Materials and Methods, Figures, Data Availability Section)
For **antibodies** provide the following information:<br>- Commercial antibodies: RRID (if possible) or supplier name, catalogue number and or/clone number<br>- Non-commercial: RRID or citation	Yes	Materials and Methods

DNA and RNA sequences	Information included in the manuscript?	In which section is the information available? (Reagents and Tools Table, Materials and Methods, Figures, Data Availability Section)
**Short novel DNA or RNA including primers, probes**: provide the sequences.	Yes	Materials and Methods, Figure EV1

Cell materials	Information included in the manuscript?	In which section is the information available? (Reagents and Tools Table, Materials and Methods, Figures, Data Availability Section)
**Cell lines:** Provide species information, strain. Provide accession number in repository **OR** supplier name, catalog number, clone number, and/**OR** RRID.	Not Applicable	
**Primary cultures:** Provide species, strain, sex of origin, genetic modification status.	Not Applicable	Materials and Methods
Report if the cell lines were recently **authenticated** (e.g., by STR profiling) and tested for mycoplasma contamination.	Not Applicable	

Experimental animals	Information included in the manuscript?	In which section is the information available? (Reagents and Tools Table, Materials and Methods, Figures, Data Availability Section)
**Laboratory animals or Model organisms:** Provide species, strain, sex, age, genetic modification status. Provide accession number in repository **OR** supplier name, catalog number, clone number, **OR** RRID.	Yes	Materials and Methods
**Animal observed in or captured from the field:** Provide species, sex, and age where possible.	Not Applicable	
Please detail **housing and husbandry conditions**.	Yes	Materials and Methods

Plants and microbes	Information included in the manuscript?	In which section is the information available? (Reagents and Tools Table, Materials and Methods, Figures, Data Availability Section)
**Plants:** provide species and strain, ecotype and cultivar where relevant, unique accession number if available, and source (including location for collected wild specimens).	Not Applicable	
**Microbes:** provide species and strain, unique accession number if available, and source.	Not Applicable	

Human research participants	Information included in the manuscript?	In which section is the information available? (Reagents and Tools Table, Materials and Methods, Figures, Data Availability Section)
If collected and within the bounds of privacy constraints report on age, sex and gender or ethnicity for all study participants.	Not Applicable	

Core facilities	Information included in the manuscript?	In which section is the information available? (Reagents and Tools Table, Materials and Methods, Figures, Data Availability Section)
If your work benefited from core facilities, was their service mentioned in the acknowledgments section?	Yes	Acknowledgements section

Design

Study protocol	Information included in the manuscript?	In which section is the information available? (Reagents and Tools Table, Materials and Methods, Figures, Data Availability Section)
If study protocol has been **pre-registered, provide DOI in the manuscript**. For clinical trials, provide the trial registration number **OR** cite DOI.	Not Applicable	
Report the **clinical trial registration number** (at ClinicalTrials.gov or equivalent), where applicable.	Not Applicable	

Laboratory protocol	Information included in the manuscript?	In which section is the information available? (Reagents and Tools Table, Materials and Methods, Figures, Data Availability Section)
Provide DOI OR other citation details if **external detailed step-by-step protocols** are available.	Yes	Materials and Methods

Experimental study design and statistics	Information included in the manuscript?	In which section is the information available? (Reagents and Tools Table, Materials and Methods, Figures, Data Availability Section)
Include a statement about **sample size** estimate even if no statistical methods were used.	Yes	
Were any steps taken to minimize the effects of subjective bias when allocating animals/samples to treatment (e.g. **randomization procedure**)? If yes, have they been described?	Yes	Mice were randomized in all experiments
Include a statement about **blinding** even if no blinding was done.	Yes	
Describe **inclusion/exclusion criteria** if samples or animals were excluded from the analysis. Were the criteria pre-established?<br><br>If sample or data points were omitted from analysis, report if this was due to attrition or intentional exclusion and provide justification.	Not Applicable	
For every figure, are **statistical tests** justified as appropriate? Do the data meet the assumptions of the tests (e.g., normal distribution)? Describe any methods used to assess it. Is there an estimate of variation within each group of data? Is the variance similar between the groups that are being statistically compared?	Yes	Figure legends, Materials and Methods

Sample definition and in-laboratory replication	Information included in the manuscript?	In which section is the information available? (Reagents and Tools Table, Materials and Methods, Figures, Data Availability Section)
In the figure legends: state number of times the experiment was **replicated** in laboratory.	Yes	
In the figure legends: define whether data describe **technical or biological replicates**.	Yes	Figure legends

Ethics

Ethics	Information included in the manuscript?	In which section is the information available? (Reagents and Tools Table, Materials and Methods, Figures, Data Availability Section)
Studies involving **human participants**: State details of **authority granting ethics approval** (IRB or equivalent committee(s), provide reference number for approval.	Not Applicable	
Studies involving **human participants**: Include a statement confirming that **informed consent** was obtained from all subjects and that the experiments conformed to the principles set out in the WMA Declaration of Helsinki and the Department of Health and Human Services Belmont Report.	Not Applicable	
Studies involving **human participants:** For publication of **patient photos**, include a statement confirming that consent to publish was obtained.	Not Applicable	
Studies involving experimental **animals**: State details of **authority granting ethics approva**l (IRB or equivalent committee(s), provide reference number for approval. Include a statement of compliance with ethical regulations.	Yes	Materials and Methods
Studies involving **specimen and field samples:** State if relevant **permits** obtained, provide details of authority approving study; if none were required, explain why.	Not Applicable	

Dual Use Research of Concern (DURC)	Information included in the manuscript?	In which section is the information available? (Reagents and Tools Table, Materials and Methods, Figures, Data Availability Section)
Could your study fall under dual use research restrictions? Please check biosecurity documents and list of **select agents and toxins** (CDC): https://www.selectagents.gov/sat/list.htm	Not Applicable	
If you used a select agent, is the security level of the lab appropriate and reported in the manuscript?	Not Applicable	
If a study is subject to dual use research of concern regulations, is the name of the **authority granting approval and reference number** for the regulatory approval provided in the manuscript?	Not Applicable	

Reporting

The MDAR framework recommends adoption of discipline-specific guidelines, established and endorsed through community initiatives. Journals have their own policy about requiring specific guidelines and recommendations to complement MDAR.

Adherence to community standards	Information included in the manuscript?	In which section is the information available? (Reagents and Tools Table, Materials and Methods, Figures, Data Availability Section)
State if relevant guidelines or checklists (e.g., **ICMJE, MIBBI, ARRIVE, PRISMA**) have been followed or provided.	Yes	
For **tumor marker prognostic studies,** we recommend that you follow the **REMARK** reporting guidelines (see link list at top right). See author guidelines, under 'Reporting Guidelines'. Please confirm you have followed these guidelines.	Not Applicable	
For **phase II and III randomized controlled trials**, please refer to the **CONSORT** flow diagram (see link list at top right) and submit the CONSORT checklist (see link list at top right) with your submission. See author guidelines, under 'Reporting Guidelines'. Please confirm you have submitted this list.	Not Applicable	

Data Availability

Data availability	Information included in the manuscript?	In which section is the information available? (Reagents and Tools Table, Materials and Methods, Figures, Data Availability Section)
Have **primary datasets** been deposited according to the journal's guidelines (see 'Data Deposition' section) and the respective accession numbers provided in the Data Availability Section?	Yes	
Were **human clinical and genomic datasets** deposited in a public access-controlled repository in accordance to ethical obligations to the patients and to the applicable consent agreement?	Not Applicable	
Are **computational models** that are central and integral to a study available without restrictions in a machine-readable form? Were the relevant accession numbers or links provided?	Not Applicable	
If publicly available data were reused, provide the respective **data citations in the reference list**.	Not Applicable	
